# Supplementary material for: Learning mutational signatures and their multidimensional genomic properties with TensorSignatures
Source: Nat Commun. 2021 Jun 15;12:3628. doi: 10.1038/s41467-021-23551-9 (PMC8206343; doi:10.1038/s41467-021-23551-9)
Supplement: Supplementary file 1 — Supplementary Information [file 41467_2021_23551_MOESM1_ESM.pdf]

Learning mutational signatures and their multidimensional genomic properties with TensorSignatures

Harald Vöhringer[1], Arne Van Hoeck[2], Edwin Cuppen[2,3], and Moritz Gerstung[1,4,*]

[1] *European Molecular Biology Laboratory, European Bioinformatics Institute (EMBL-EBI), Hinxton, UK.*
[2] *Center for Molecular Medicine and Oncode Institute, University Medical Center Utrecht, Universiteitsweg 100, 3584 CG Utrecht, The Netherlands.*
[3] *Hartwig Medical Foundation, Amsterdam, The Netherlands.*
[4] *European Molecular Biology Laboratory, Genome Biology Unit, Heidelberg, Germany.*
[*] *to whom correspondence should be addressed*

Abstract

Mutational signature analysis is an essential part of the cancer genome analysis toolkit. Here we present TensorSignatures, an algorithm to learn mutational signatures jointly across different variant categories as well as their genomic localisation and properties. The analysis of 2,778 primary and 3,824 metastatic cancer genomes of the PCAWG consortium and the HMF cohort shows that practically all signatures operate dynamically in response to various genomic and epigenomic states. The analysis pins differential spectra of UV mutagenesis found in active and inactive chromatin to global genome nucleotide excision repair. TensorSignatures accurately characterises transcription-associated mutagenesis, which is detected in 7 different cancer types. The algorithm also extracts distinct signatures of replication- and double strand break repair-driven mutagenesis by APOBEC3A and 3B with differential numbers and length of mutation clusters. As a fourth example, TensorSignatures reproduces a signature of somatic hypermutation generating highly clustered variants around the transcription start sites of active genes in lymphoid leukaemia, distinct from a more general and less clustered signature of Polη-driven translesion synthesis found in a broad range of cancer types.

Contents

1 Supplementary Figures **4**

Supplementary Figure 1: Simulation Experiments 4

Supplementary Figure 2: Extracting the genomic properties of mutational signatures by regression. 6

Supplementary Figure 3: Assigning single base substitutions to their source signature with maximum a posteriori approaches. 8

Supplementary Figure 4: Stability of solutions. 9

Supplementary Figure 5: Model selection in the PCAWG dataset. 10

Supplementary Figure 6: Extracting mutational signatures in the PCAWG dataset with SigProfiler. 11

Supplementary Figure 7: Extracting epigenetic signature activities with partially matched ChromHMM annotations. 12

Supplementary Figure 8: Validation of TensorSignatures in the HMF cohort. 13

Supplementary Figure 9: The spectrum of UV mutagenesis changes from closed to open chromatin, reflecting GG- and TC-NER. 14

Supplementary Figure 10: Transcription-associated mutagenesis is common in highly transcribed genes. 16

Supplementary Figure 11: Replication- and DSBR-driven mutagenesis by APOBEC3A and 3B. 17

Supplementary Figure 12: Targeted somatic hypermutation at TSS and dispersed clustered translesion synthesis. 18

2 Supplementary Note 1: TensorSignature Vignettes **19**

TS01-N[C>T]G (5meC>T) 19

TS02-N[C>T]N (unknown) 21

TS03-N[N>N]N-q (unknown/quiet) 23

TS04-N[N>N]N (unknown/active) 25

TS05-T[C>T]N (UV/GG-NER) 27

TS06-Y[C>T]N (UV/GG+TC-NER) 29

TS07-N[T>C]N (unknown) 31

TS08-A[T>C]W (unknown/TAM) 33

TS09-N[T>A]N (PAH/AA) 35

TS10-N[C>A]N (PAH/B[a]P) 37

TS11-T[C>D]W;SV (APOBEC) 39

TS12-T[C>D]W (APOBEC) 41

TS13-N[C>K]H (AID/SHM) 43
TS14-W[T>V]W (POLH) 45
TS15-G[C>T]N;ID (MMRD) 47
TS16-N[C>A]T;ID (MMRD:POLE-exo) 49
TS17-T[C>A]T (POLE-exo) 51
TS18-N[C>A]W (BERD/MUTYH) 53
TS19-N[N>N]N;SV (HRD/BRCA) 55
TS20-N[T>G]T (unknown/5FU) 57

3 Supplementary Note 2: TensorSignatures Manual **59**

Installing TensorSignatures 59
Installation via GitHub 59
Installation via Pypi 59
Installation via Docker 60
Quick Start 60
Step 1: Data preparation 60
Step 2: Computing trinucleotide normalisation 61
Step 3: Run TensorSignatures 61
Tutorials 62
Understanding the mutation count tensor 62
Understanding tensor factors 65
The TensorSignatures CLI 68
The TensorSignatures API 70

1 Supplementary Figures

Supplementary Figure 1: Simulation Experiments

Supplementary Fig. 1: Data was simulated to assess the ability to extract tensor signatures. Simulation parameters were chosen such that the average number of mutations per genome (m) ranged between 50 and 10,000, and the number of samples n varied between 10 and 3,000. Ten different realisations of the data were created by perturbing the resulting counts ten times with negative binomial noise ($\tau = 50$). Each tensor factorisation was performed 10 times on each realisation and the solutions which minimised the negative log-likelihood were selected. All models were trained for 50,000 epochs using an ADAMgrad optimiser and a constant learning rate of 0.1. Shown panels report means (bar, dots) and standard deviations (errorbars). **a,** Accuracy of signature inference with respect to the number of samples (n) and the number of mutations per sample (m) in the simulated dataset. Signature recognition is defined as 1 minus cosine distance of the inferred and true signature. **b,** Accuracy of exposure inference with respect to the number of samples (n) and the number of mutations per sample (m) in the simulated dataset. **c,** Accuracy of inferred transcriptional and replicational activities ($a0$) and strand biases ($b0$), and SNV composition ($m1$) with respect to the number of samples (n), and the number of mutations per sample (m) in the simulated dataset. **d,** Accuracy of inferred epigenetic ($k0$) and nucleosomal activities ($k1$), and clustering propensities ($k2$) with respect to the number of samples (n) and the number of mutations per sample (m) in the simulated dataset. **e,** Accuracy of signature recognition at different ranks with respect to sample size (n) and number of mutations (m). **f,** Model selection via BIC. Data was simulated with ten signatures and ten decompositions were performed for ranks 2 to 20. The midline of each boxplot indicates the median, boxes the quartiles of the data, and whiskers extend to 1.5 of the interquartile range, except for points that indicate outliers.

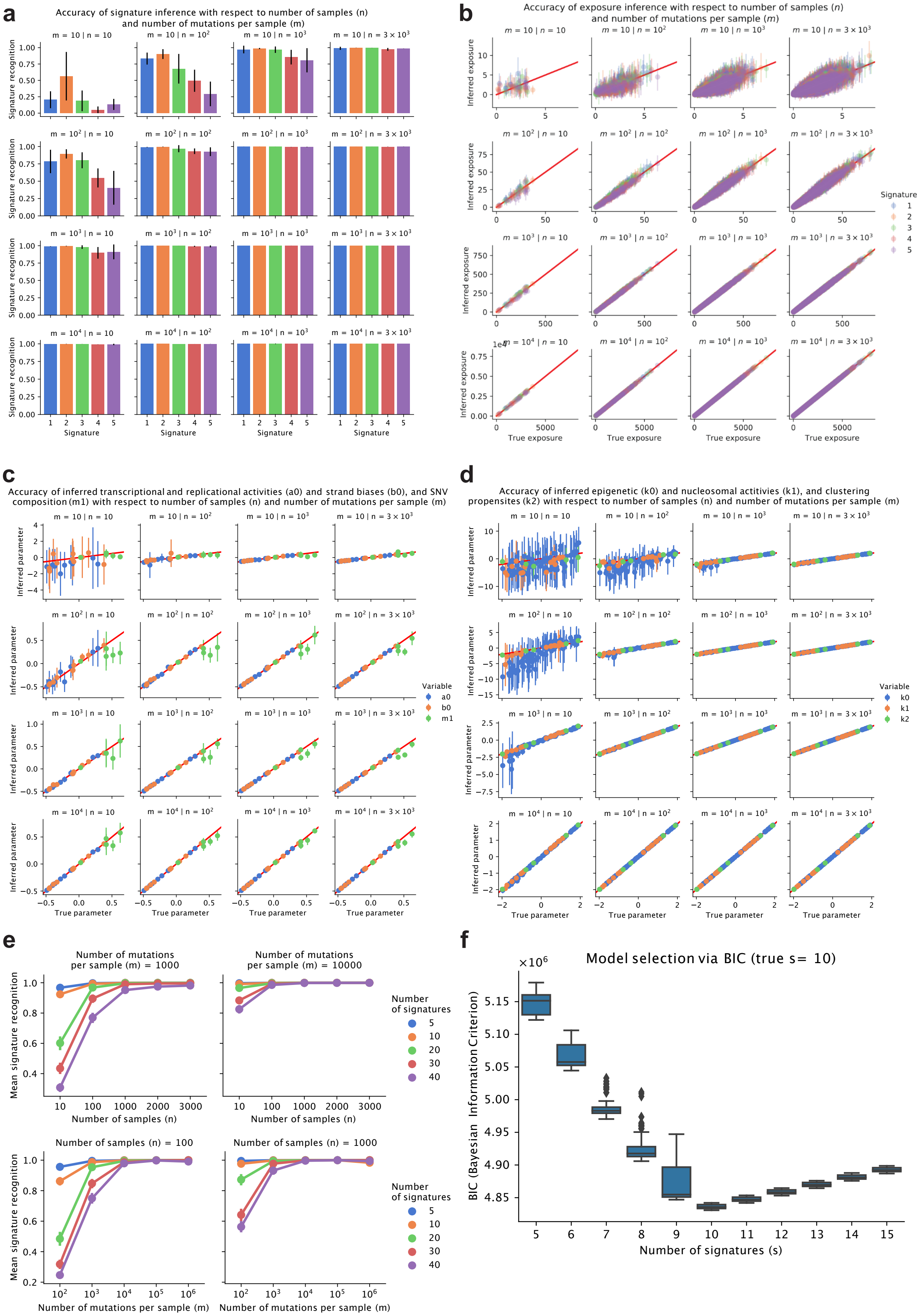
a
Accuracy of signature inference with respect to number of samples (n) and number of mutations per sample (m)
Signature recognition
Signature
b
Accuracy of exposure inference with respect to number of samples (n) and number of mutations per sample (m)
Inferred exposure
True exposure
Signature
c
Accuracy of inferred transcriptional and replicational activities (a0) and strand biases (b0), and SNV composition (m1) with respect to number of samples (n) and number of mutations per sample (m)
Inferred parameter
True parameter
Variable
a0
b0
m1
d
Accuracy of inferred epigenetic (k0) and nucleosomal activities (k1), and clustering propensites (k2) with respect to number of samples (n) and number of mutations per sample (m)
Inferred parameter
True parameter
Variable
k0
k1
k2
e
Number of mutations per sample (m) = 1000
Number of mutations per sample (m) = 10000
Mean signature recognition
Number of samples (n)
Number of signatures
Number of samples (n) = 100
Number of samples (n) = 1000
Number of mutations per sample (m)
f
Model selection via BIC (true s= 10)
BIC (Bayesian Information Criterion)
Number of signatures (s)

Supplementary Figure 2: Extracting the genomic properties of mutational signatures by regression.

Supplementary Fig. 2: **Upper panel:** Schematic depiction of the simulation approach to recover signature activities similar to TensorSignatures by regressing activities in different genomic states post-hoc. A mutation count tensor with various genomic features was simulated. To recover mutational signatures and corresponding sample exposures, the marginalised (summed) count tensor was decomposed on the 96-trinucleotide and sample dimension (conventional NMF). To determine strand biases and signature activities across genomic states, exposures of extracted spectra were fitted to the count matrices containing the single base substitutions of a specific state only (e.g. template strand mutations, TssA). To obtain scalar parameters similar to TensorSignatures' tensor factors, state specific exposures were regressed to respective baseline exposures (e.g. exposures of template strand mutations against exposures of unassigned mutations), and obtained regression coefficients compared with equivalent parameters of a tensor factorisation and the ground truth. These experiment were performed for datasets with $n = \{100, 1000, 10000\}$ and different numbers of mutations per sample $m = \{100, 1000, 10000\}$. Note that this approach fails to recover signature activities in untranscribed and transcribed, and early and late replicating regions (indicated as "Amplitudes" in the figure). **Middle and lower panel:** Depiction of relative and absolute errors, respectively. Each boxplot shows the data distribution from 10 initialisations. The midline of each boxplot indicates the median, boxes the quartiles of the data, and whiskers extend to 1.5 of the interquartile range, except for points that indicate outliers.

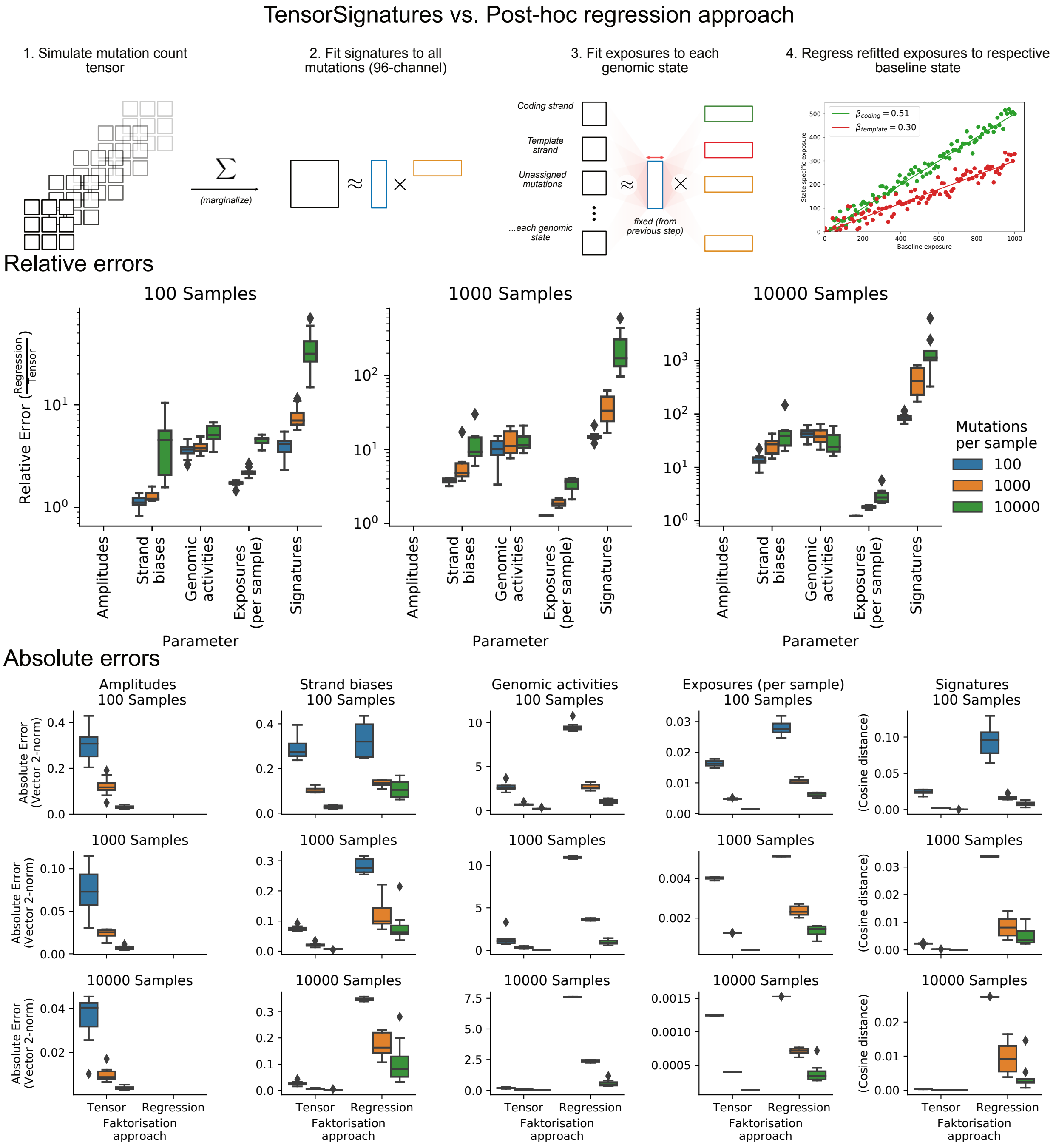
TensorSignatures vs. Post-hoc regression approach
1. Simulate mutation count tensor
(marginalize)
2. Fit signatures to all mutations (96-channel)
3. Fit exposures to each genomic state
Coding strand
Template strand
Unassigned mutations
...each genomic state
fixed (from previous step)
4. Regress refitted exposures to respective baseline state
$\beta_{coding} = 0.51$
$\beta_{template} = 0.30$
State specific exposure
Baseline exposure
Relative errors
100 Samples
1000 Samples
10000 Samples
Relative Error ($\frac{\text{Regression}}{\text{Tensor}}$)
Amplitudes
Strand biases
Genomic activities
Exposures (per sample)
Signatures
Parameter
Mutations per sample
100
1000
10000
Absolute errors
Amplitudes 100 Samples
Strand biases 100 Samples
Genomic activities 100 Samples
Exposures (per sample) 100 Samples
Signatures 100 Samples
1000 Samples
10000 Samples
Absolute Error (Vector 2-norm)
(Cosine distance)
Tensor
Regression
Faktorisation approach

Supplementary Figure 3: Assigning single base substitutions to their source signature with maximum a posteriori approaches.

Supplementary Fig. 3: **Upper panel:** Schematic depiction of the simulation approach which uses maximum a posteriori (MAP) estimation to assign mutations to their respective mutational signature as described by Morganella et al (2016). Two very similar signatures (TS05 and TS06) were used to simulate a mutation count tensor. Conventional NMF was applied to the marginalised (summed) count tensor and subsequently the MAP signature calculated for each mutation type in each sample. **Lower panel:** Per sample absolute errors of the tensor factorisation and the aforementioned strategy. Each boxplot shows the data distribution from 10 initialisations. The midline of each boxplot indicates the median, boxes the quartiles of the data, and whiskers extend to 1.5 of the interquartile range, except for points that indicate outliers.

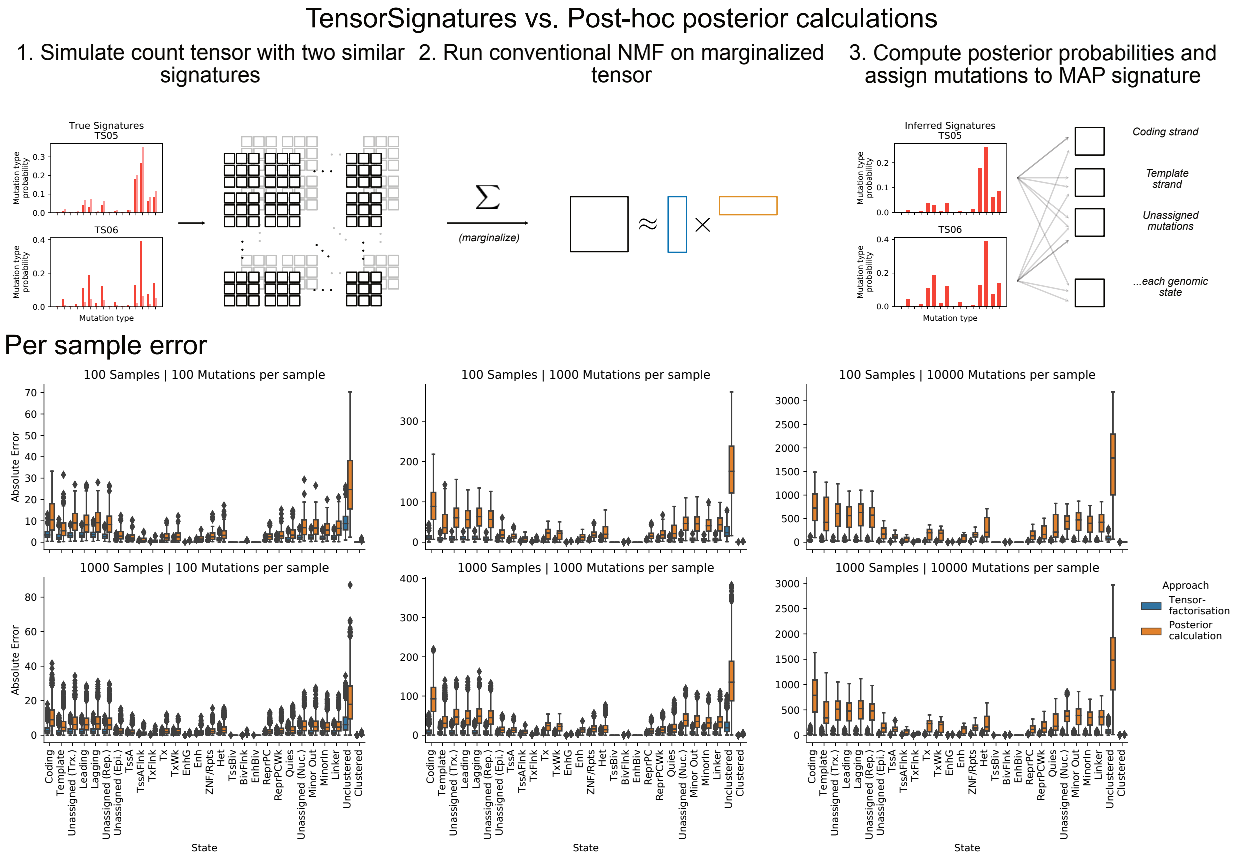

Supplementary Figure 4: Stability of solutions.

Supplementary Fig. 4: **Left panel:** Matching SNV and other mutation type spectra (from independent NMFs) by correlating their exposures. Independent NMFs were performed with SNV and other mutation type count matrices of the PCAWG dataset. To match resulting spectra, correlation coefficients of exposures were calculated, and highest correlating SNV and other mutation type spectra paired. To evaluate the stability of matched signatures and equivalent tensor signatures silhouettes scores were computed. **Right panel:** Signature stability across several ranks using TensorSignatures and the signature matching approach. Dots and error bars indicate the mean and stand deviation of silhouette scores derived from 50 initialisations of post-hoc paired signatures (SNV and other mutation types) and TensorSignatures' signatures.

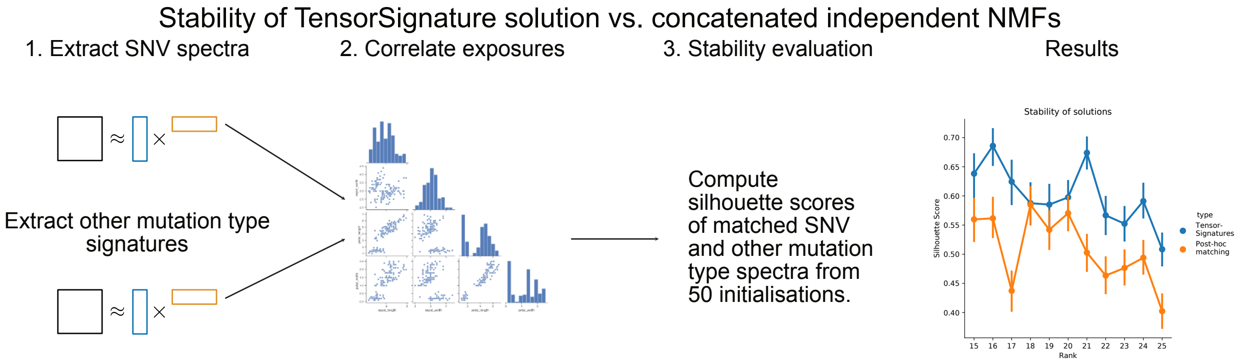

Supplementary Figure 5: Model selection in the PCAWG dataset.

Supplementary Fig. 5: Model selection in the PCAWG dataset (chosen number of signatures 20 with a size τ of 50). Each boxplot shows the data distribution from 50 initialisations. The midline of each boxplot indicates the median, boxes the quartiles of the data, and whiskers extend to 1.5 of the interquartile range, except for points that indicate outliers.

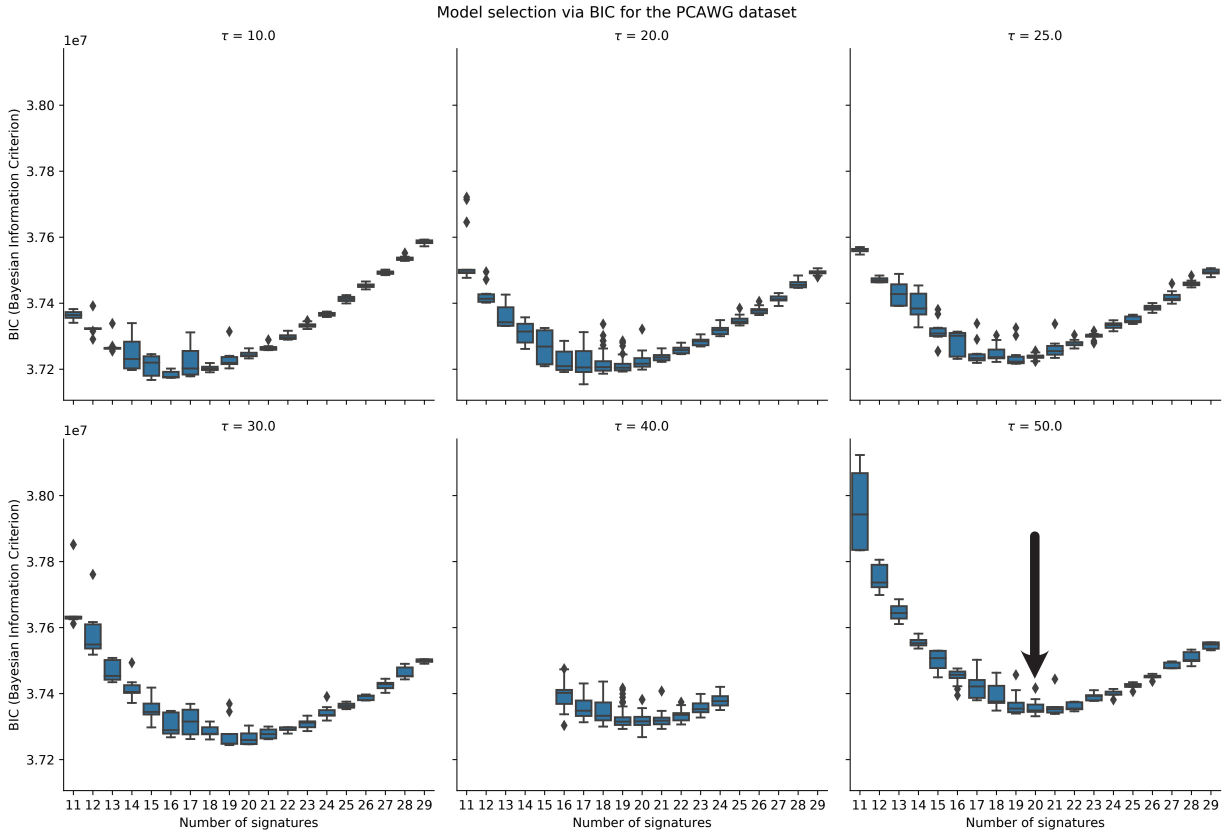

Supplementary Figure 6: Extracting mutational signatures in the PCAWG dataset with SigProfiler.

Supplementary Fig. 6: SigProfiler decomposition of the PCAWG dataset. Decomposition were performed across the ranks 11 to 30 by running the tool with default settings. The heatmap depicts cosine distances between SigProfiler signatures extracted at different decomposition ranks (Suggested rank by SigProfiler: 22, highlighted in green) and closest COSMIC SBS signatures (y-axis).

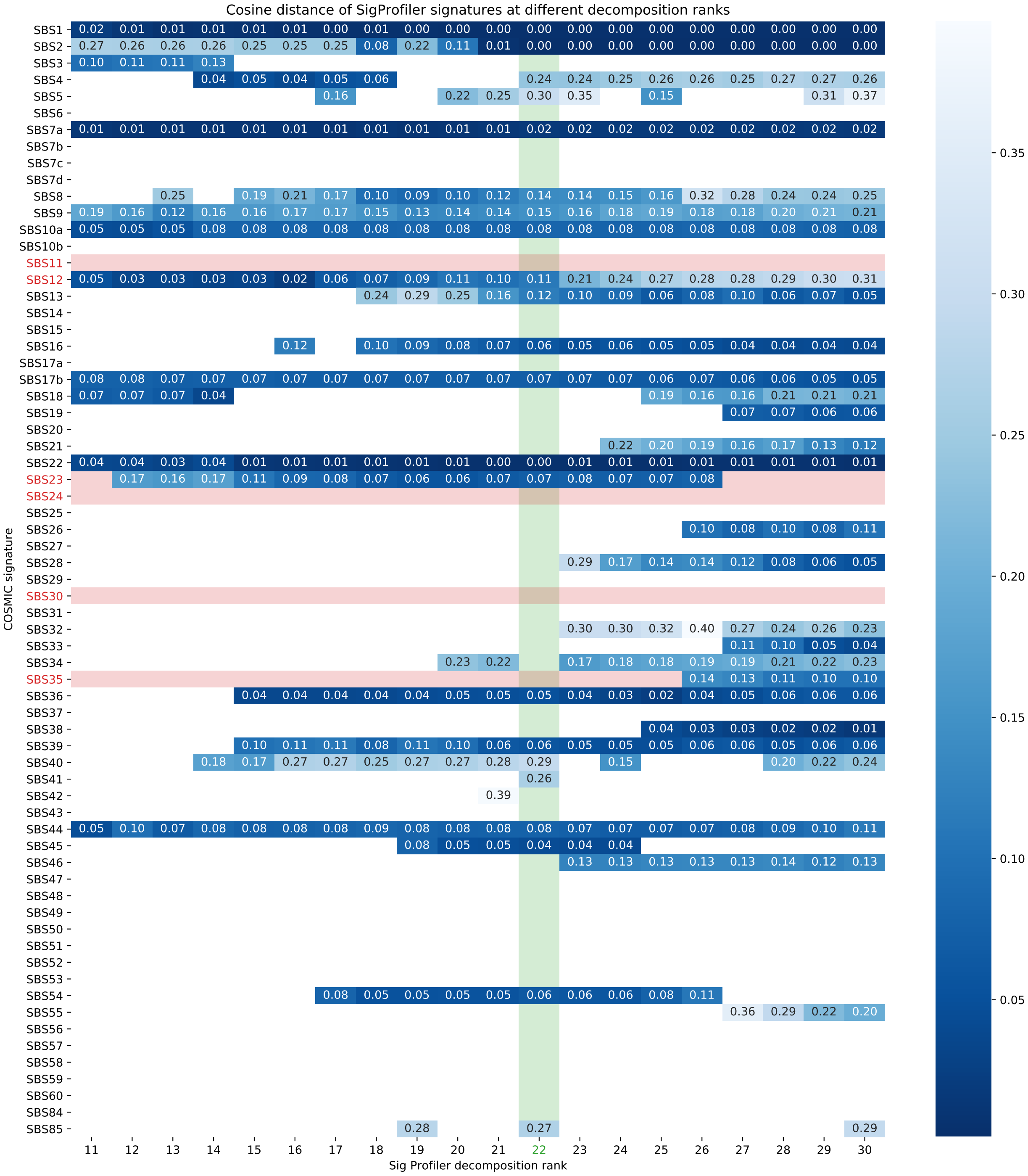

Supplementary Figure 7: Extracting epigenetic signature activities with partially matched ChromHMM annotations.

Supplementary Fig. 7: **a,** Distribution of PCAWG SNV count data across ChromHMM states using an all tissue and tissue specific consensus. **b,** Extracting epigenetic signature activities on partially matched ChromHMM annotations. Comparison of inferred epigenetic signature activities using an all tissue (upper triangle) and tissue specific (lower triangle) consensus (grey triangles indicate NA values).

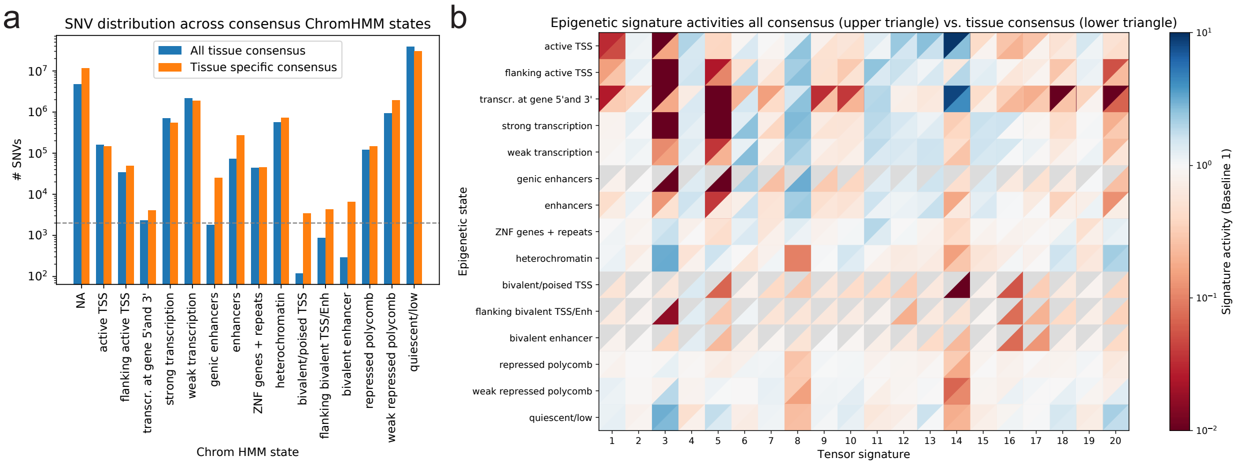

Supplementary Figure 8: Validation of TensorSignatures in the HMF cohort.

Supplementary Fig. 8: **a,** Model selection in the HMF dataset (chosen number of signatures 27 with a size of 30). Each boxplot shows the data distribution from 50 initialisations. The midline of each boxplot indicates the median, boxes the quartiles of the data, and whiskers extend to 1.5 of the interquartile range, except for points that indicate outliers. **b,** Squared errors of tensor factors from the PCAWG discovery and HMF validation analysis. **c,** C>T mutation type probabilities of TS22 for coding and template strand DNA, and the MNV spectrum of TS23.

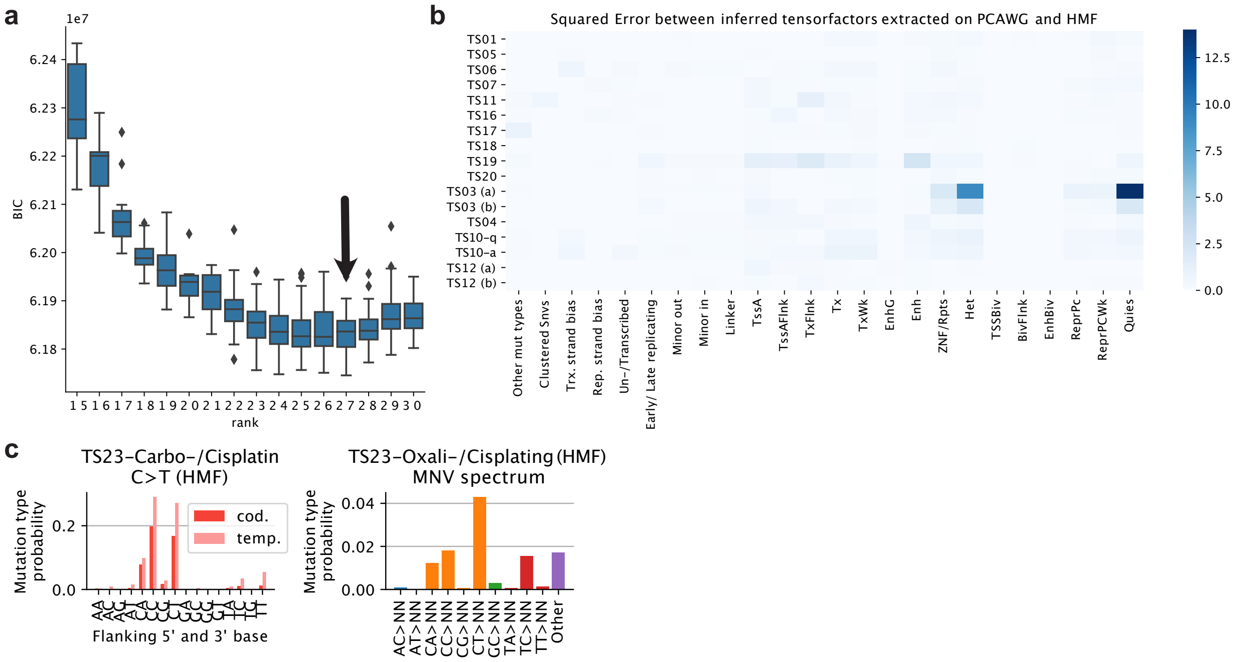

Supplementary Figure 9: The spectrum of UV mutagenesis changes from closed to open chromatin, reflecting GG- and TC-NER.

Supplementary Fig. 9: **a,** Correlation of TS05 and TS06 exposures in PCAWG Skin-Melanoma samples (n=107). **b,** Heptanucleotide context normalised C>T mutation counts in active and quiescent genomic regions. **c,** Pooled C>T variants from cSCC $XPC^{-/-}$ and cSCC XPC^{wt} genomes from active and quiescent regions respectively. Transcriptional strand bias and C[C>T]/T[C>T] spectral shift in GG-NER deficient XPC^{wt} cSCC genomes.

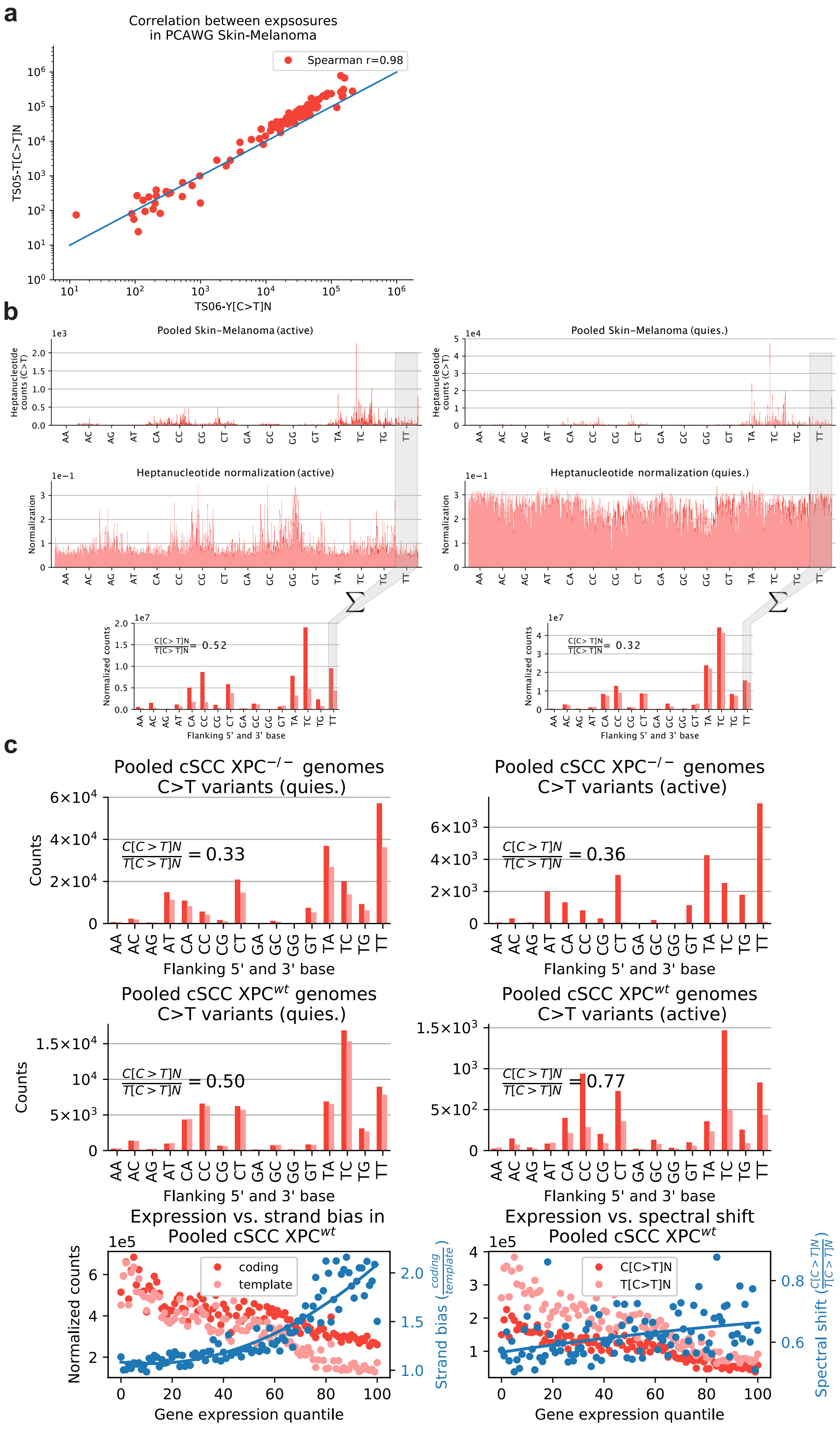

a
Correlation between expsosures in PCAWG Skin-Melanoma
Spearman r=0.98
TS05-T[C>T]N
TS06-Y[C>T]N
b
Pooled Skin−Melanoma (active)
Pooled Skin−Melanoma (quies.)
Heptanucleotide counts (C>T)
Heptanucleotide normalization (active)
Heptanucleotide normalization (quies.)
Normalization
Normalized counts
C[C>T]N / T[C>T]N = 0.52
C[C>T]N / T[C>T]N = 0.32
Flanking 5' and 3' base
c
Pooled cSCC XPC$^{-/-}$ genomes C>T variants (quies.)
Pooled cSCC XPC$^{-/-}$ genomes C>T variants (active)
Counts
C[C>T]N / T[C>T]N = 0.33
C[C>T]N / T[C>T]N = 0.36
Flanking 5' and 3' base
Pooled cSCC XPC^{wt} genomes C>T variants (quies.)
Pooled cSCC XPC^{wt} genomes C>T variants (active)
C[C>T]N / T[C>T]N = 0.50
C[C>T]N / T[C>T]N = 0.77
Expression vs. strand bias in Pooled cSCC XPC^{wt}
coding
template
Normalized counts
Strand bias (coding / template)
Gene expression quantile
Expression vs. spectral shift Pooled cSCC XPC^{wt}
C[C>T]N
T[C>T]N
Spectral shift (C[C>T]N / T[C>T]N)

Supplementary Figure 10: Transcription-associated mutagenesis is common in highly transcribed genes.

Supplementary Fig. 10: **a**, Correlation of predicted TS07 and TS08 mutation counts in PCAWG Liver-HCC samples (n=326). **b**, T>C mutation counts from active genomic regions in samples with high TS08 activity (other than Liver-HCC).

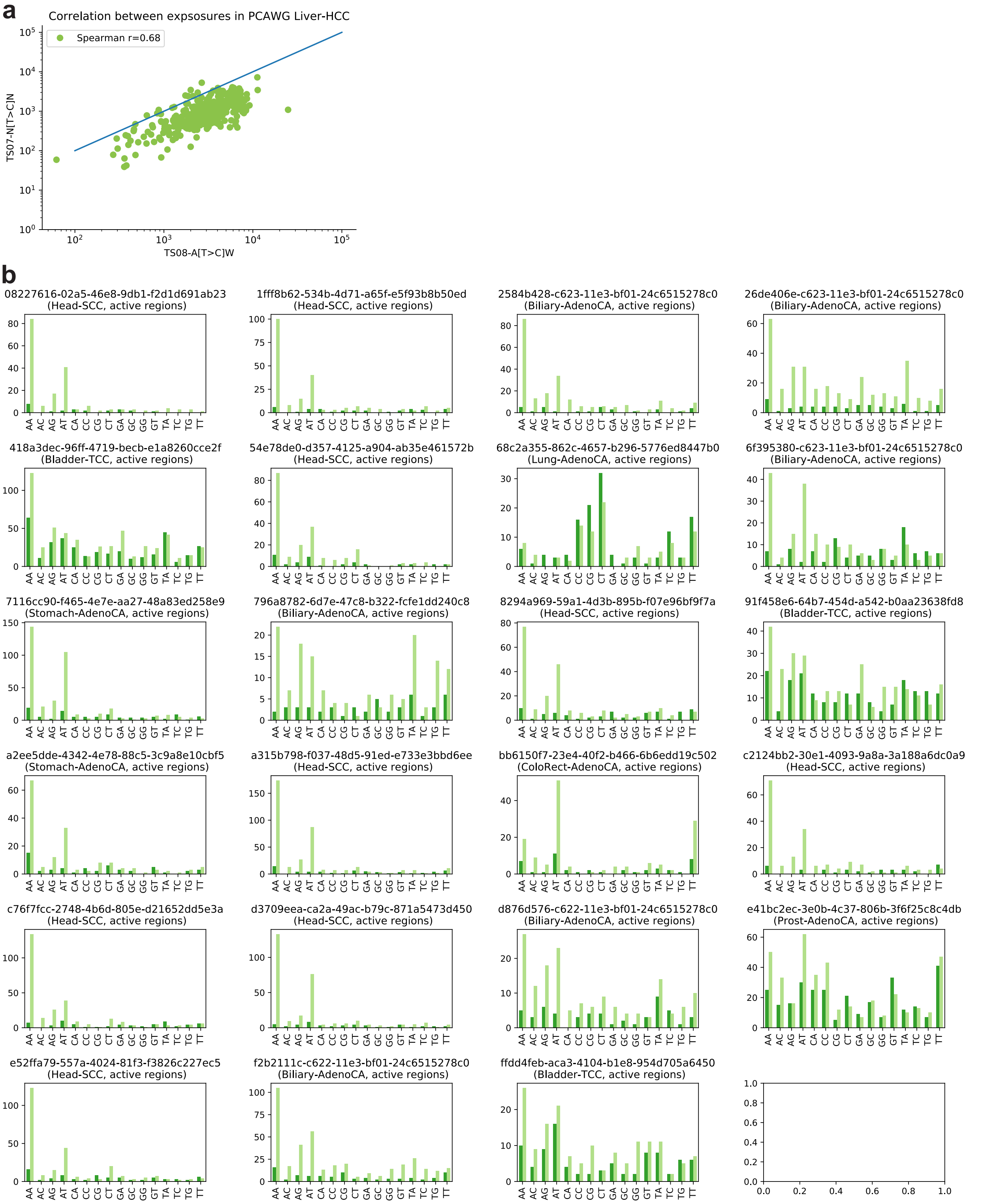

Supplementary Figure 11: Replication- and DSBR-driven mutagenesis by APOBEC3A and 3B.

Supplementary Fig. 11: **a,** Pancancer-wide pooled clustered C>G and C>T variants proximal and distal to SVs **b,** Motif logo plots of the tetranucleotide context at mutated C (top), TC (middle) and TCA (lower) sites in yeast cells exposed to APOBEC3A/3B mutagenesis respectively (Chan et. al. (2017)), and similar motif logo plots extracted at clustered mutations from samples with high TS11 or TS12 exposures.

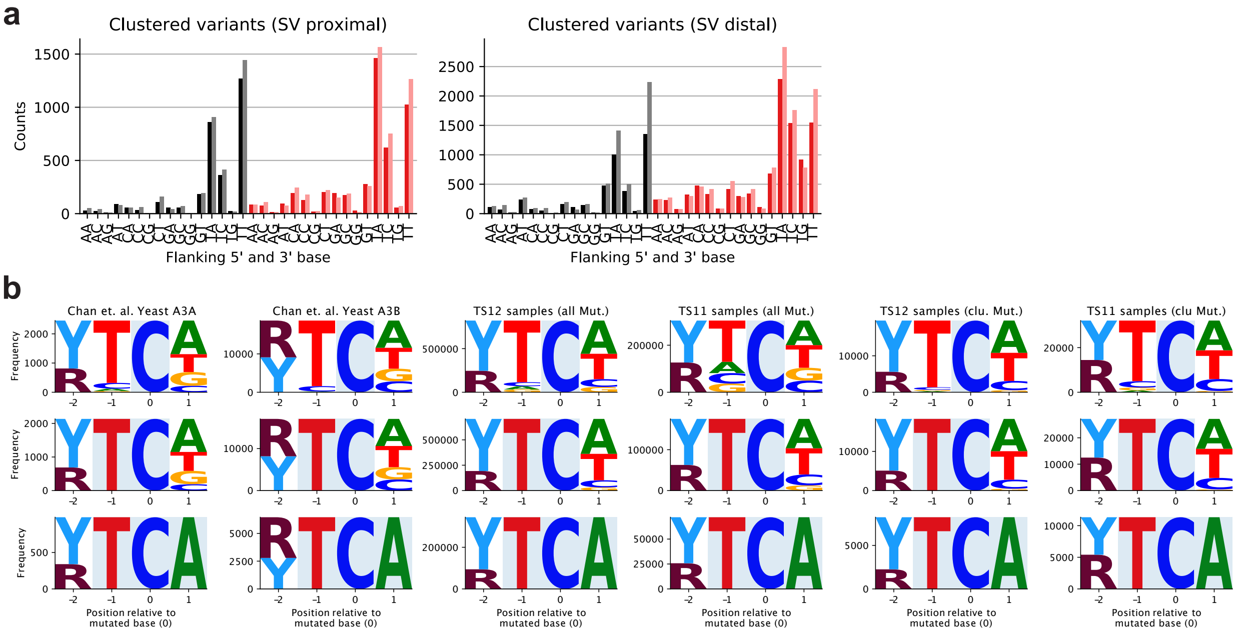

Supplementary Figure 12: Targeted somatic hypermutation at TSS and dispersed clustered translesion synthesis.

Supplementary Fig. 12: Correlation of TS13 and TS14 exposures in lymphoid cancers (Lymph-BNHL/CLL/NOS).

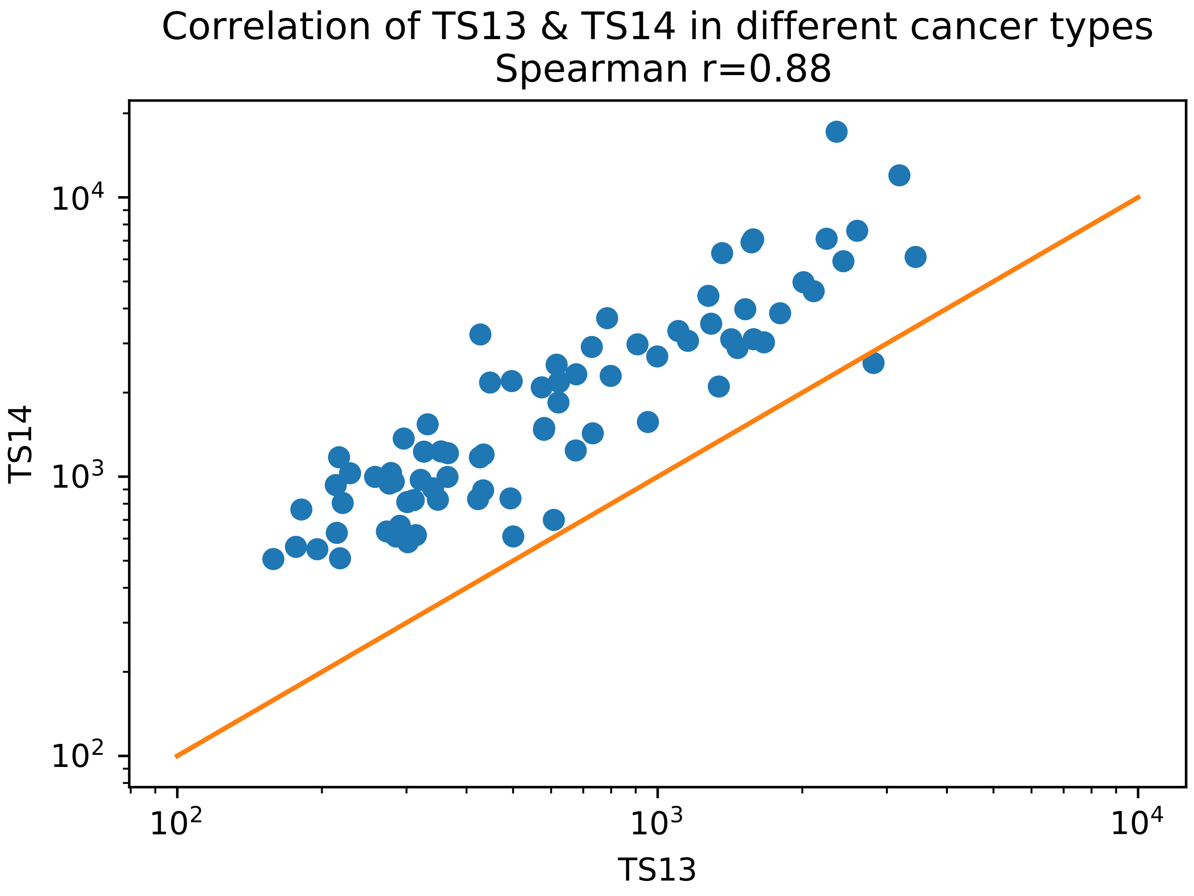

2 Supplementary Note 1: TensorSignature Vignettes

TS01-N[C>T]G (5meC>T)

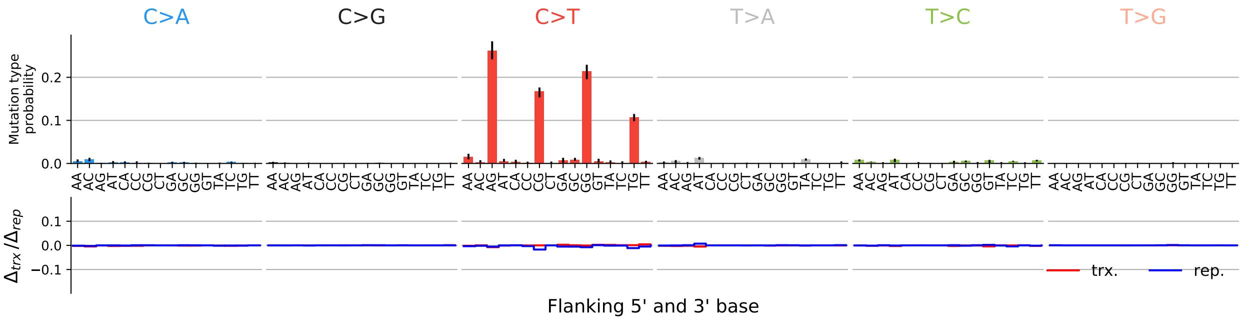

Supplementary Note 1 Fig. 1: **TS01: Single base substitution spectrum.** Bars indicate maximum likelihood estimates and error bars determine 95% bootstrap confidence intervals.

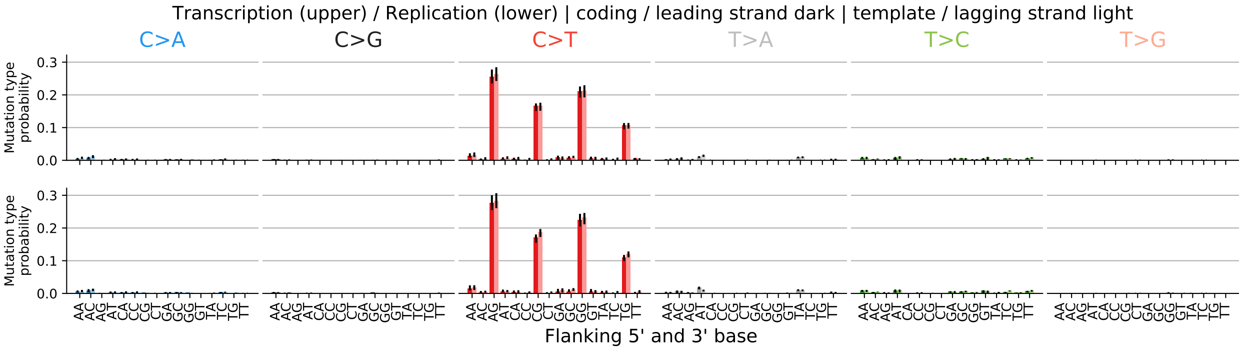

Supplementary Note 1 Fig. 2: **TS01: Single base substitution spectra for template/coding and leading/lagging strand DNA.** Bars indicate maximum likelihood estimates and error bars determine 95% bootstrap confidence intervals.

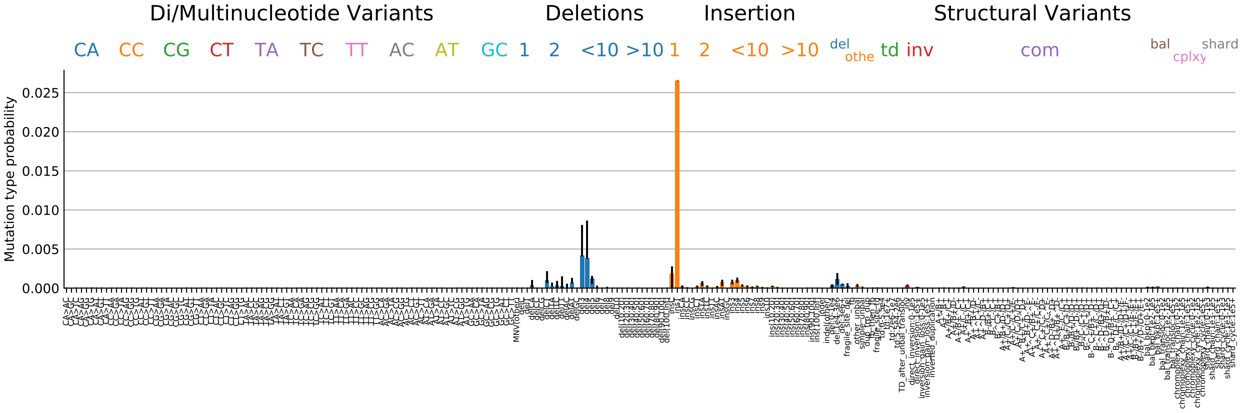

Supplementary Note 1 Fig. 3: **TS01: Spectrum other mutation types.** Bars indicate maximum likelihood estimates and error bars determine 95% bootstrap confidence intervals.

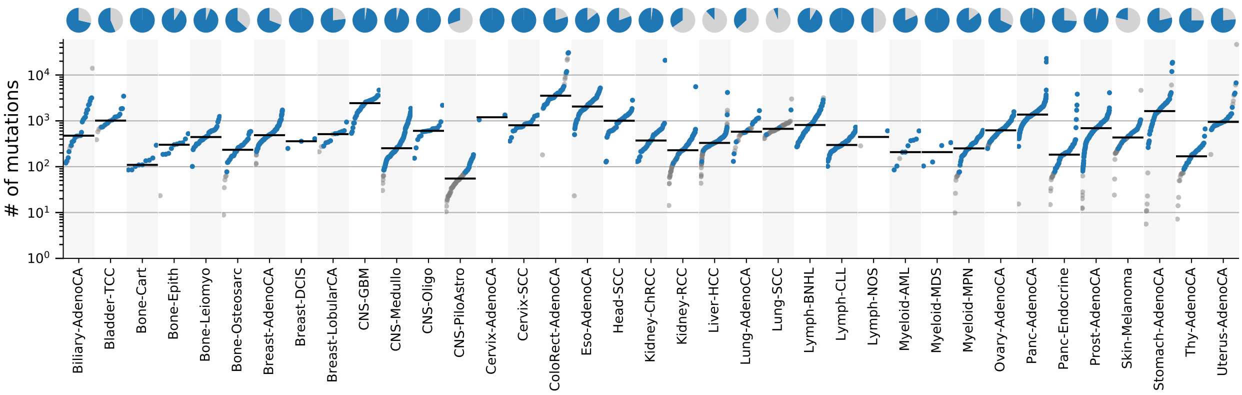

Supplementary Note 1 Fig. 4: **TS01: Signature activity in different cancer types.** Dots indicate maximum likelihood estimates and colored blue if the sample exposure exceeded the typical signature activity. Pie charts indicate the proportion of samples with detectable signature contribution.

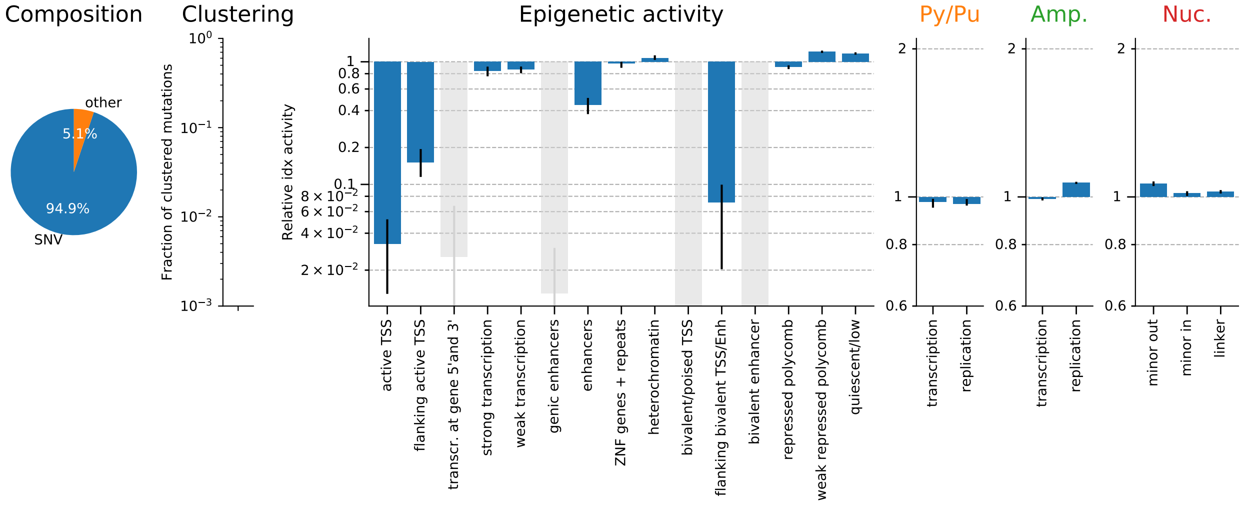

Supplementary Note 1 Fig. 5: **TS01: Signature specific tensor coefficients.** Bars and pie charts indicate maximum likelihood estimates and error bars determine 95% bootstrap confidence intervals.

TS02-N[C>T]N (unknown)

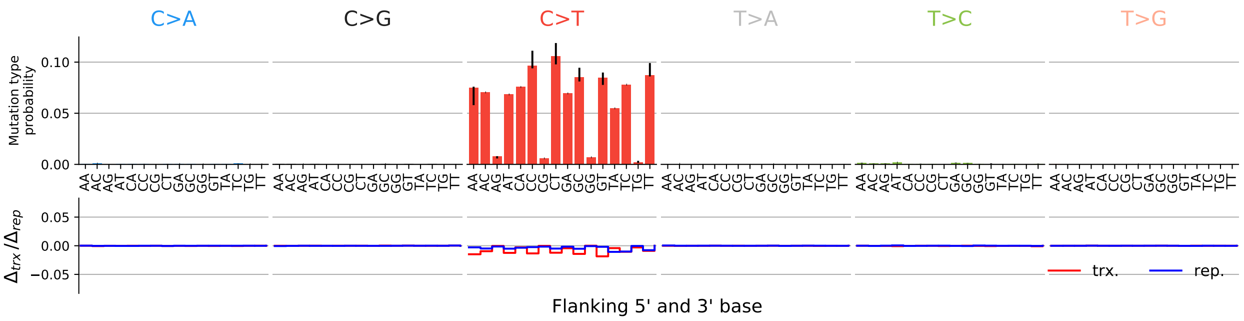

Supplementary Note 1 Fig. 6: **TS02: Single base substitution spectrum.** Bars indicate maximum likelihood estimates and error bars determine 95% bootstrap confidence intervals.

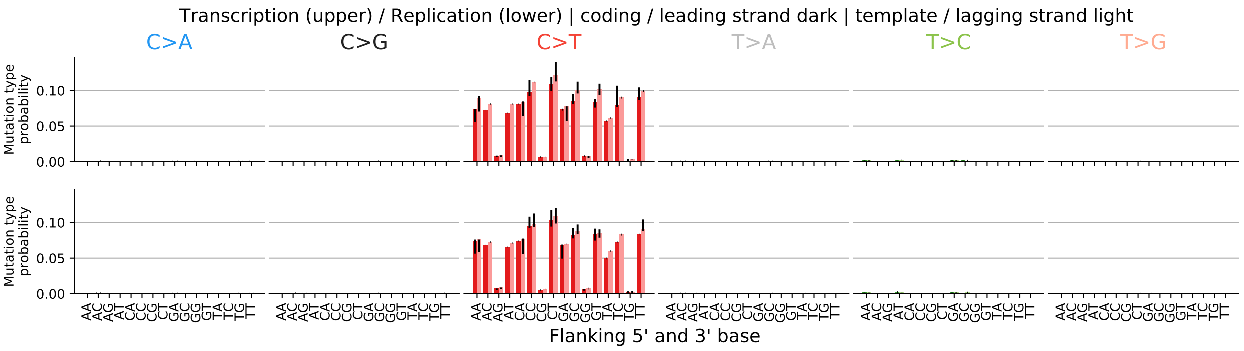

Supplementary Note 1 Fig. 7: **TS02: Single base substitution spectra for template/coding and leading/lagging strand DNA.** Bars indicate maximum likelihood estimates and error bars determine 95% bootstrap confidence intervals.

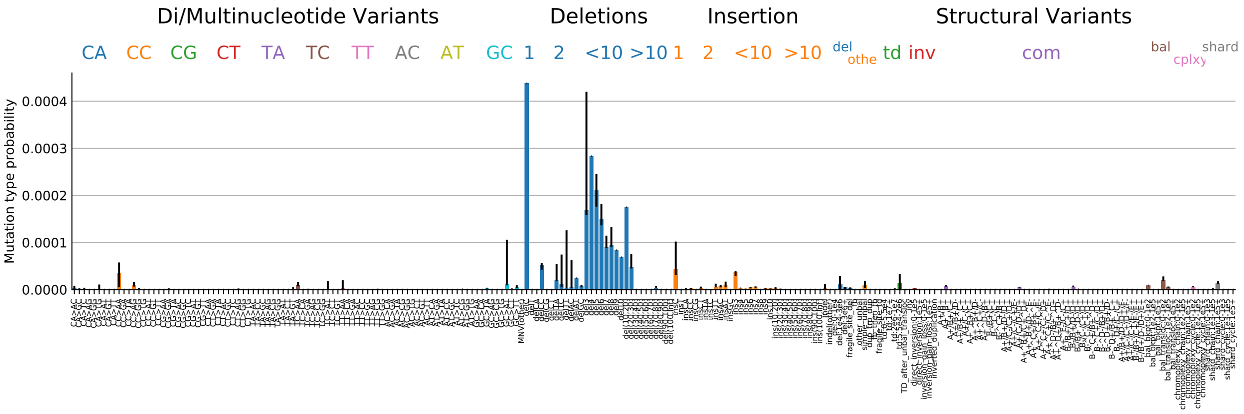

Supplementary Note 1 Fig. 8: **TS02: Spectrum other mutation types.** Bars indicate maximum likelihood estimates and error bars determine 95% bootstrap confidence intervals.

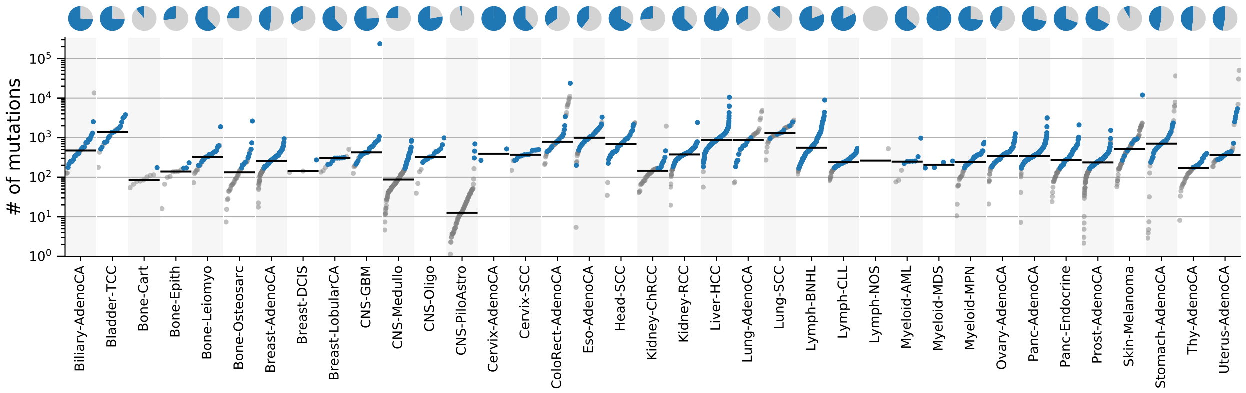

Supplementary Note 1 Fig. 9: **TS02: Signature activity in different cancer types.** Dots indicate maximum likelihood estimates and colored blue if the sample exposure exceeded the typical signature activity. Pie charts indicate the proportion of samples with detectable signature contribution.

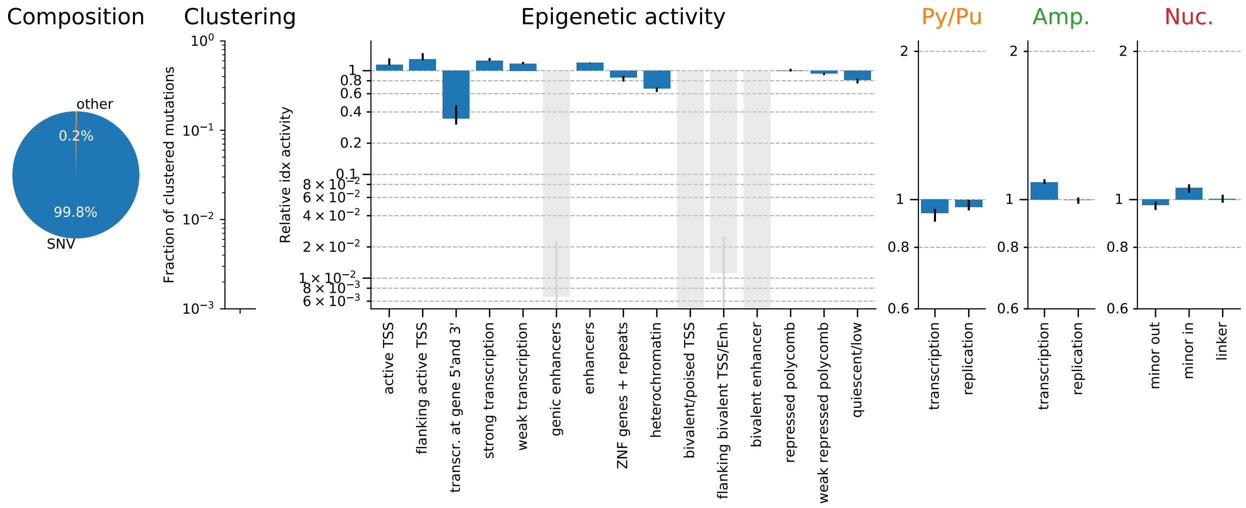

Supplementary Note 1 Fig. 10: **TS02: Signature specific tensor coefficients.** Bars and pie charts indicate maximum likelihood estimates and error bars determine 95% bootstrap confidence intervals.

TS03-N[N>N]N-q (unknown/quiet)

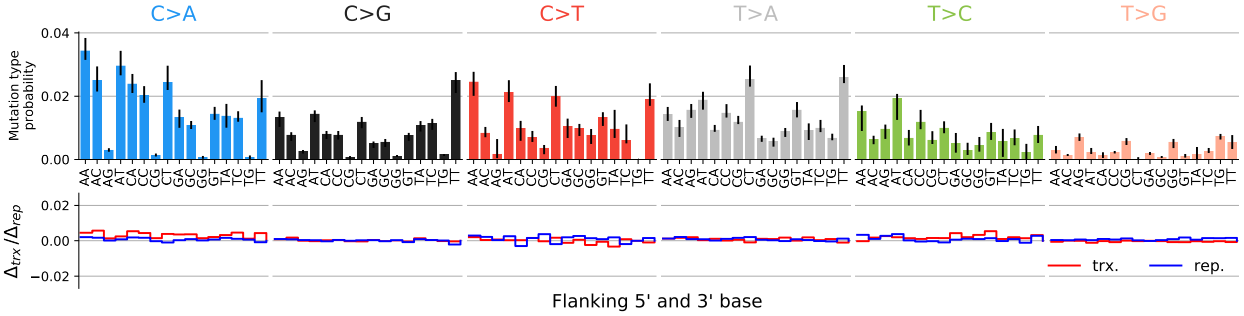

Supplementary Note 1 Fig. 11: **TS03: Single base substitution spectrum.** Bars indicate maximum likelihood estimates and error bars determine 95% bootstrap confidence intervals.

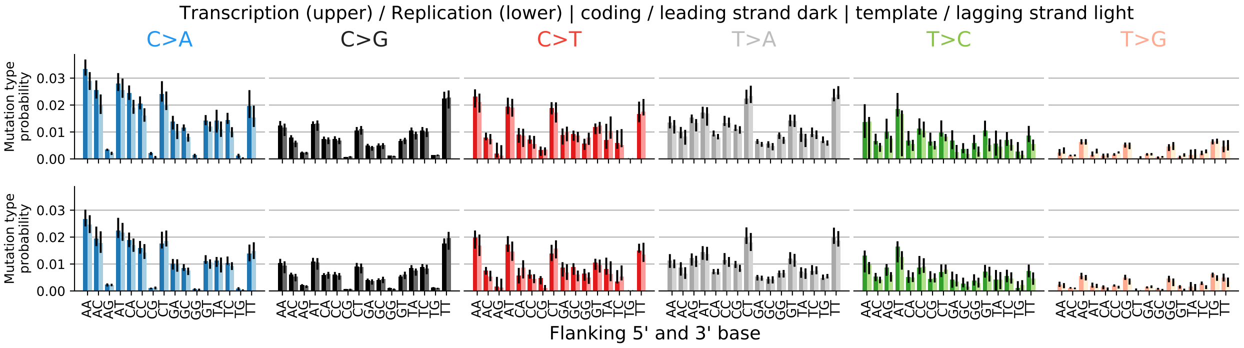

Supplementary Note 1 Fig. 12: **TS03: Single base substitution spectra for template/-coding and leading/lagging strand DNA.** Bars indicate maximum likelihood estimates and error bars determine 95% bootstrap confidence intervals.

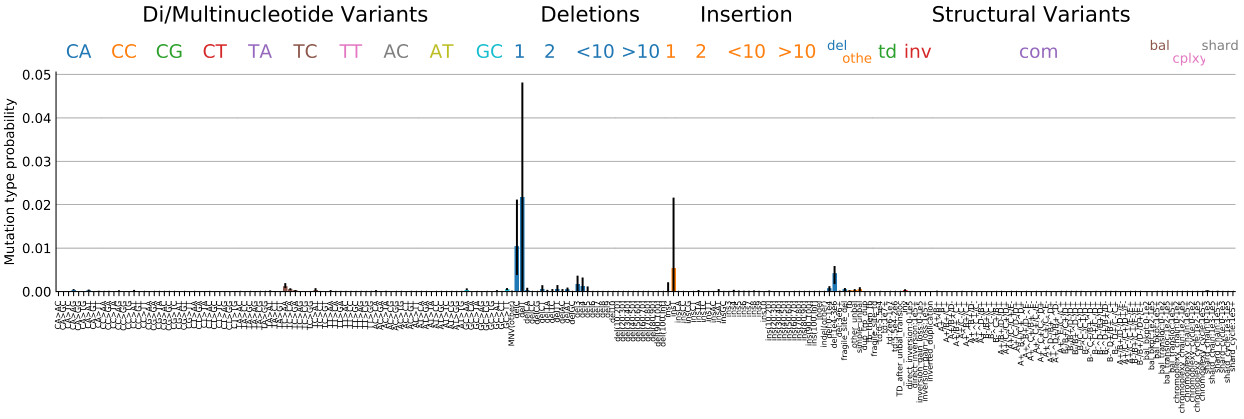

Supplementary Note 1 Fig. 13: **TS03: Spectrum other mutation types.** Bars indicate maximum likelihood estimates and error bars determine 95% bootstrap confidence intervals.

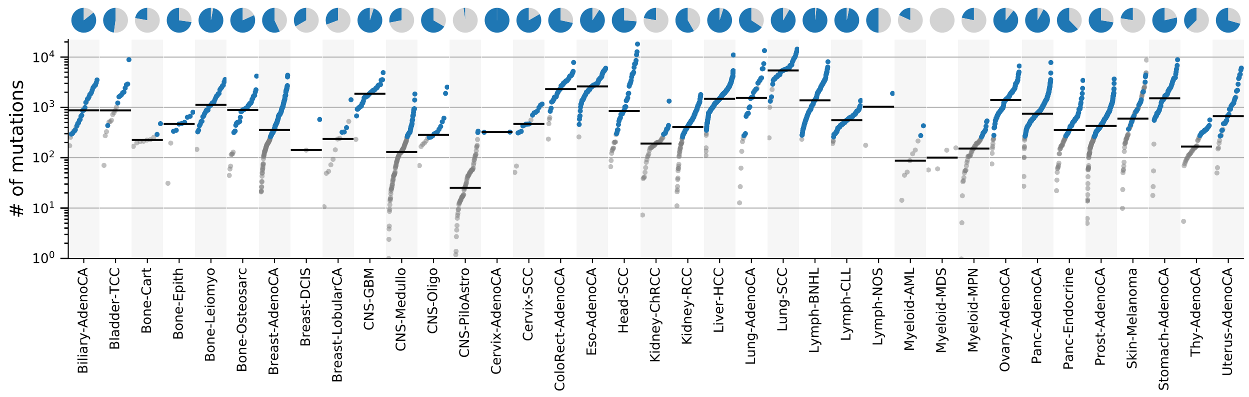

Supplementary Note 1 Fig. 14: **TS03: Signature activity in different cancer types.** Dots indicate maximum likelihood estimates and colored blue if the sample exposure exceeded the typical signature activity. Pie charts indicate the proportion of samples with detectable signature contribution.

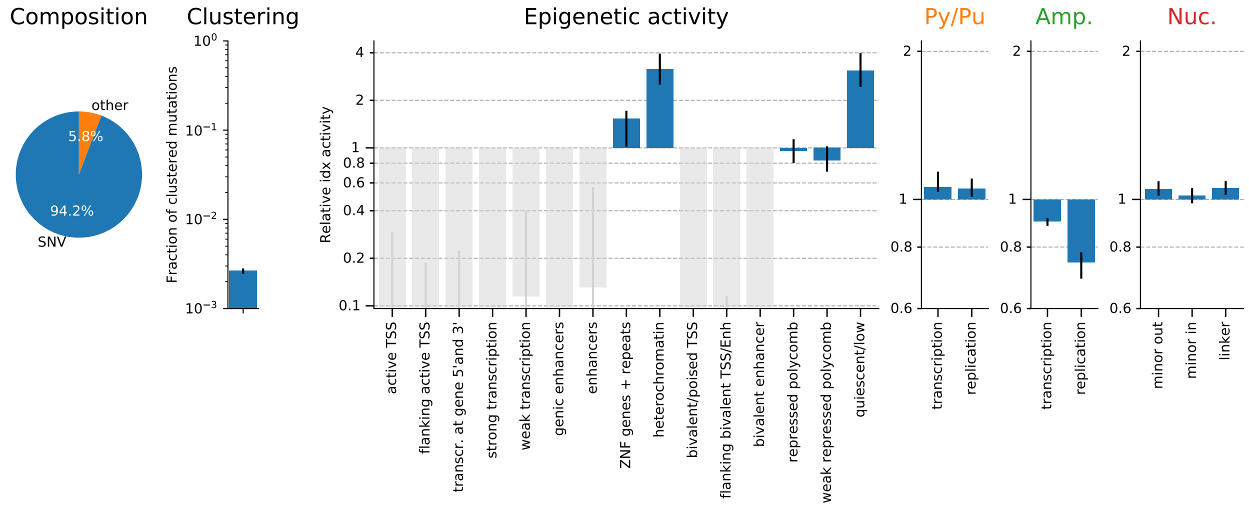

Supplementary Note 1 Fig. 15: **TS03: Signature specific tensor coefficients.** Bars and pie charts indicate maximum likelihood estimates and error bars determine 95% bootstrap confidence intervals.

TS04-N[N>N]N (unknown/active)

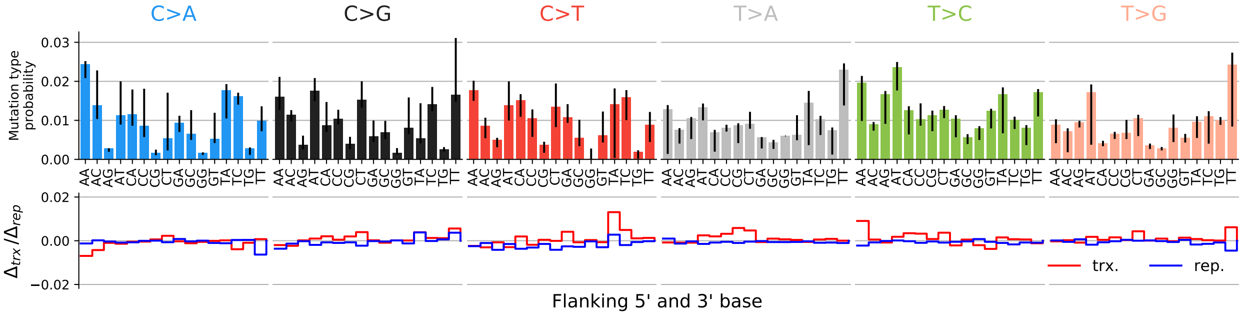

Supplementary Note 1 Fig. 16: **TS04: Single base substitution spectrum.** Bars indicate maximum likelihood estimates and error bars determine 95% bootstrap confidence intervals.

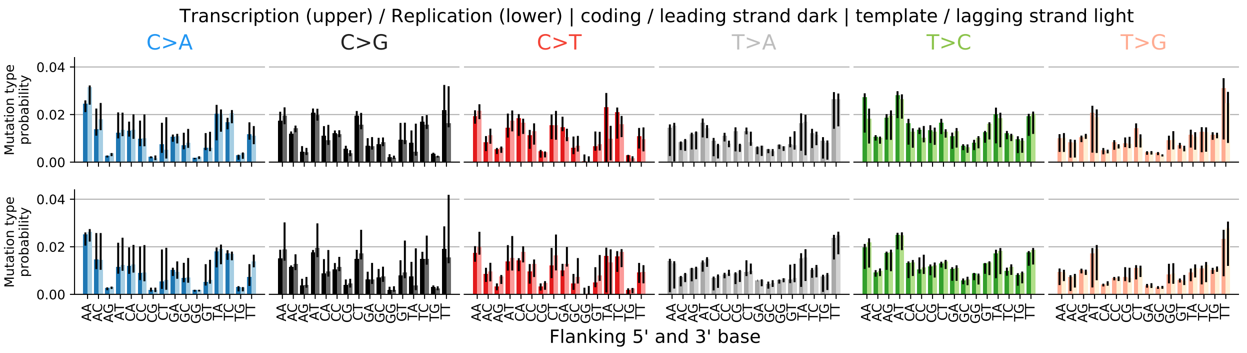

Supplementary Note 1 Fig. 17: **TS04: Single base substitution spectra for template/-coding and leading/lagging strand DNA.** Bars indicate maximum likelihood estimates and error bars determine 95% bootstrap confidence intervals.

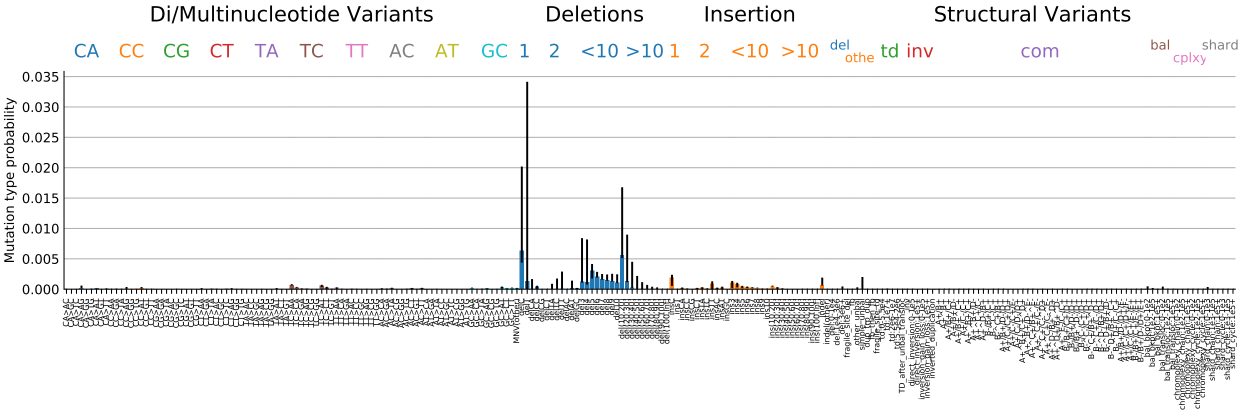

Supplementary Note 1 Fig. 18: **TS04: Spectrum other mutation types.** Bars indicate maximum likelihood estimates and error bars determine 95% bootstrap confidence intervals.

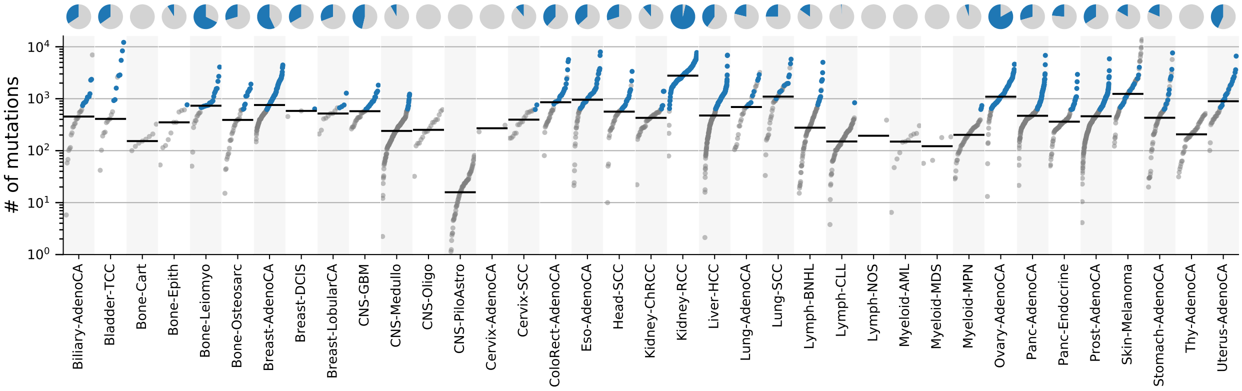

Supplementary Note 1 Fig. 19: **TS04: Signature activity in different cancer types.** Dots indicate maximum likelihood estimates and colored blue if the sample exposure exceeded the typical signature activity. Pie charts indicate the proportion of samples with detectable signature contribution.

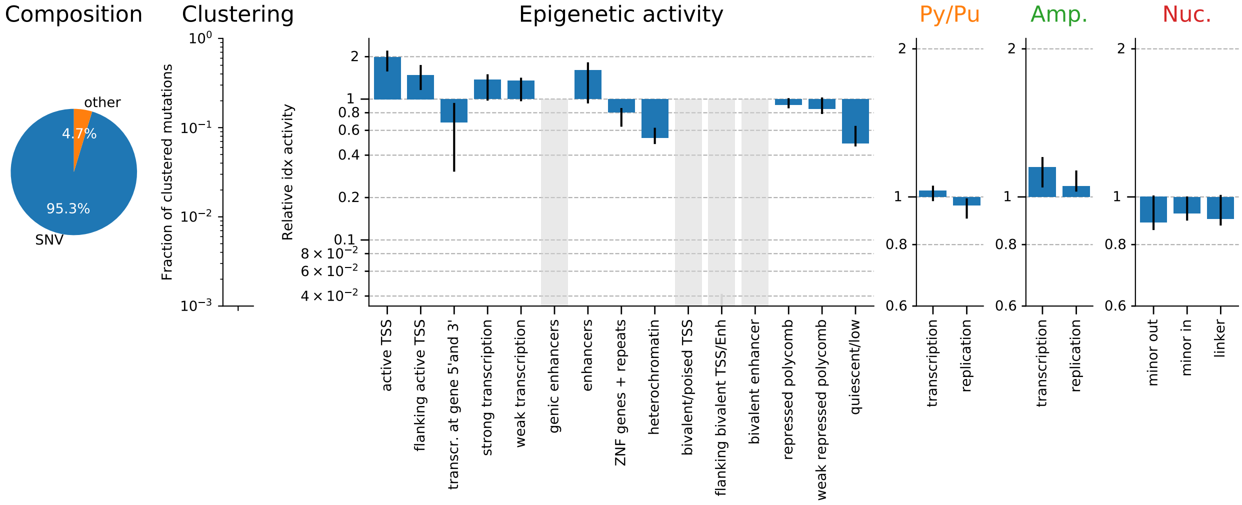

Supplementary Note 1 Fig. 20: **TS04: Signature specific tensor coefficients.** Bars and pie charts indicate maximum likelihood estimates and error bars determine 95% bootstrap confidence intervals.

TS05-T[C>T]N (UV/GG-NER)

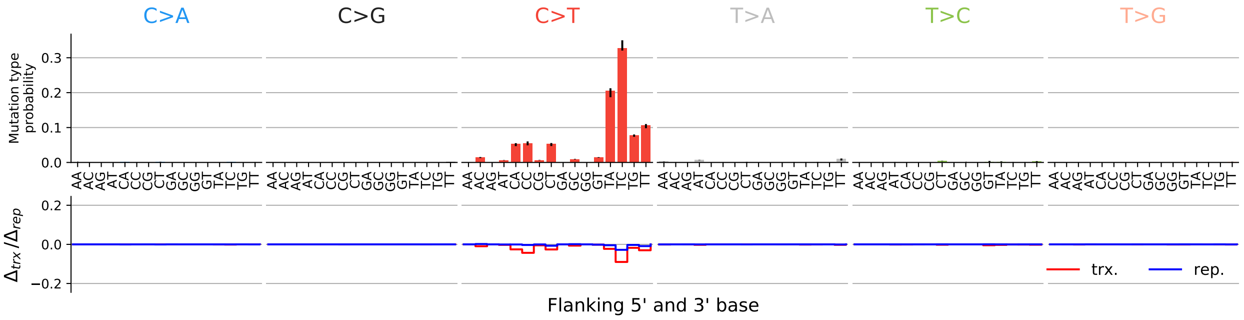

Supplementary Note 1 Fig. 21: **TS05: Single base substitution spectrum.** Bars indicate maximum likelihood estimates and error bars determine 95% bootstrap confidence intervals.

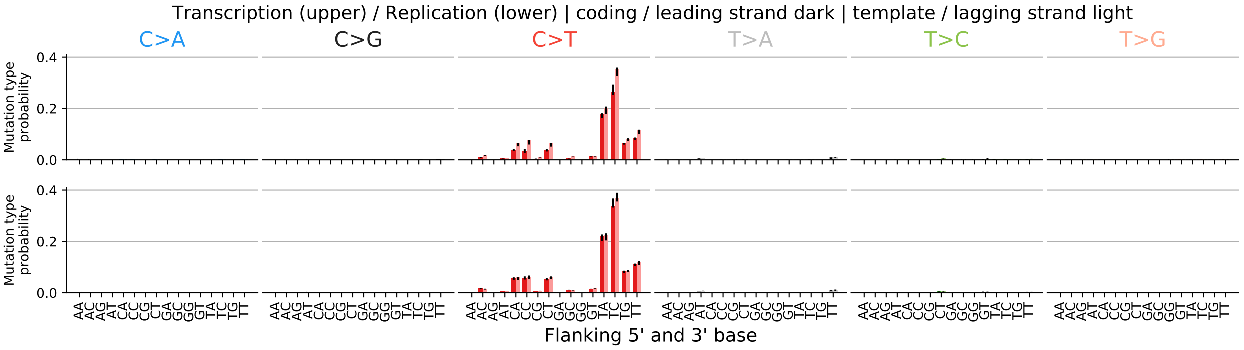

Supplementary Note 1 Fig. 22: **TS05: Single base substitution spectra for template/-coding and leading/lagging strand DNA.** Bars indicate maximum likelihood estimates and error bars determine 95% bootstrap confidence intervals.

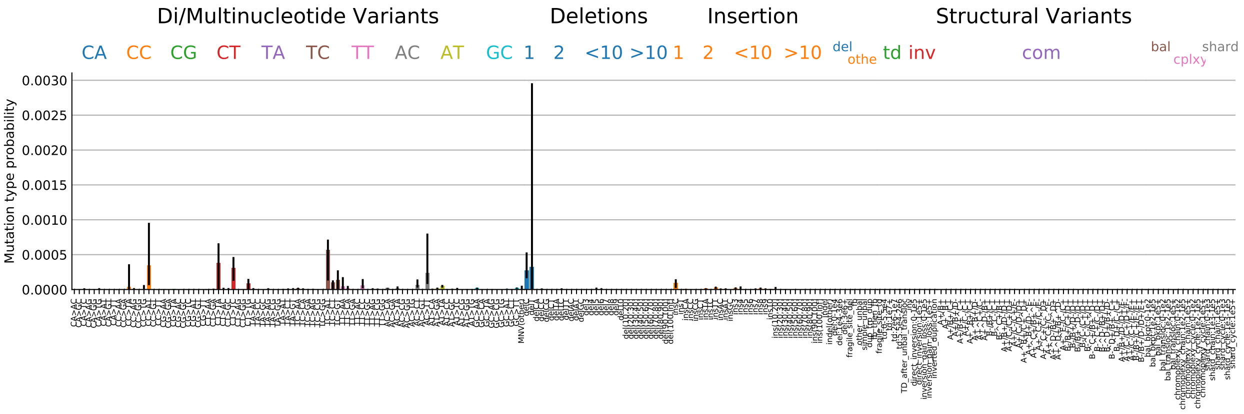

Supplementary Note 1 Fig. 23: **TS05: Spectrum other mutation types.** Bars indicate maximum likelihood estimates and error bars determine 95% bootstrap confidence intervals.

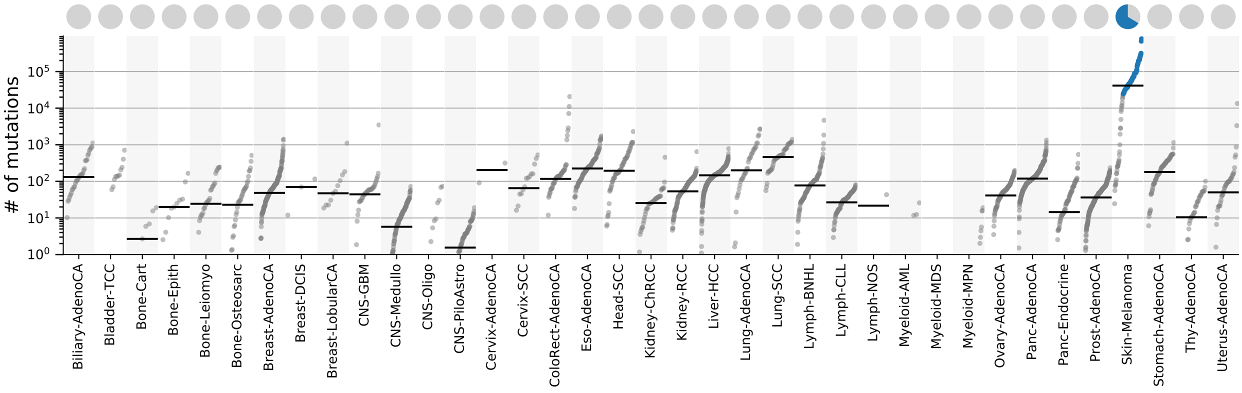

Supplementary Note 1 Fig. 24: **TS05: Signature activity in different cancer types.** Dots indicate maximum likelihood estimates and colored blue if the sample exposure exceeded the typical signature activity. Pie charts indicate the proportion of samples with detectable signature contribution.

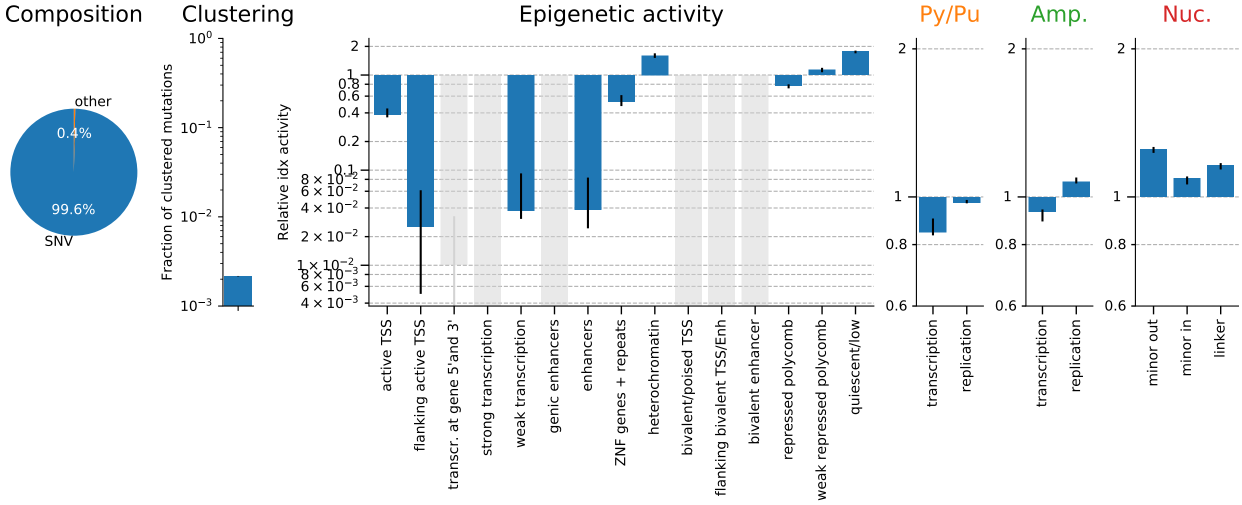

Supplementary Note 1 Fig. 25: **TS05: Signature specific tensor coefficients.** Bars and pie charts indicate maximum likelihood estimates and error bars determine 95% bootstrap confidence intervals.

TS06-Y[C>T]N (UV/GG+TC-NER)

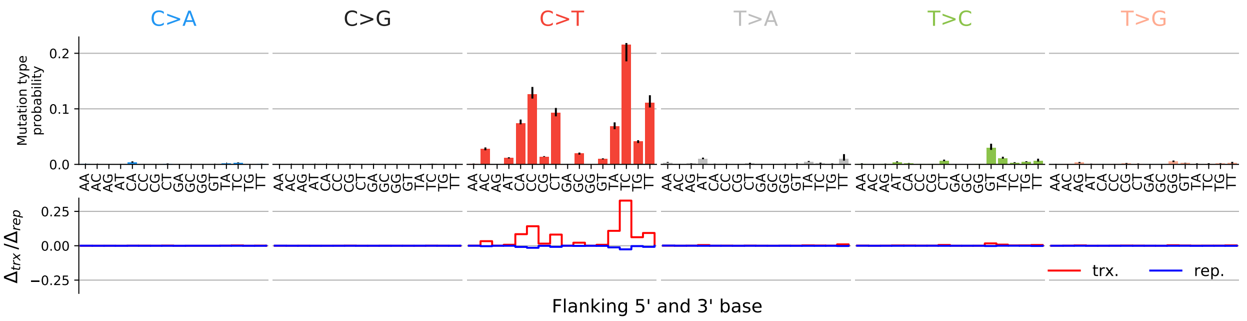

Supplementary Note 1 Fig. 26: **TS06: Single base substitution spectrum.** Bars indicate maximum likelihood estimates and error bars determine 95% bootstrap confidence intervals.

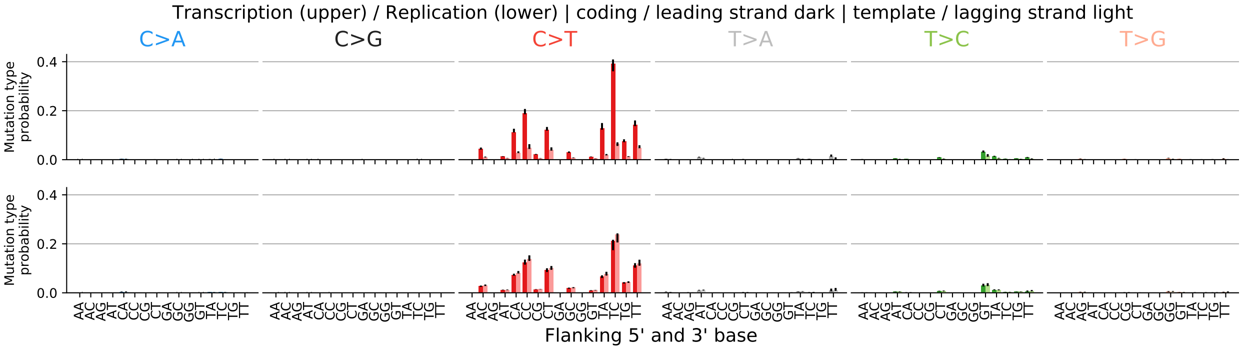

Supplementary Note 1 Fig. 27: **TS06: Single base substitution spectra for template/-coding and leading/lagging strand DNA.** Bars indicate maximum likelihood estimates and error bars determine 95% bootstrap confidence intervals.

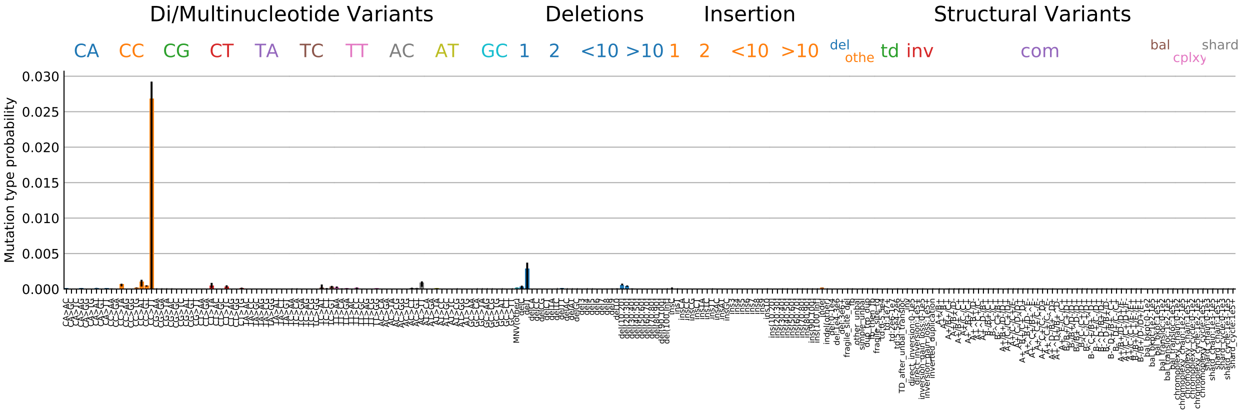

Supplementary Note 1 Fig. 28: **TS06: Spectrum other mutation types.** Bars indicate maximum likelihood estimates and error bars determine 95% bootstrap confidence intervals.

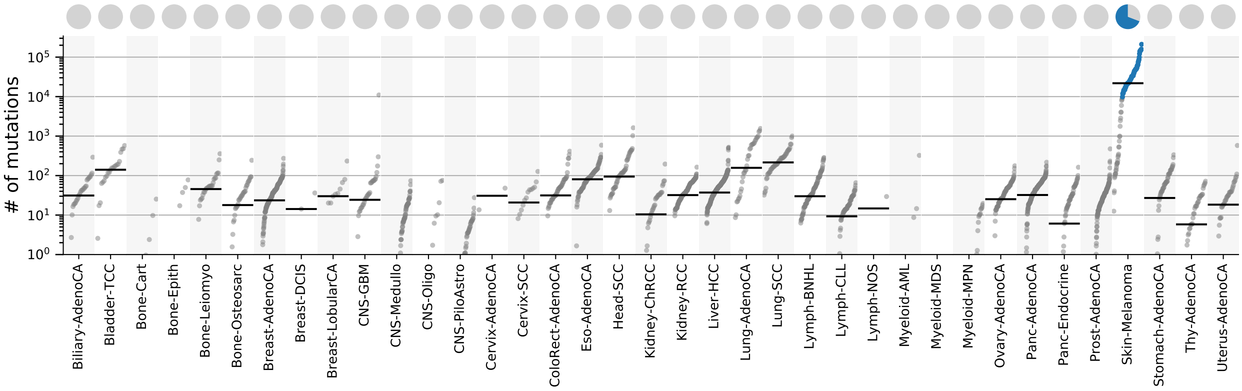

Supplementary Note 1 Fig. 29: **TS06: Signature activity in different cancer types.** Dots indicate maximum likelihood estimates and colored blue if the sample exposure exceeded the typical signature activity. Pie charts indicate the proportion of samples with detectable signature contribution.

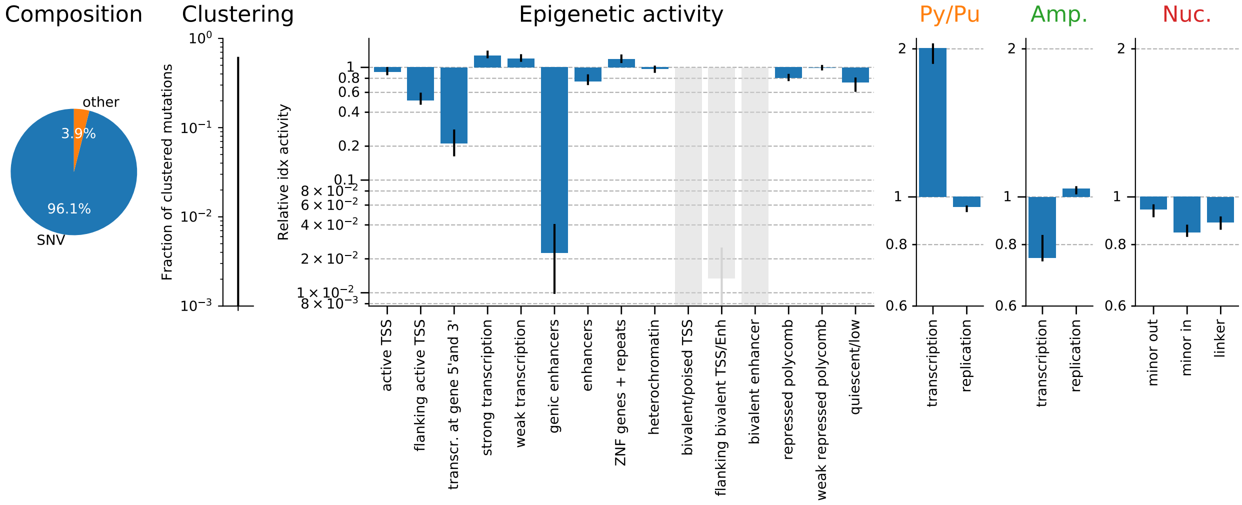

Supplementary Note 1 Fig. 30: **TS06: Signature specific tensor coefficients.** Bars and pie charts indicate maximum likelihood estimates and error bars determine 95% bootstrap confidence intervals.

TS07-N[T>C]N (unknown)

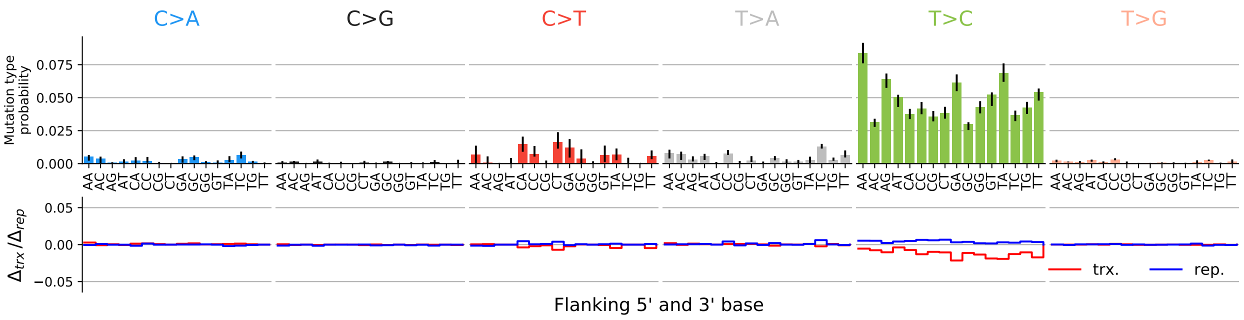

Supplementary Note 1 Fig. 31: **TS07: Single base substitution spectrum.** Bars indicate maximum likelihood estimates and error bars determine 95% bootstrap confidence intervals.

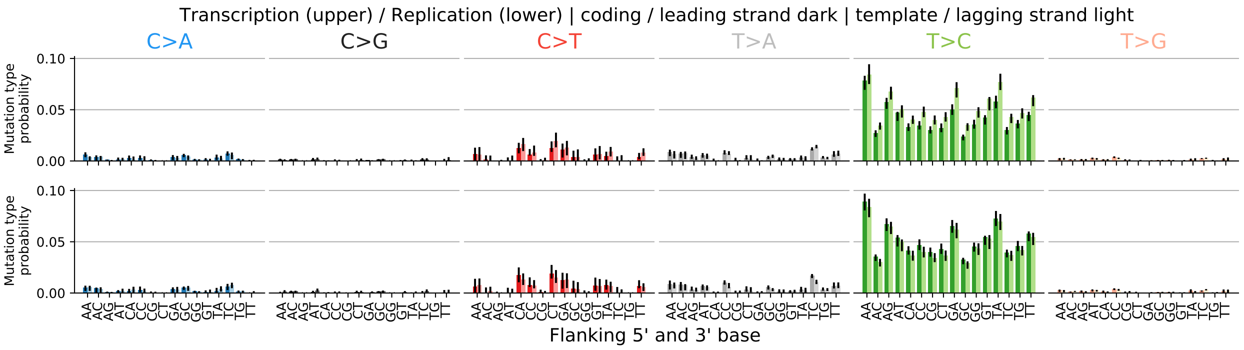

Supplementary Note 1 Fig. 32: **TS07: Single base substitution spectra for template/-coding and leading/lagging strand DNA.** Bars indicate maximum likelihood estimates and error bars determine 95% bootstrap confidence intervals.

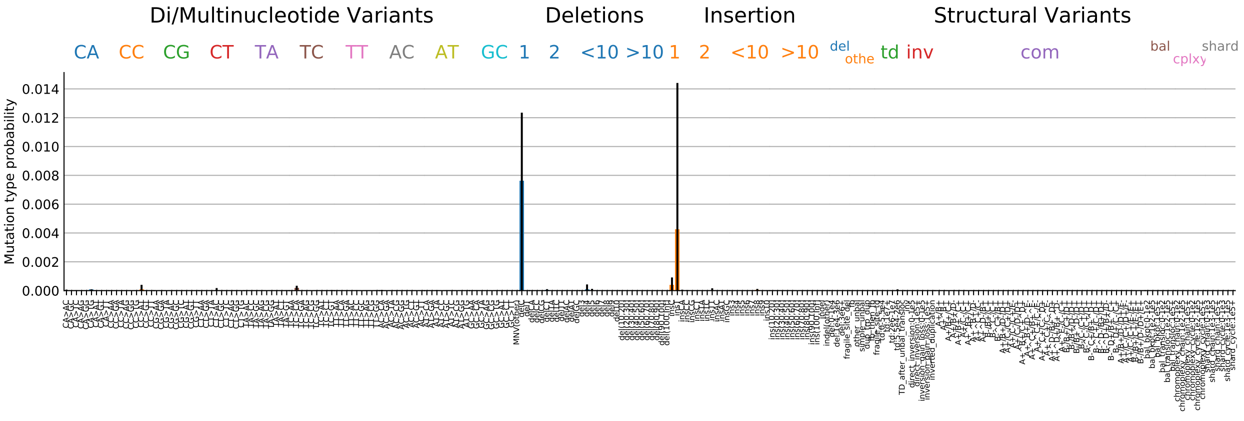

Supplementary Note 1 Fig. 33: **TS07: Spectrum other mutation types.** Bars indicate maximum likelihood estimates and error bars determine 95% bootstrap confidence intervals.

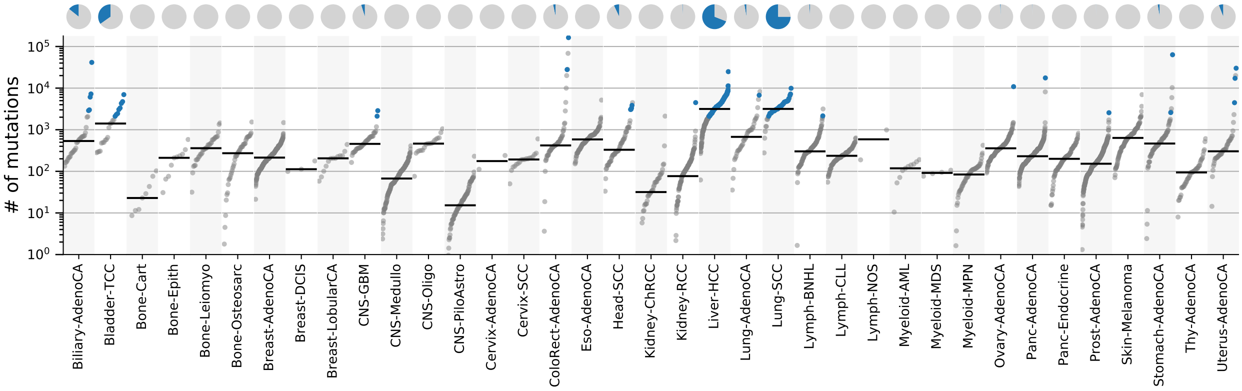

Supplementary Note 1 Fig. 34: **TS07: Signature activity in different cancer types.** Dots indicate maximum likelihood estimates and colored blue if the sample exposure exceeded the typical signature activity. Pie charts indicate the proportion of samples with detectable signature contribution.

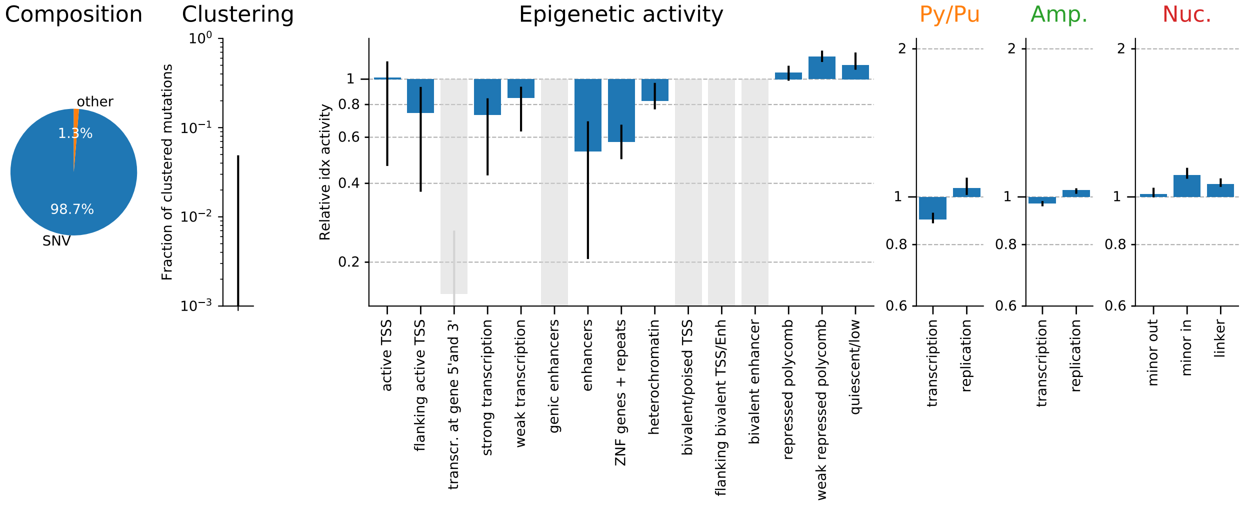

Supplementary Note 1 Fig. 35: **TS07: Signature specific tensor coefficients.** Bars and pie charts indicate maximum likelihood estimates and error bars determine 95% bootstrap confidence intervals.

TS08-A[T>C]W (unknown/TAM)

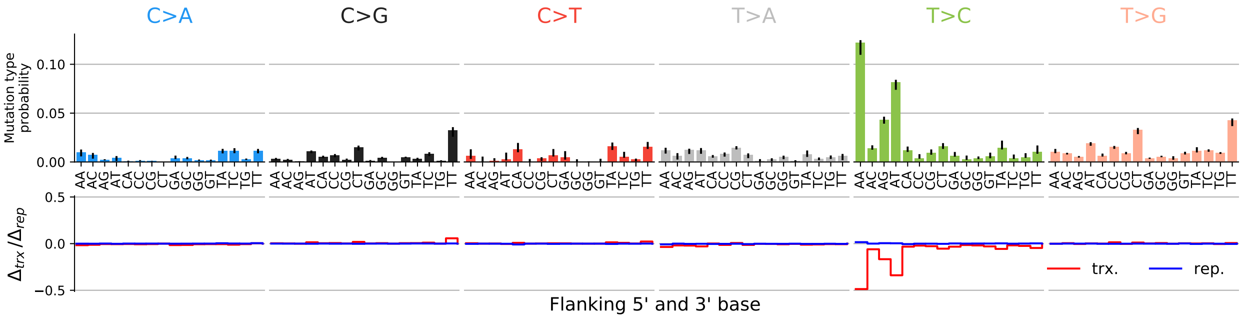

Supplementary Note 1 Fig. 36: **TS08: Single base substitution spectrum.** Bars indicate maximum likelihood estimates and error bars determine 95% bootstrap confidence intervals.

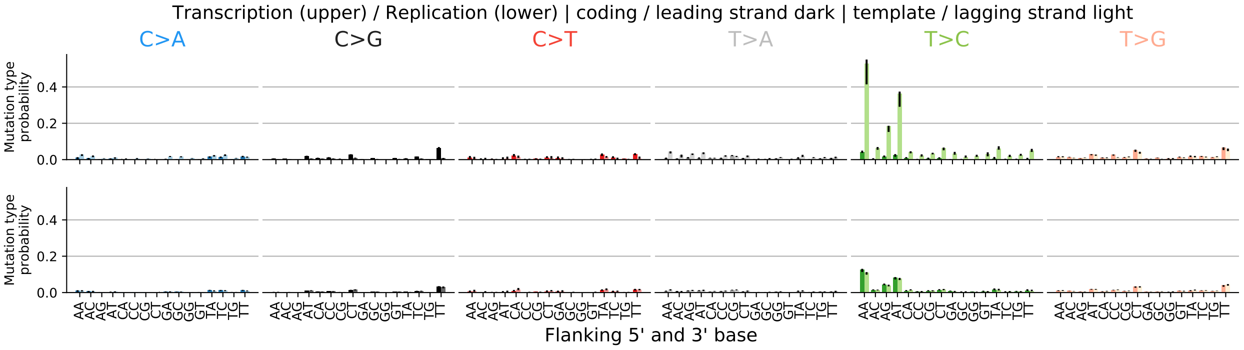

Supplementary Note 1 Fig. 37: **TS08: Single base substitution spectra for template/-coding and leading/lagging strand DNA.** Bars indicate maximum likelihood estimates and error bars determine 95% bootstrap confidence intervals.

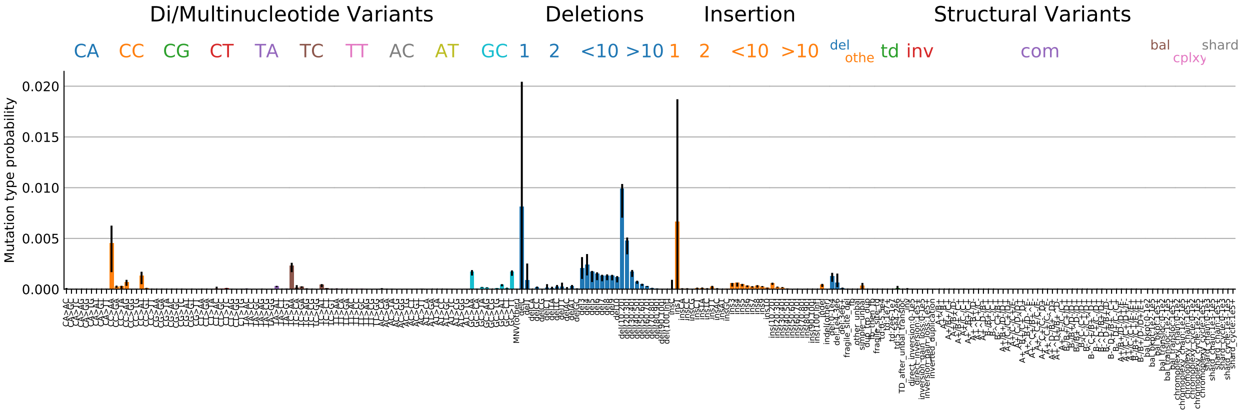

Supplementary Note 1 Fig. 38: **TS08: Spectrum other mutation types.** Bars indicate maximum likelihood estimates and error bars determine 95% bootstrap confidence intervals.

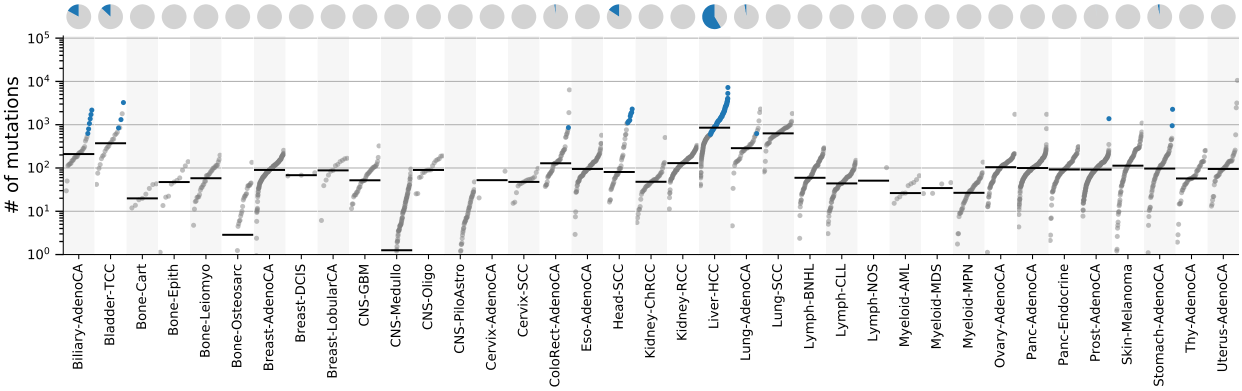

Supplementary Note 1 Fig. 39: **TS08: Signature activity in different cancer types.** Dots indicate maximum likelihood estimates and colored blue if the sample exposure exceeded the typical signature activity. Pie charts indicate the proportion of samples with detectable signature contribution.

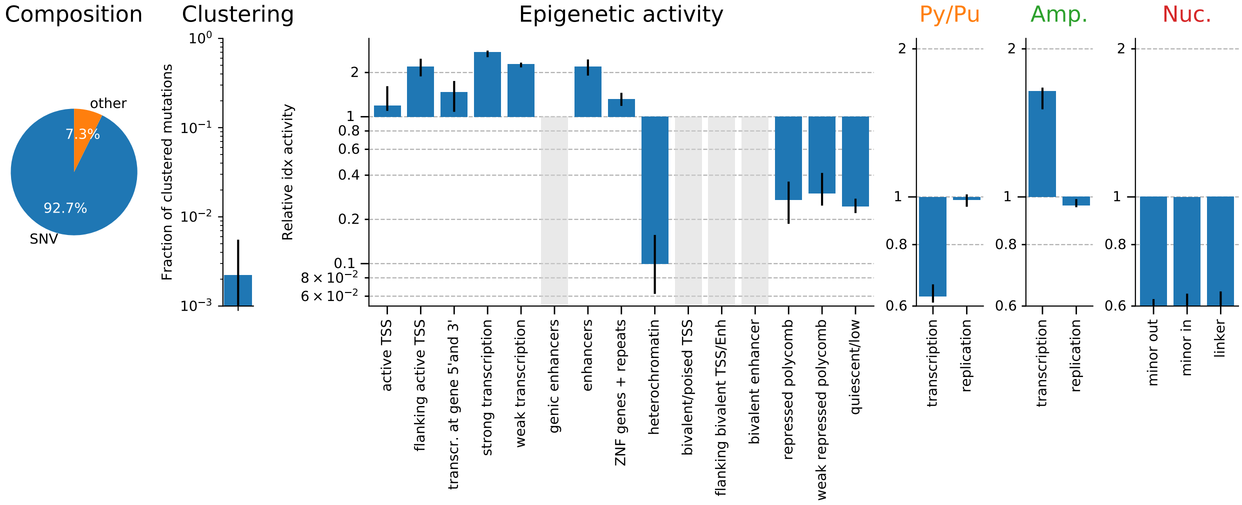

Supplementary Note 1 Fig. 40: **TS08: Signature specific tensor coefficients.** Bars and pie charts indicate maximum likelihood estimates and error bars determine 95% bootstrap confidence intervals.

TS09-N[T>A]N (PAH/AA)

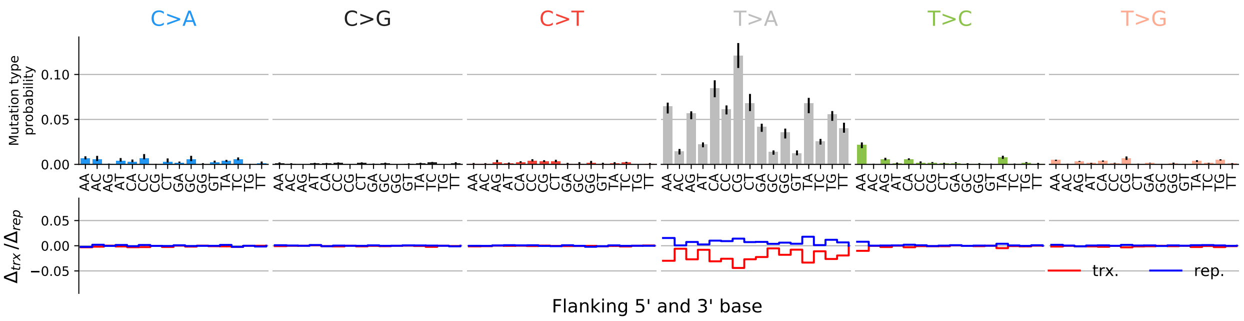

Supplementary Note 1 Fig. 41: **TS09: Single base substitution spectrum.** Bars indicate maximum likelihood estimates and error bars determine 95% bootstrap confidence intervals.

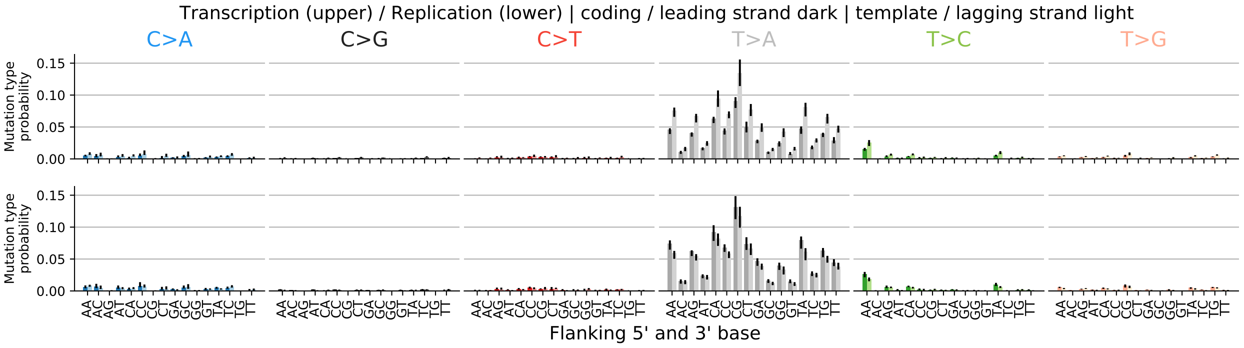

Supplementary Note 1 Fig. 42: **TS09: Single base substitution spectra for template/-coding and leading/lagging strand DNA.** Bars indicate maximum likelihood estimates and error bars determine 95% bootstrap confidence intervals.

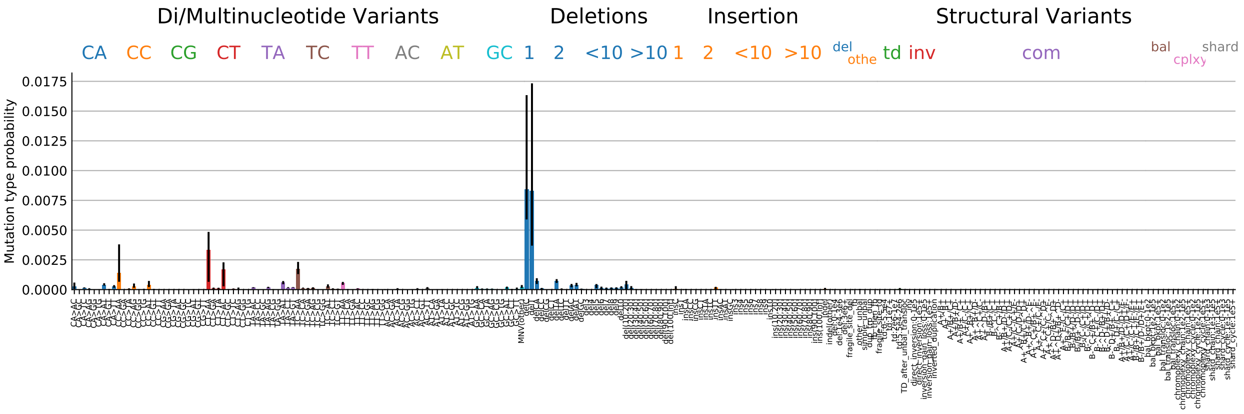

Supplementary Note 1 Fig. 43: **TS09: Spectrum other mutation types.** Bars indicate maximum likelihood estimates and error bars determine 95% bootstrap confidence intervals.

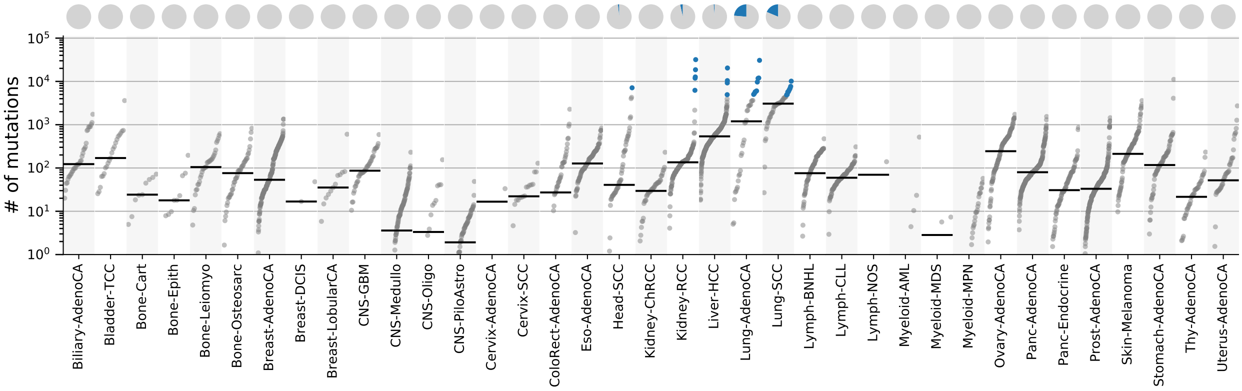

Supplementary Note 1 Fig. 44: **TS09: Signature activity in different cancer types.** Dots indicate maximum likelihood estimates and colored blue if the sample exposure exceeded the typical signature activity. Pie charts indicate the proportion of samples with detectable signature contribution.

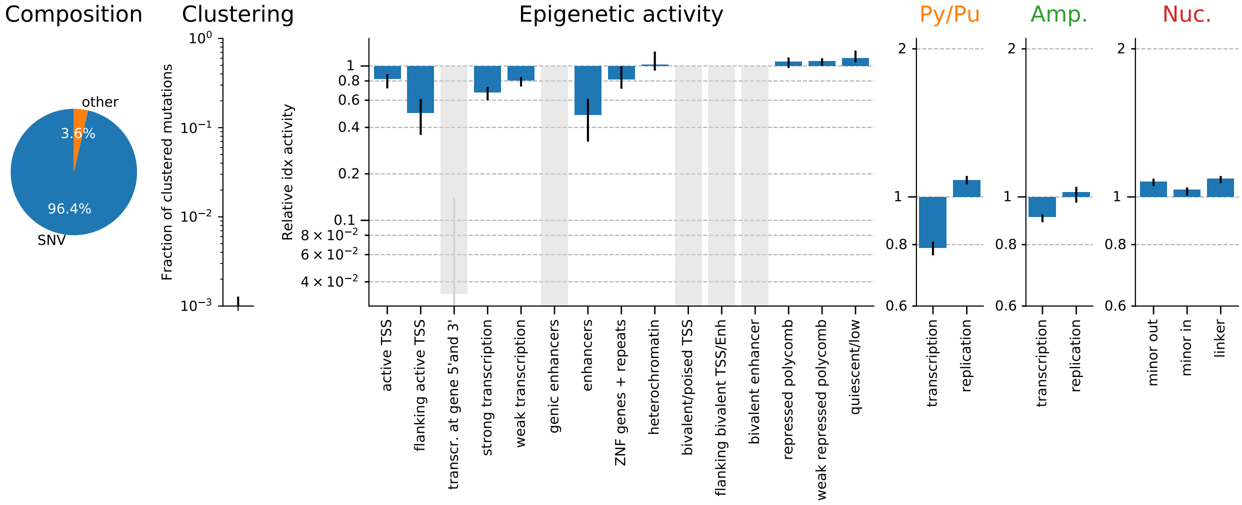

Supplementary Note 1 Fig. 45: **TS09: Signature specific tensor coefficients.** Bars and pie charts indicate maximum likelihood estimates and error bars determine 95% bootstrap confidence intervals.

TS10-N[C>A]N (PAH/B[a]P)

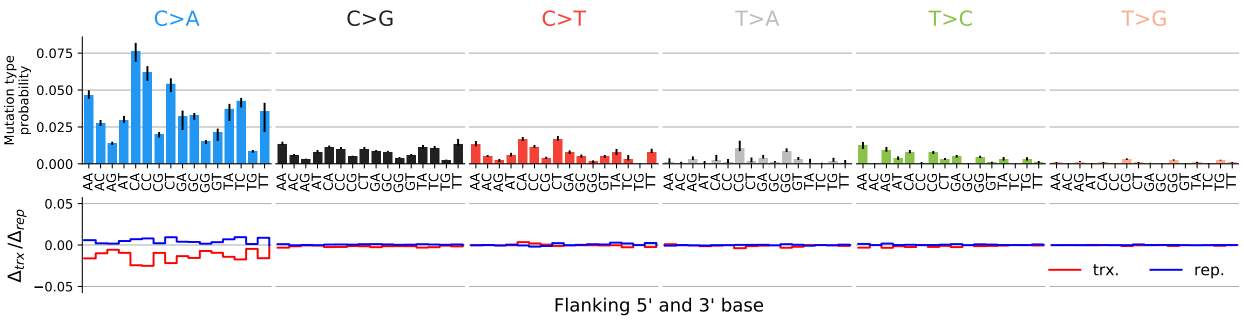

Supplementary Note 1 Fig. 46: **TS10: Single base substitution spectrum.** Bars indicate maximum likelihood estimates and error bars determine 95% bootstrap confidence intervals.

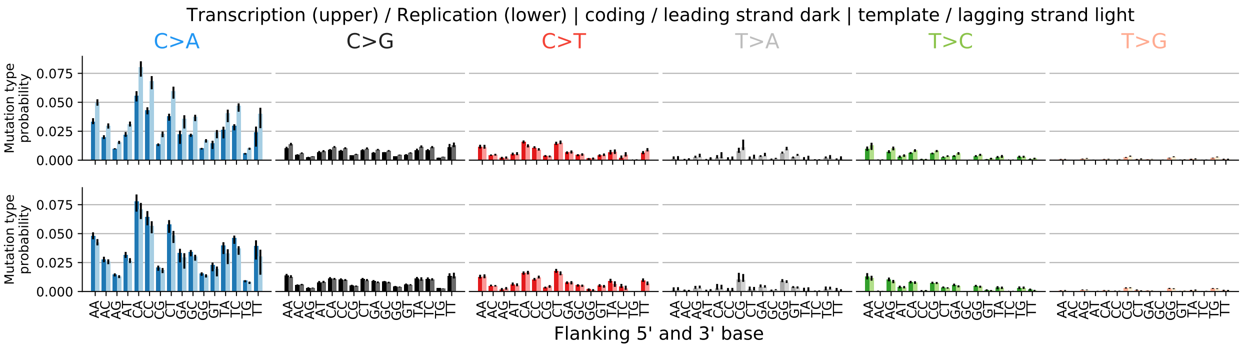

Supplementary Note 1 Fig. 47: **TS10: Single base substitution spectra for template/-coding and leading/lagging strand DNA.** Bars indicate maximum likelihood estimates and error bars determine 95% bootstrap confidence intervals.

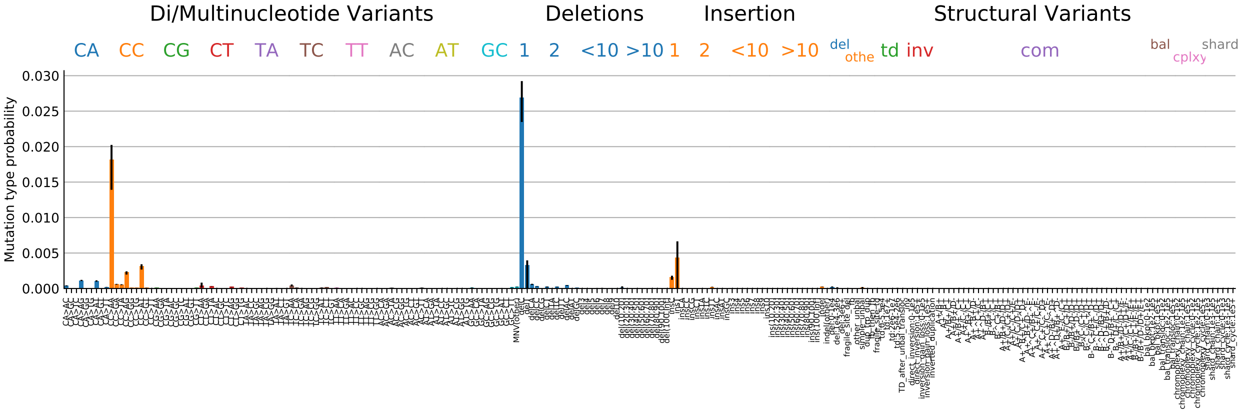

Supplementary Note 1 Fig. 48: **TS10: Spectrum other mutation types.** Bars indicate maximum likelihood estimates and error bars determine 95% bootstrap confidence intervals.

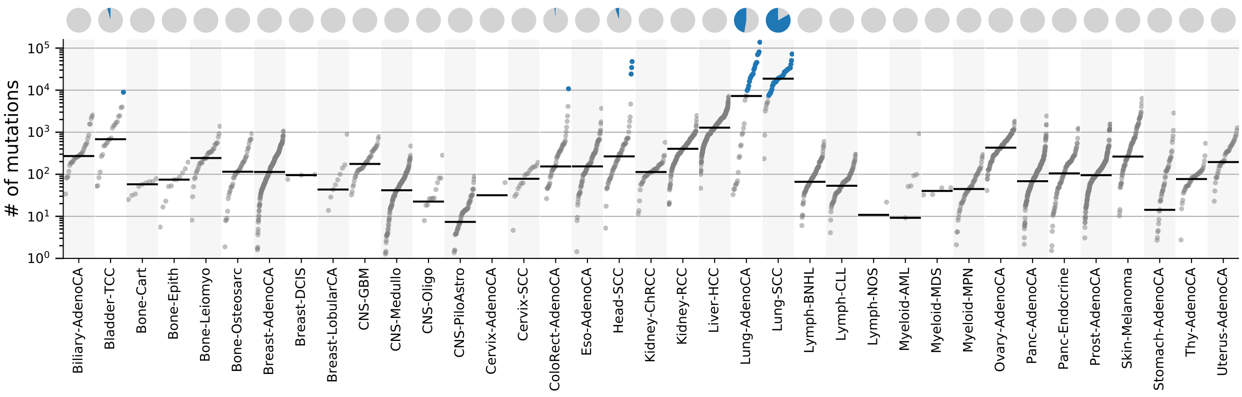

Supplementary Note 1 Fig. 49: **TS10: Signature activity in different cancer types.** Dots indicate maximum likelihood estimates and colored blue if the sample exposure exceeded the typical signature activity. Pie charts indicate the proportion of samples with detectable signature contribution.

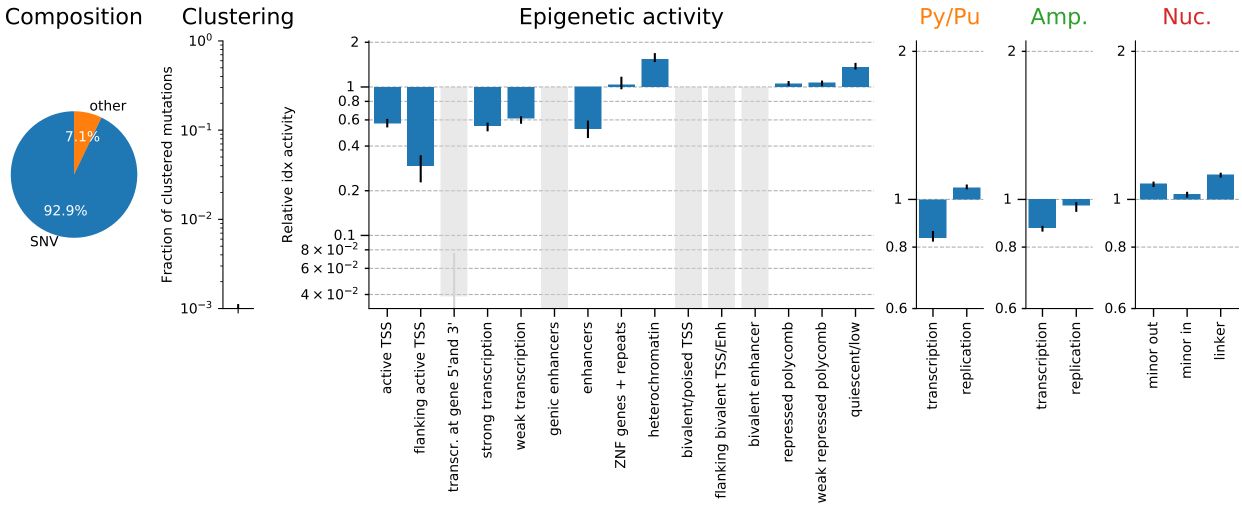

Supplementary Note 1 Fig. 50: **TS10: Signature specific tensor coefficients.** Bars and pie charts indicate maximum likelihood estimates and error bars determine 95% bootstrap confidence intervals.

TS11-T[C>D]W;SV (APOBEC)

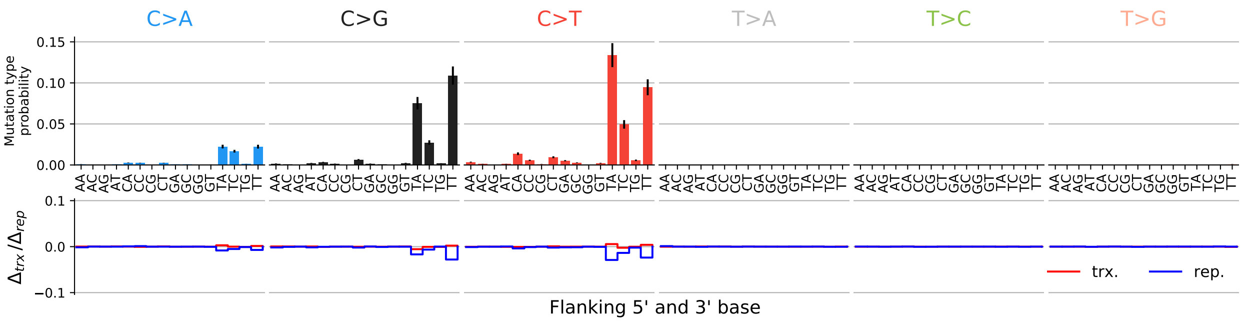

Supplementary Note 1 Fig. 51: **TS11: Single base substitution spectrum.** Bars indicate maximum likelihood estimates and error bars determine 95% bootstrap confidence intervals.

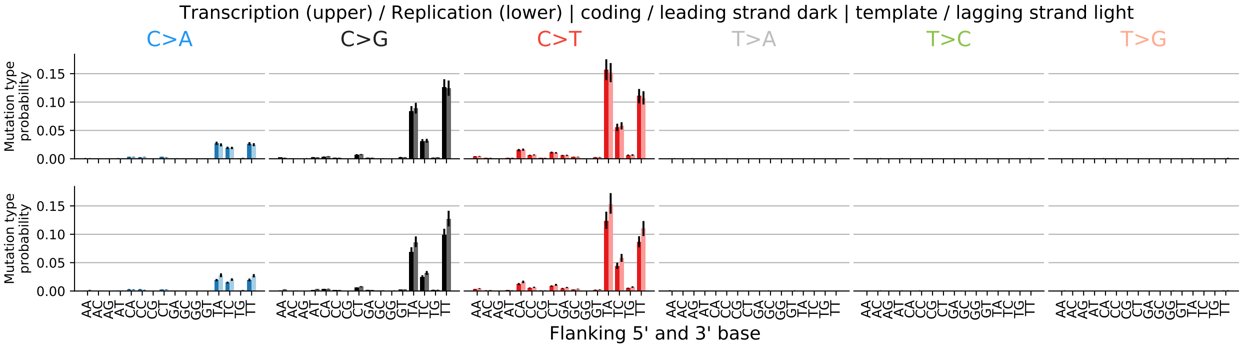

Supplementary Note 1 Fig. 52: **TS11: Single base substitution spectra for template/-coding and leading/lagging strand DNA.** Bars indicate maximum likelihood estimates and error bars determine 95% bootstrap confidence intervals.

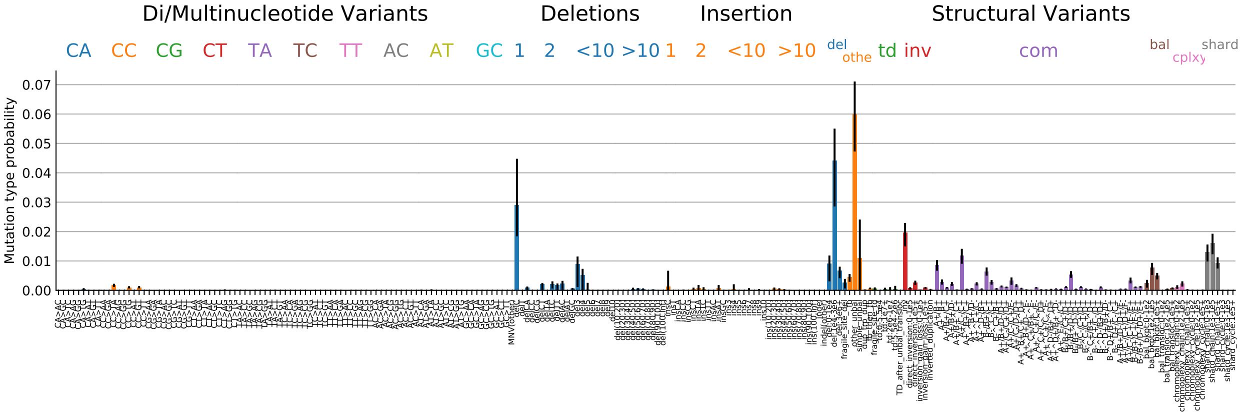

Supplementary Note 1 Fig. 53: **TS11: Spectrum other mutation types.** Bars indicate maximum likelihood estimates and error bars determine 95% bootstrap confidence intervals.

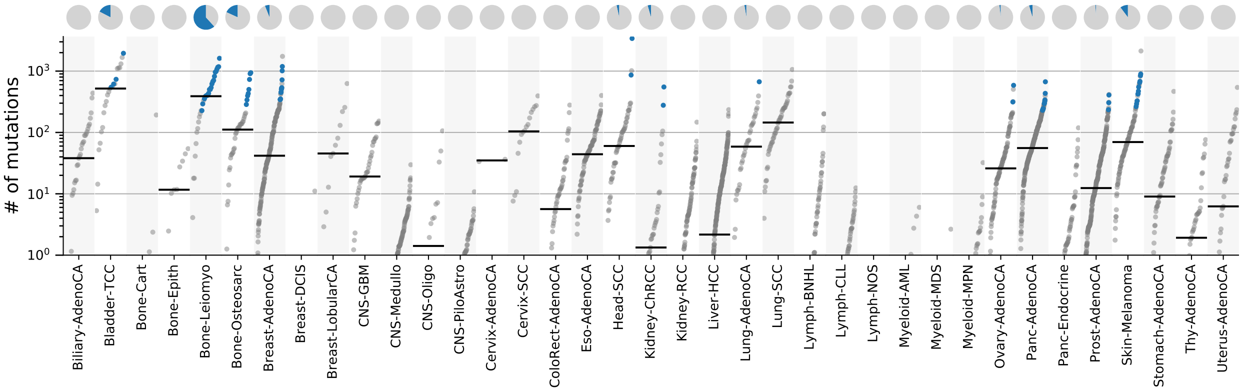

Supplementary Note 1 Fig. 54: **TS11: Signature activity in different cancer types.** Dots indicate maximum likelihood estimates and colored blue if the sample exposure exceeded the typical signature activity. Pie charts indicate the proportion of samples with detectable signature contribution.

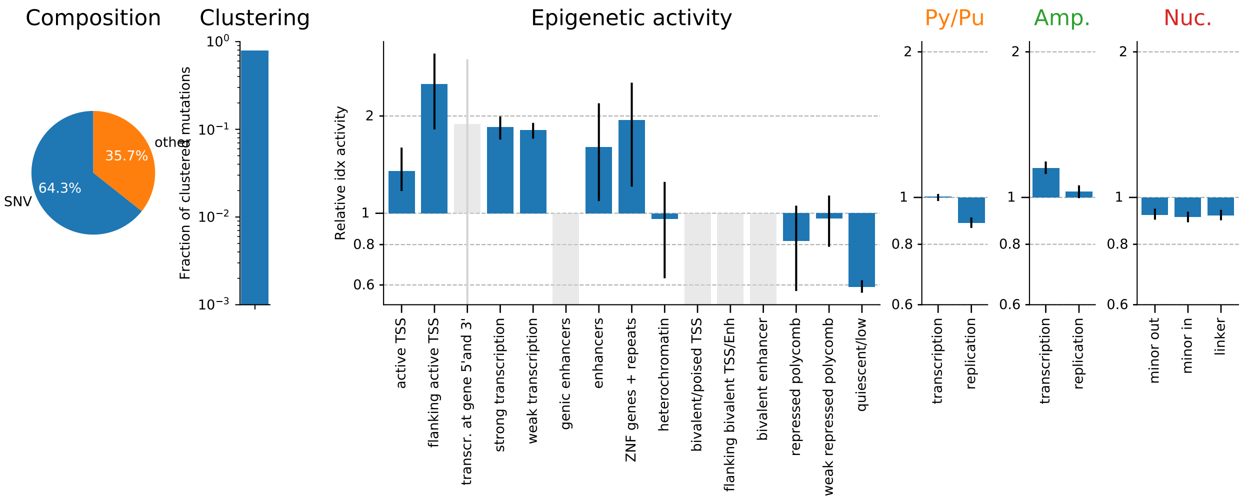

Supplementary Note 1 Fig. 55: **TS11: Signature specific tensor coefficients.** Bars and pie charts indicate maximum likelihood estimates and error bars determine 95% bootstrap confidence intervals.

TS12-T[C>D]W (APOBEC)

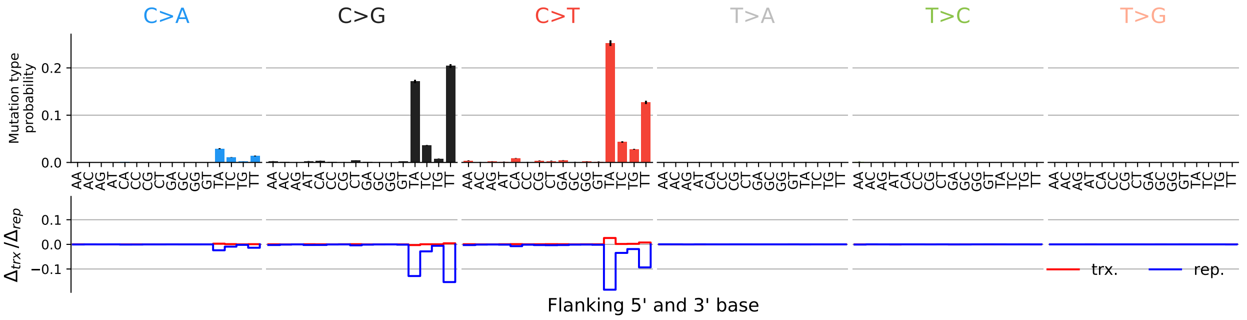

Supplementary Note 1 Fig. 56: **TS12: Single base substitution spectrum.** Bars indicate maximum likelihood estimates and error bars determine 95% bootstrap confidence intervals.

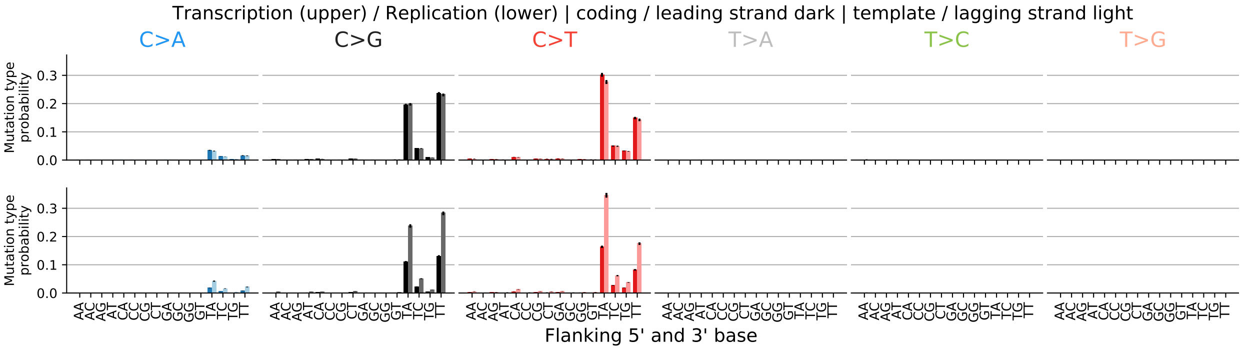

Supplementary Note 1 Fig. 57: **TS12: Single base substitution spectra for template/-coding and leading/lagging strand DNA.** Bars indicate maximum likelihood estimates and error bars determine 95% bootstrap confidence intervals.

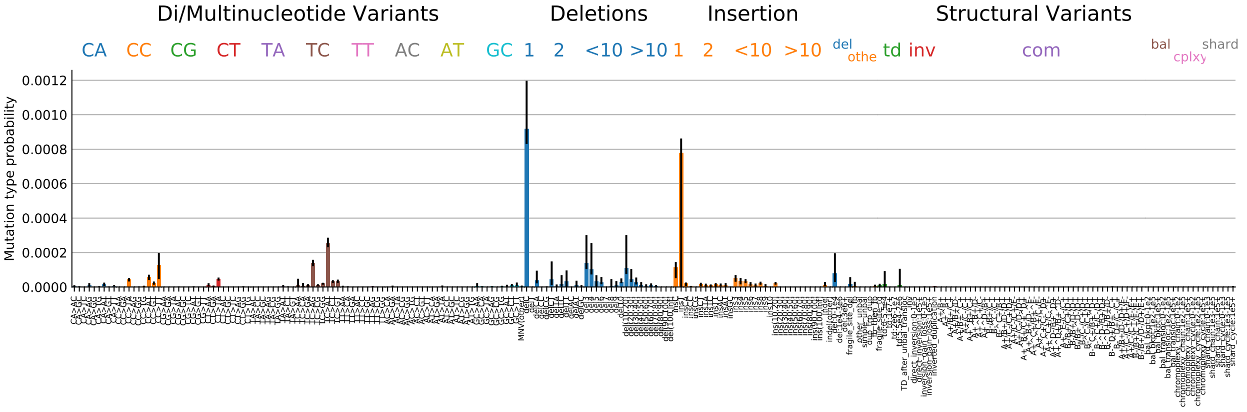

Supplementary Note 1 Fig. 58: **TS12: Spectrum other mutation types.** Bars indicate maximum likelihood estimates and error bars determine 95% bootstrap confidence intervals.

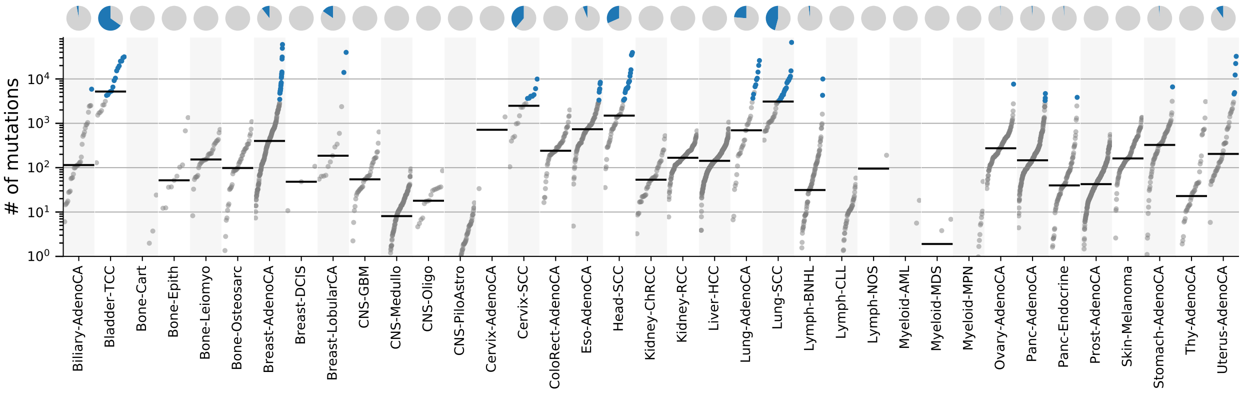

Supplementary Note 1 Fig. 59: **TS12: Signature activity in different cancer types.** Dots indicate maximum likelihood estimates and colored blue if the sample exposure exceeded the typical signature activity. Pie charts indicate the proportion of samples with detectable signature contribution.

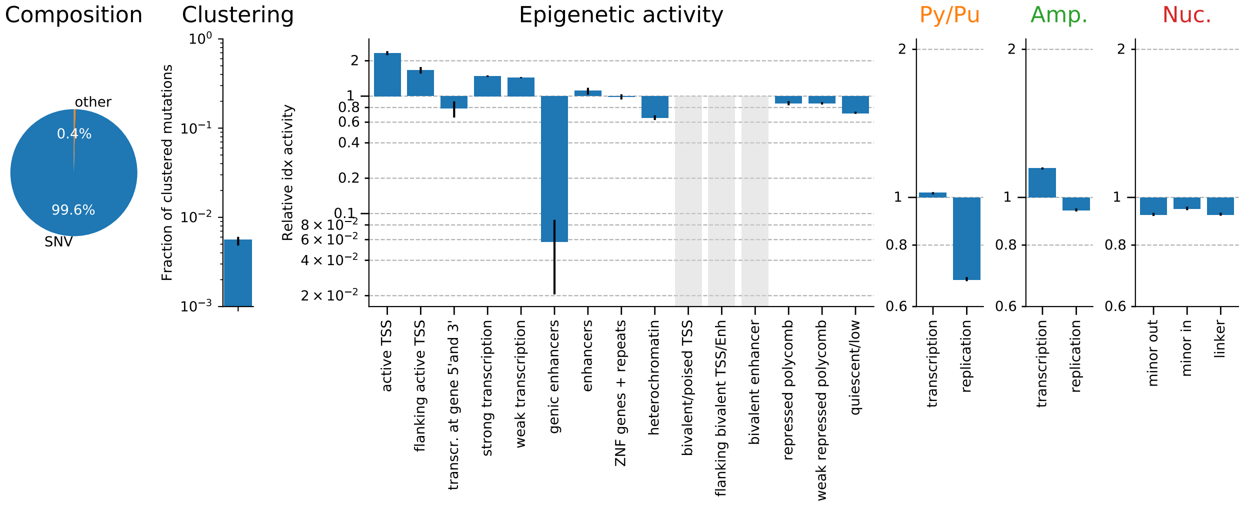

Supplementary Note 1 Fig. 60: **TS12: Signature specific tensor coefficients.** Bars and pie charts indicate maximum likelihood estimates and error bars determine 95% bootstrap confidence intervals.

TS13-N[C>K]H (AID/SHM)

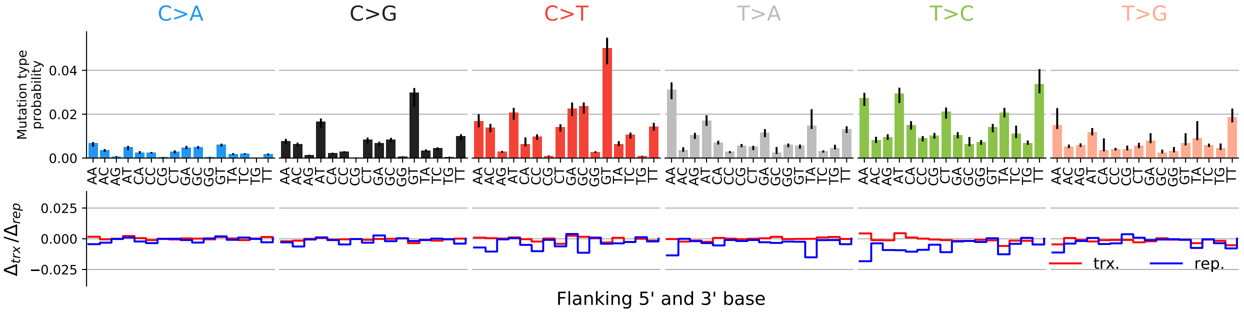

Supplementary Note 1 Fig. 61: **TS13: Single base substitution spectrum.** Bars indicate maximum likelihood estimates and error bars determine 95% bootstrap confidence intervals.

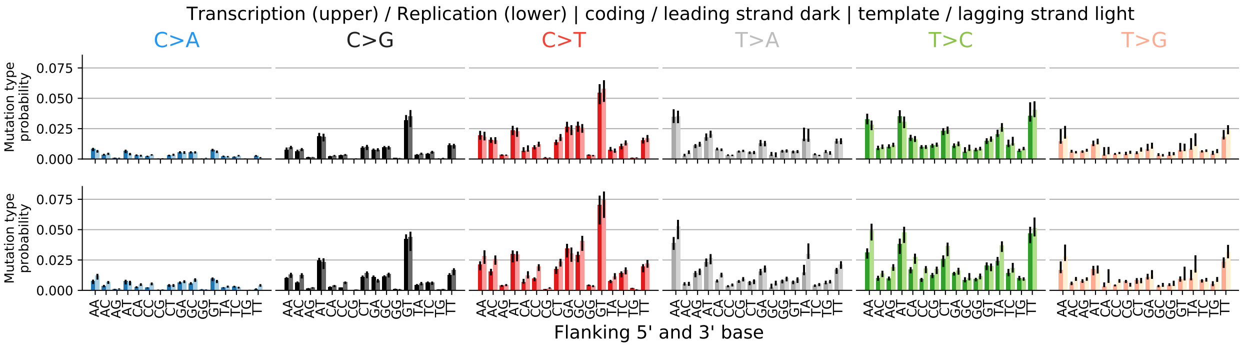

Supplementary Note 1 Fig. 62: **TS13: Single base substitution spectra for template/-coding and leading/lagging strand DNA.** Bars indicate maximum likelihood estimates and error bars determine 95% bootstrap confidence intervals.

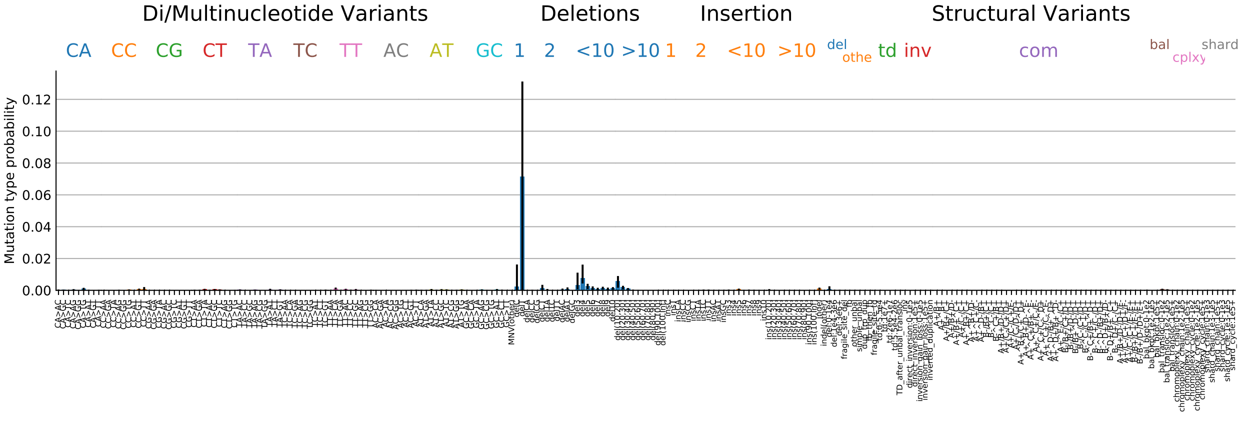

Supplementary Note 1 Fig. 63: **TS13: Spectrum other mutation types.** Bars indicate maximum likelihood estimates and error bars determine 95% bootstrap confidence intervals.

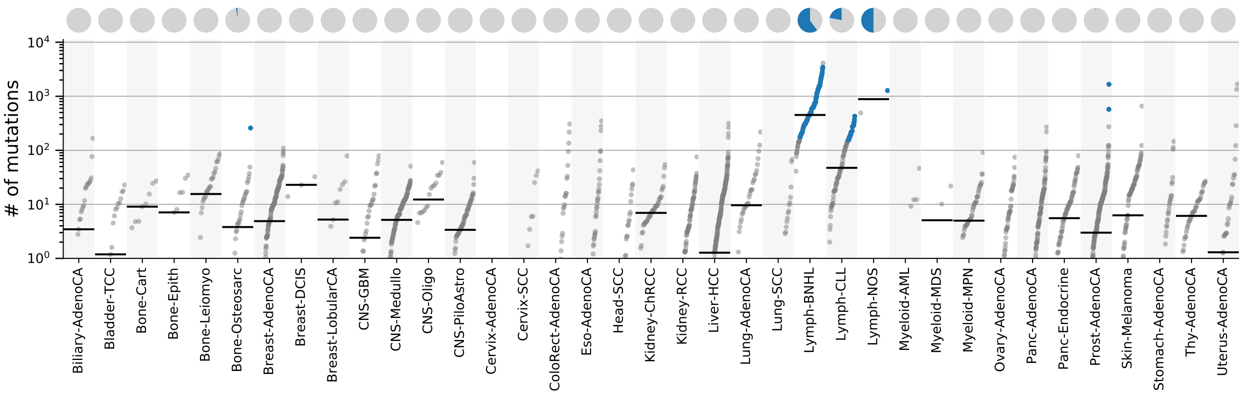

Supplementary Note 1 Fig. 64: **TS13: Signature activity in different cancer types.** Dots indicate maximum likelihood estimates and colored blue if the sample exposure exceeded the typical signature activity. Pie charts indicate the proportion of samples with detectable signature contribution.

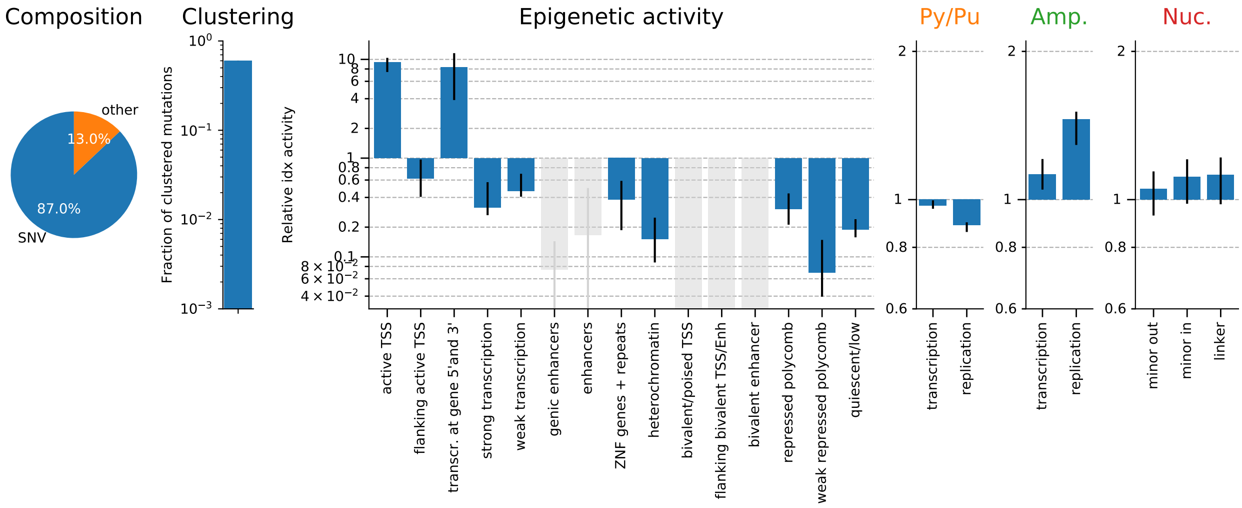

Supplementary Note 1 Fig. 65: **TS13: Signature specific tensor coefficients.** Bars and pie charts indicate maximum likelihood estimates and error bars determine 95% bootstrap confidence intervals.

TS14-W[T>V]W (POLH)

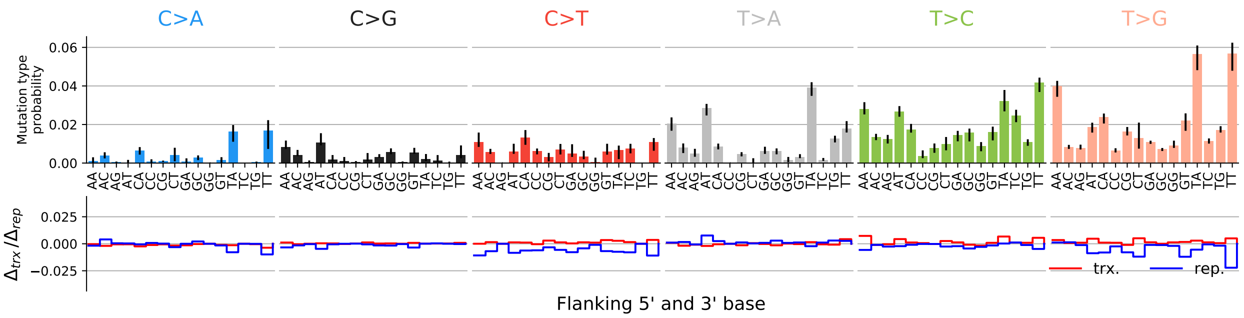

Supplementary Note 1 Fig. 66: **TS14: Single base substitution spectrum.** Bars indicate maximum likelihood estimates and error bars determine 95% bootstrap confidence intervals.

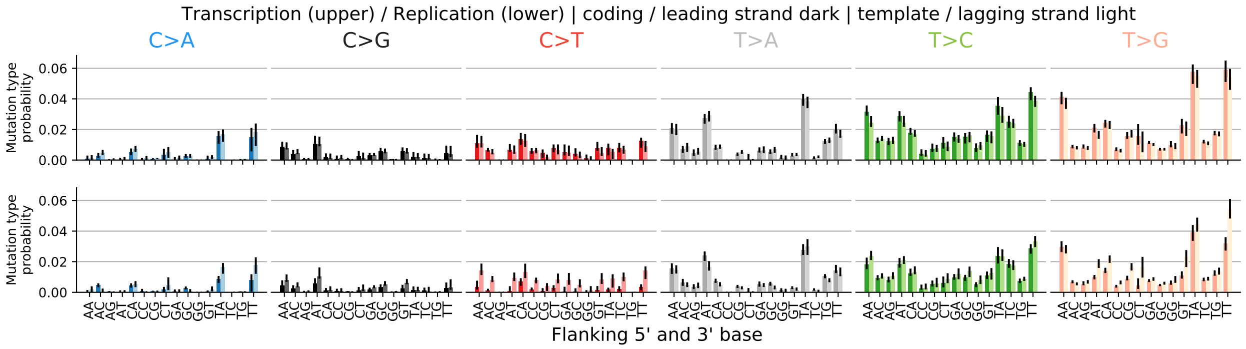

Supplementary Note 1 Fig. 67: **TS14: Single base substitution spectra for template/-coding and leading/lagging strand DNA.** Bars indicate maximum likelihood estimates and error bars determine 95% bootstrap confidence intervals.

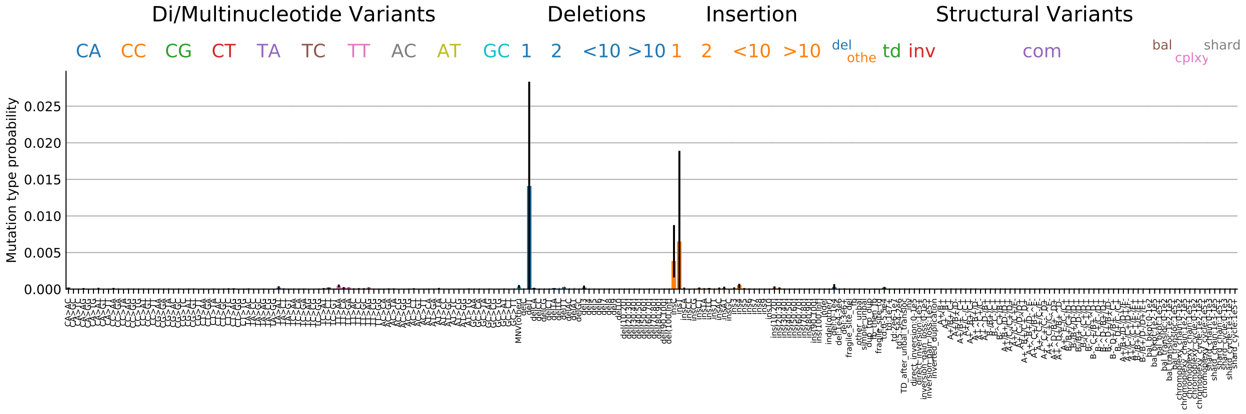

Supplementary Note 1 Fig. 68: **TS14: Spectrum other mutation types.** Bars indicate maximum likelihood estimates and error bars determine 95% bootstrap confidence intervals.

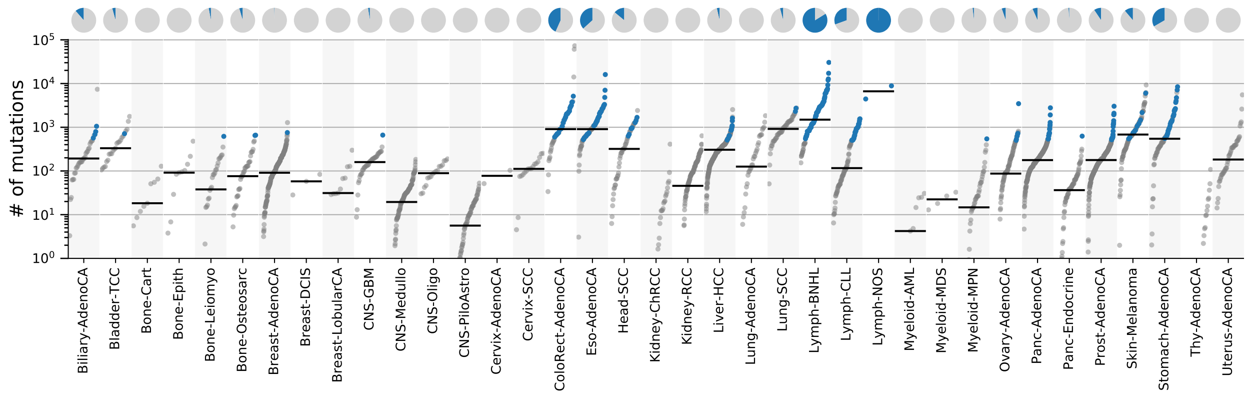

Supplementary Note 1 Fig. 69: **TS14: Signature activity in different cancer types.** Dots indicate maximum likelihood estimates and colored blue if the sample exposure exceeded the typical signature activity. Pie charts indicate the proportion of samples with detectable signature contribution.

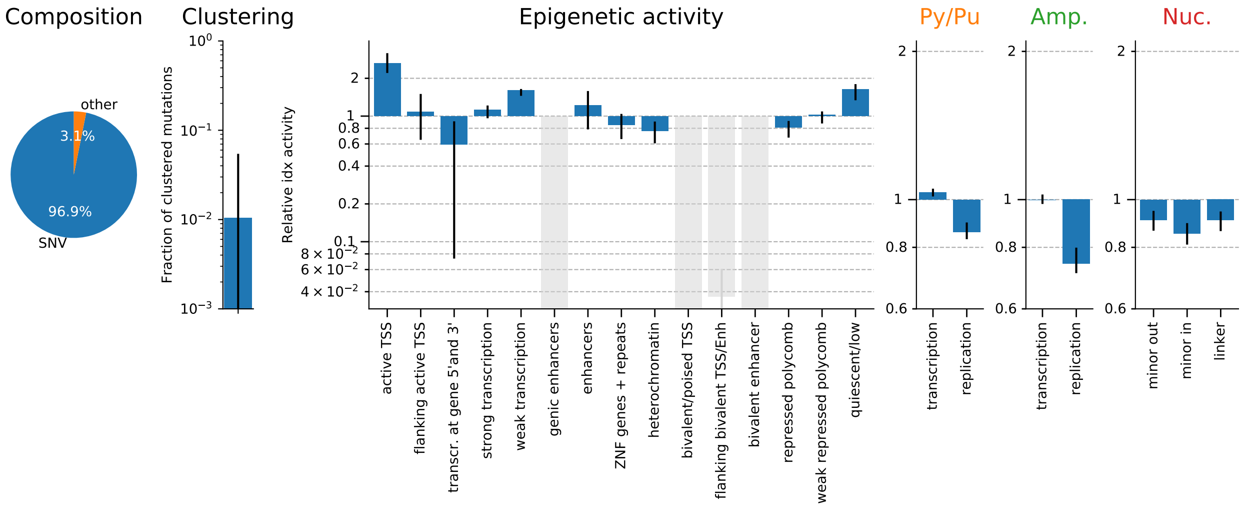

Supplementary Note 1 Fig. 70: **TS14: Signature specific tensor coefficients.** Bars and pie charts indicate maximum likelihood estimates and error bars determine 95% bootstrap confidence intervals.

TS15-G[C>T]N;ID (MMRD)

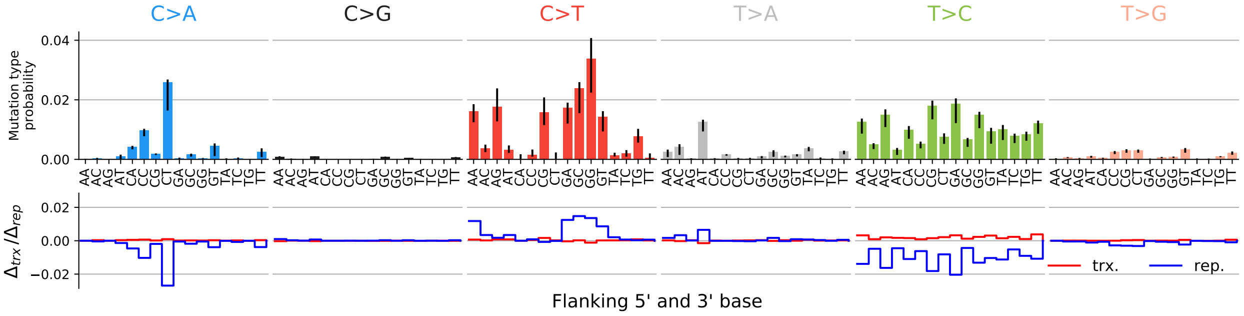

Supplementary Note 1 Fig. 71: **TS15: Single base substitution spectrum.** Bars indicate maximum likelihood estimates and error bars determine 95% bootstrap confidence intervals.

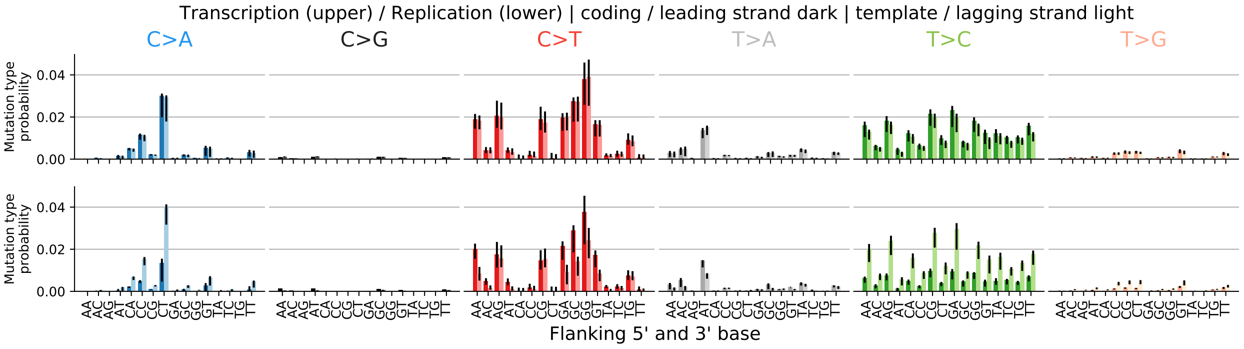

Supplementary Note 1 Fig. 72: **TS15: Single base substitution spectra for template/-coding and leading/lagging strand DNA.**

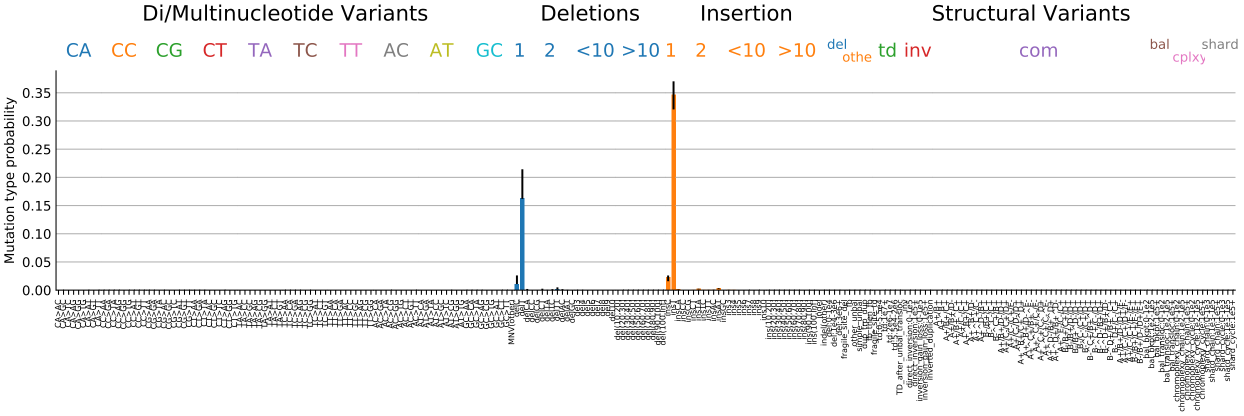

Supplementary Note 1 Fig. 73: **TS15: Spectrum other mutation types.** Bars indicate maximum likelihood estimates and error bars determine 95% bootstrap confidence intervals.

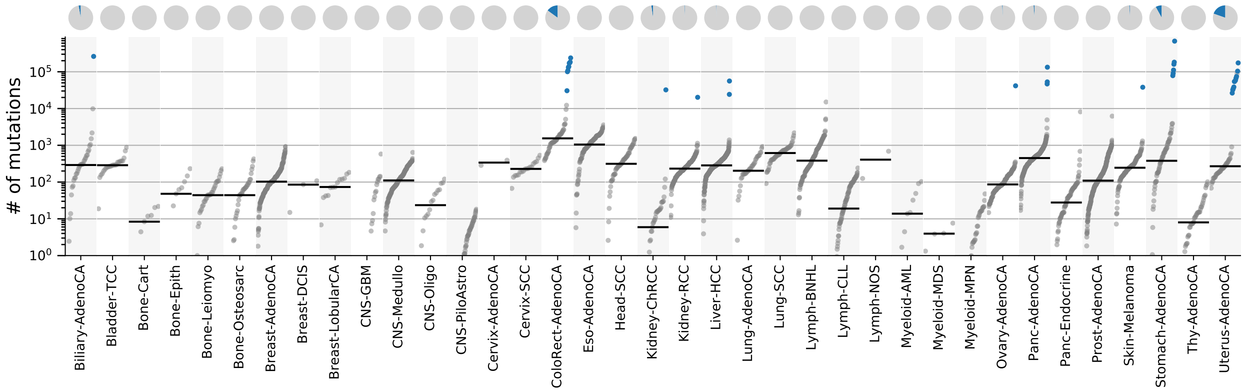

Supplementary Note 1 Fig. 74: **TS15: Signature activity in different cancer types.** Dots indicate maximum likelihood estimates and colored blue if the sample exposure exceeded the typical signature activity. Pie charts indicate the proportion of samples with detectable signature contribution.

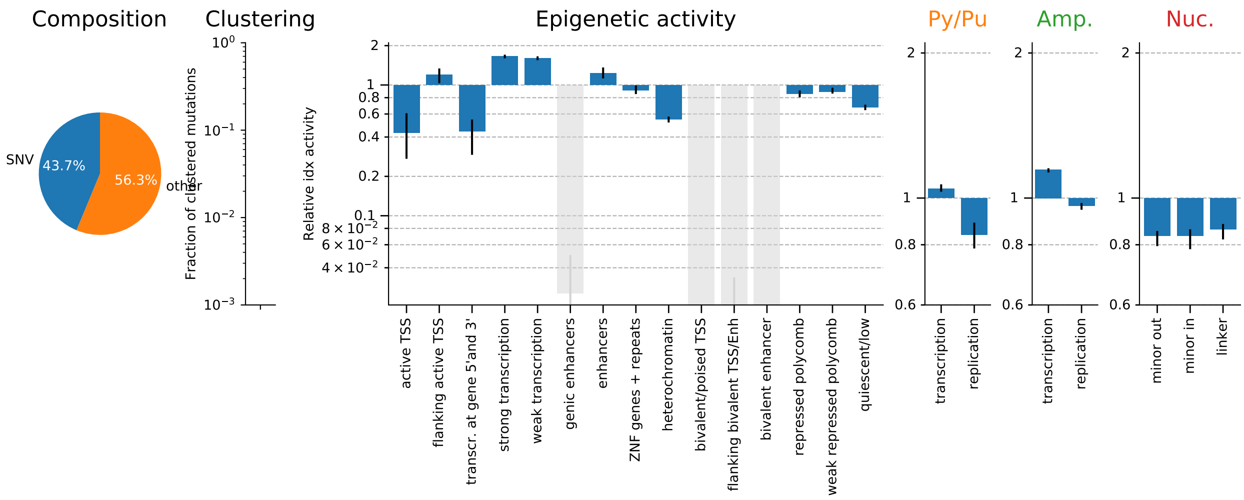

Supplementary Note 1 Fig. 75: **TS15: Signature specific tensor coefficients.** Bars and pie charts indicate maximum likelihood estimates and error bars determine 95% bootstrap confidence intervals.

TS16-N[C>A]T;ID (MMRD:POLE-exo)

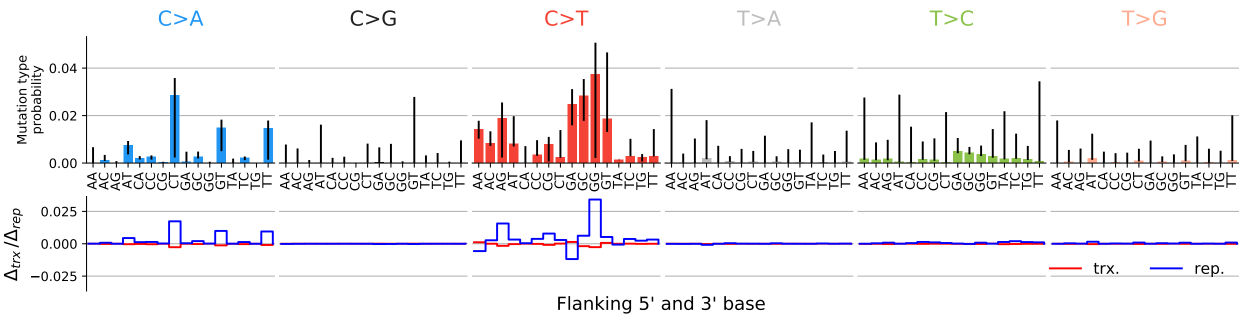

Supplementary Note 1 Fig. 76: **TS16: Single base substitution spectrum.** Bars indicate maximum likelihood estimates and error bars determine 95% bootstrap confidence intervals.

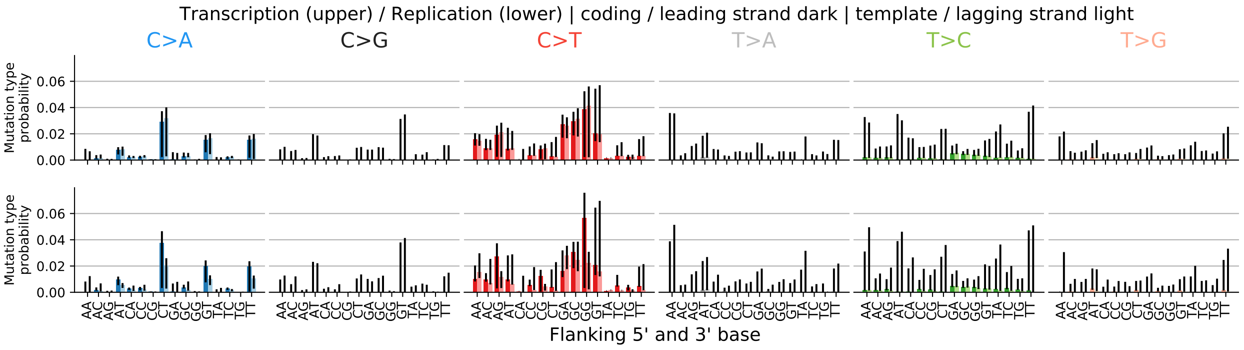

Supplementary Note 1 Fig. 77: **TS16: Single base substitution spectra for template/-coding and leading/lagging strand DNA.** Bars indicate maximum likelihood estimates and error bars determine 95% bootstrap confidence intervals.

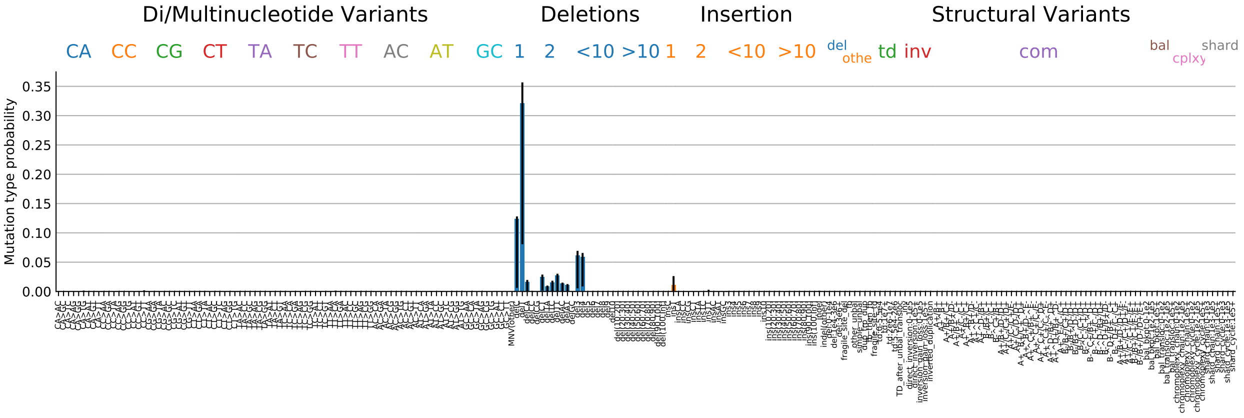

Supplementary Note 1 Fig. 78: **TS16: Spectrum other mutation types.** Bars indicate maximum likelihood estimates and error bars determine 95% bootstrap confidence intervals.

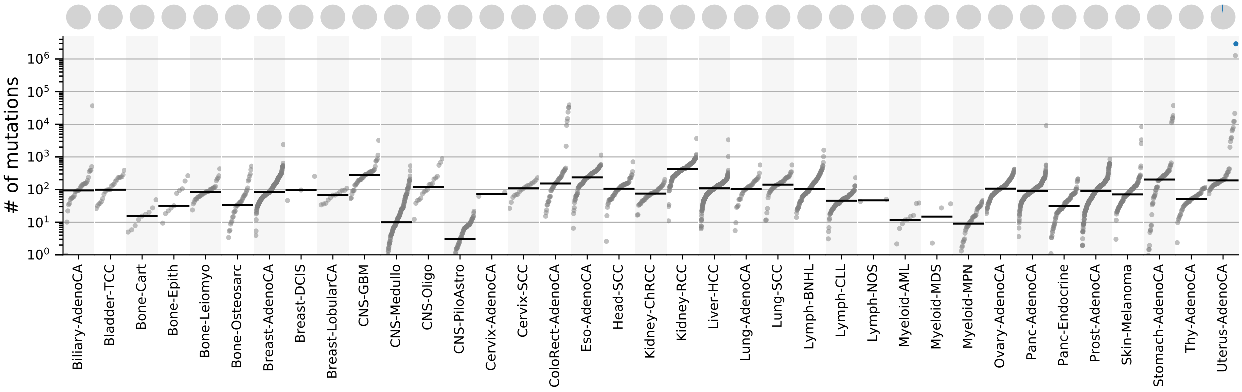

Supplementary Note 1 Fig. 79: **TS16: Signature activity in different cancer types.** Dots indicate maximum likelihood estimates and colored blue if the sample exposure exceeded the typical signature activity. Pie charts indicate the proportion of samples with detectable signature contribution.

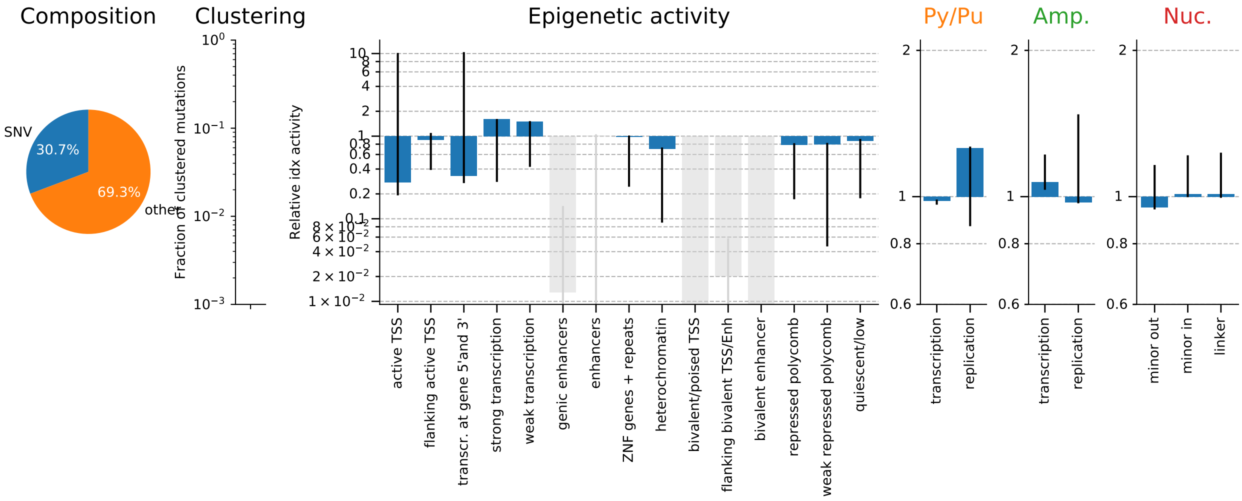

Supplementary Note 1 Fig. 80: **TS16: Signature specific tensor coefficients.** Bars and pie charts indicate maximum likelihood estimates and error bars determine 95% bootstrap confidence intervals.

TS17-T[C>A]T (POLE-exo)

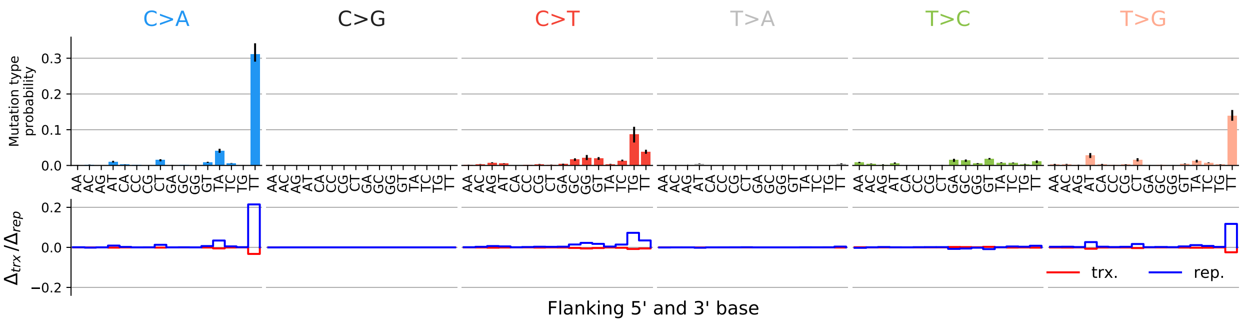

Supplementary Note 1 Fig. 81: **TS17: Single base substitution spectrum.** Bars indicate maximum likelihood estimates and error bars determine 95% bootstrap confidence intervals.

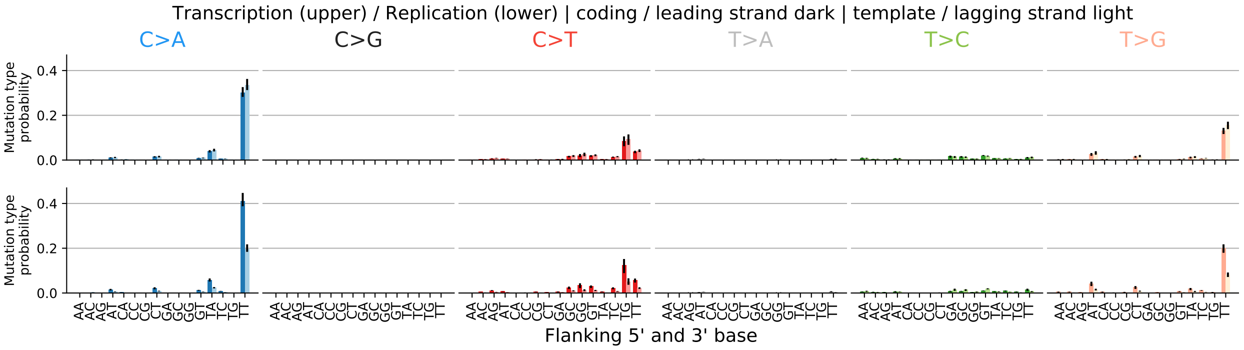

Supplementary Note 1 Fig. 82: **TS17: Single base substitution spectra for template/-coding and leading/lagging strand DNA.** Bars indicate maximum likelihood estimates and error bars determine 95% bootstrap confidence intervals.

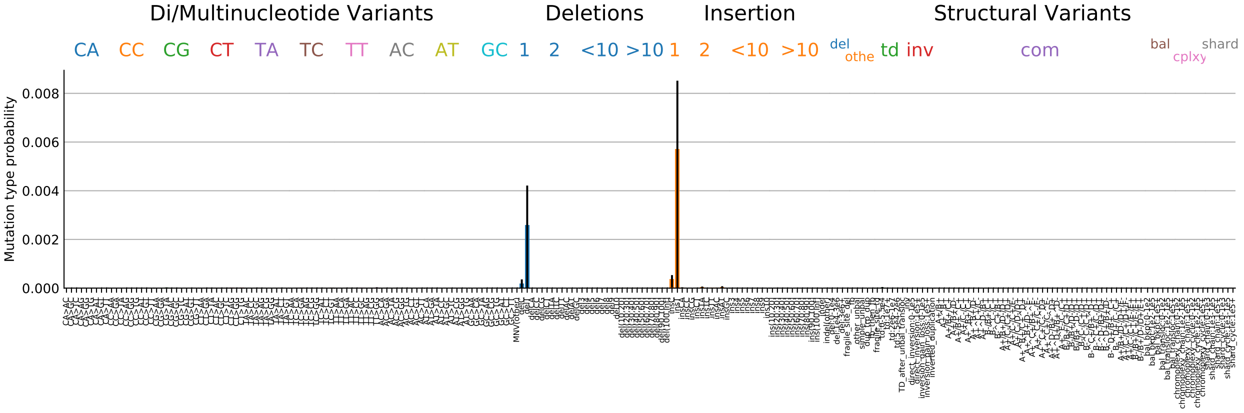

Supplementary Note 1 Fig. 83: **TS17: Spectrum other mutation types.** Bars indicate maximum likelihood estimates and error bars determine 95% bootstrap confidence intervals.

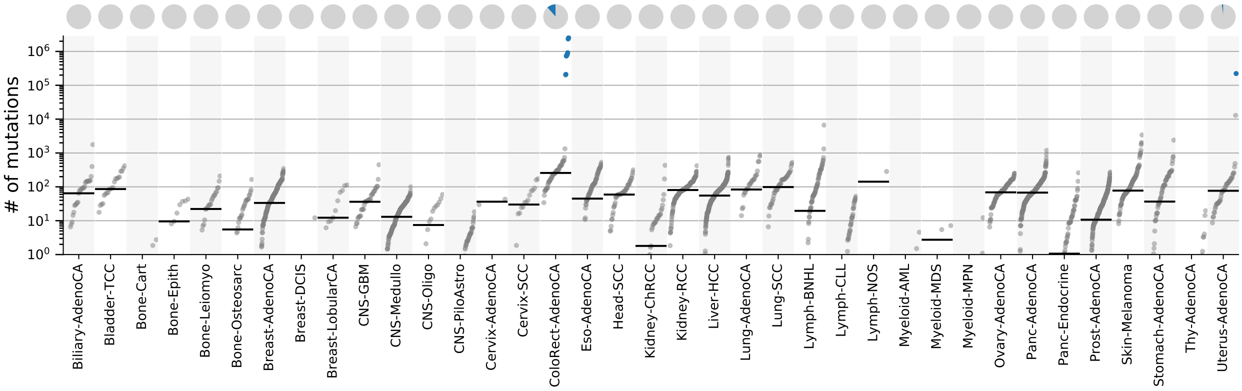

Supplementary Note 1 Fig. 84: **TS17: Signature activity in different cancer types.** Dots indicate maximum likelihood estimates and colored blue if the sample exposure exceeded the typical signature activity. Pie charts indicate the proportion of samples with detectable signature contribution.

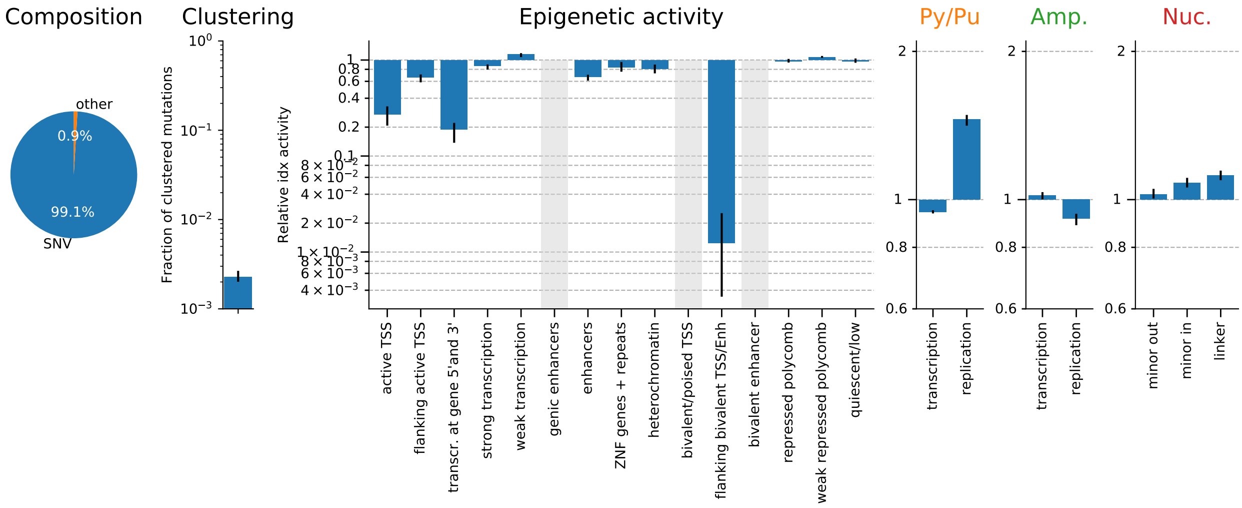

Supplementary Note 1 Fig. 85: **TS17: Signature specific tensor coefficients.** Bars and pie charts indicate maximum likelihood estimates and error bars determine 95% bootstrap confidence intervals.

TS18-N[C>A]W (BERD/MUTYH)

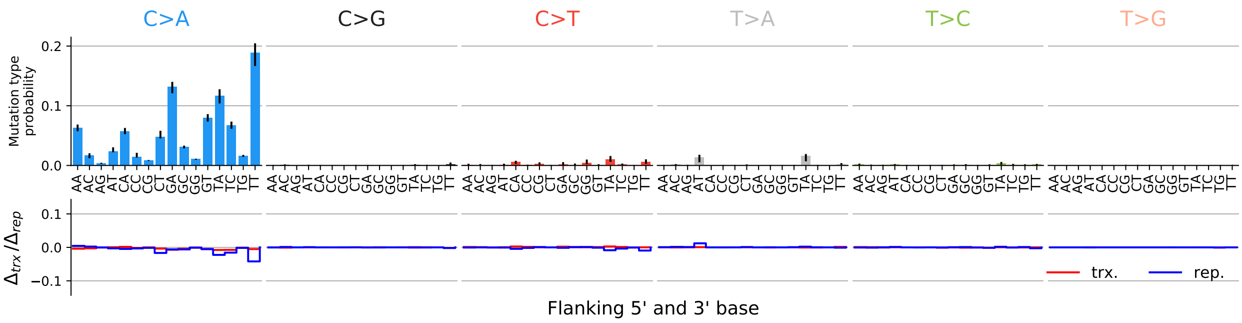

Supplementary Note 1 Fig. 86: **TS18: Single base substitution spectrum.** Bars indicate maximum likelihood estimates and error bars determine 95% bootstrap confidence intervals.

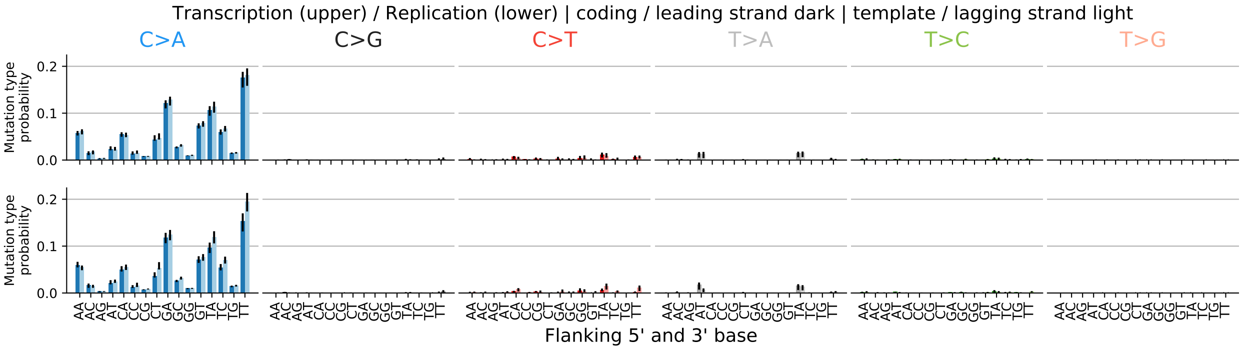

Supplementary Note 1 Fig. 87: **TS18: Single base substitution spectra for template/-coding and leading/lagging strand DNA.** Bars indicate maximum likelihood estimates and error bars determine 95% bootstrap confidence intervals.

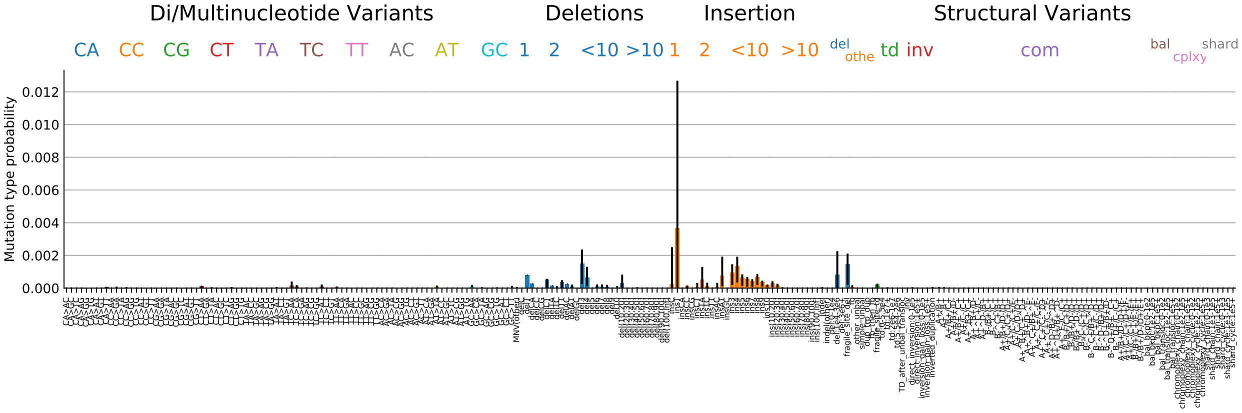

Supplementary Note 1 Fig. 88: **TS18: Spectrum other mutation types.** Bars indicate maximum likelihood estimates and error bars determine 95% bootstrap confidence intervals.

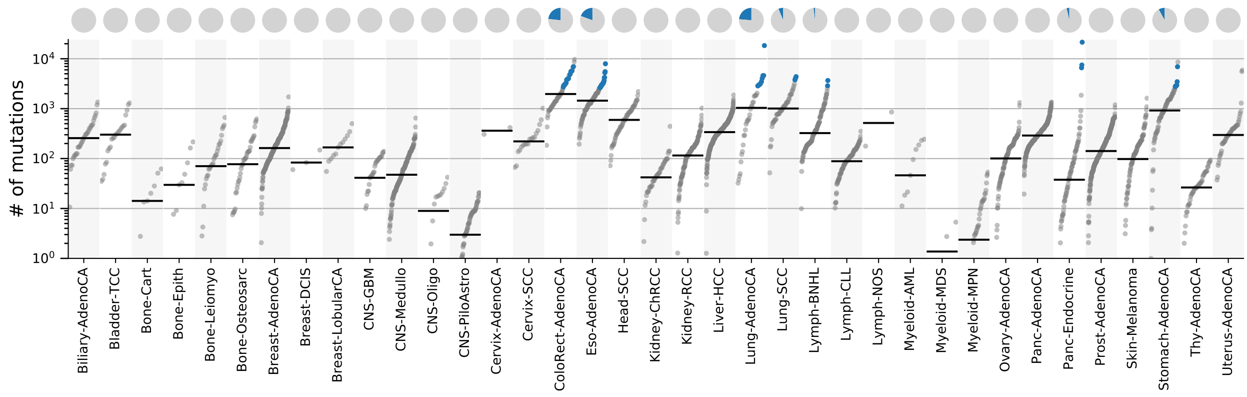

Supplementary Note 1 Fig. 89: **TS18: Signature activity in different cancer types.** Dots indicate maximum likelihood estimates and colored blue if the sample exposure exceeded the typical signature activity. Pie charts indicate the proportion of samples with detectable signature contribution.

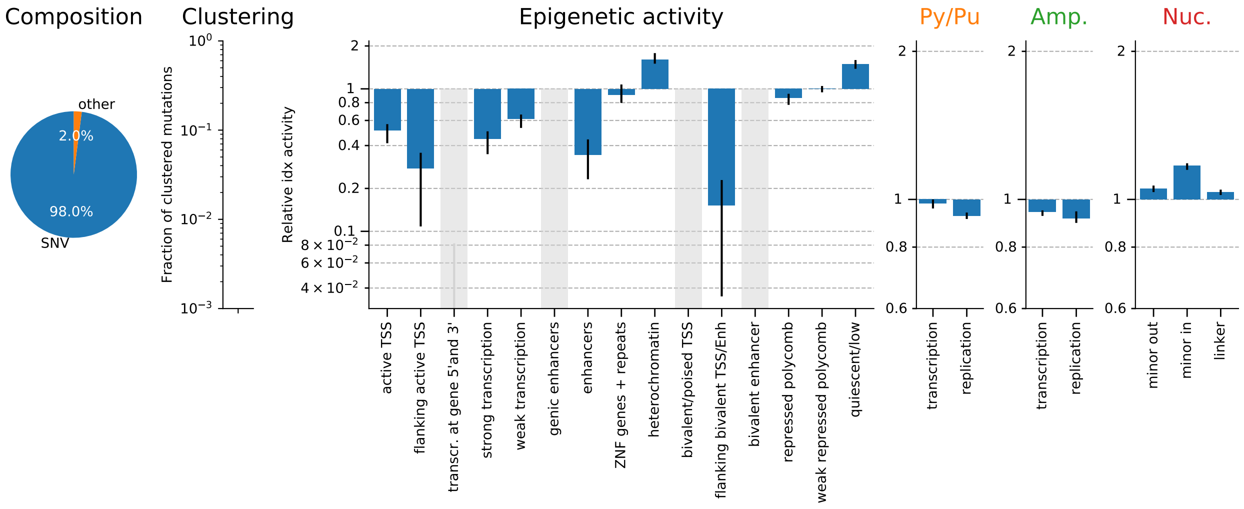

Supplementary Note 1 Fig. 90: **TS18: Signature specific tensor coefficients.** Bars and pie charts indicate maximum likelihood estimates and error bars determine 95% bootstrap confidence intervals.

TS19-N[N>N]N;SV (HRD/BRCA)

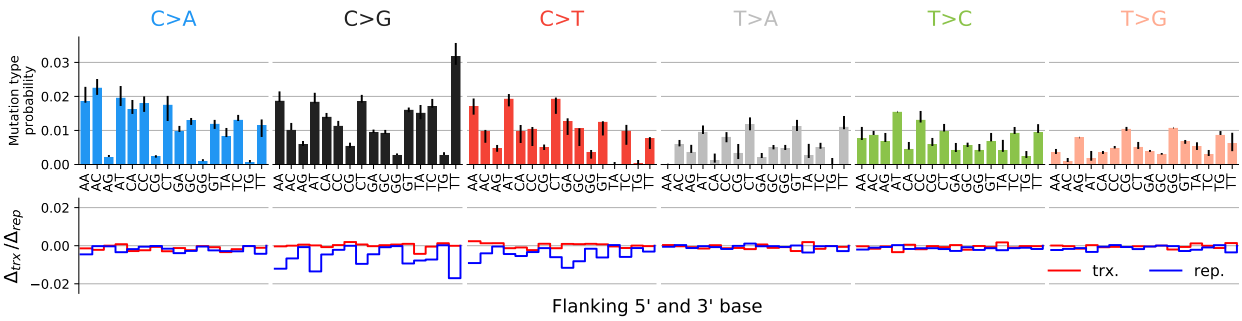

Supplementary Note 1 Fig. 91: **TS19: Single base substitution spectrum.** Bars indicate maximum likelihood estimates and error bars determine 95% bootstrap confidence intervals.

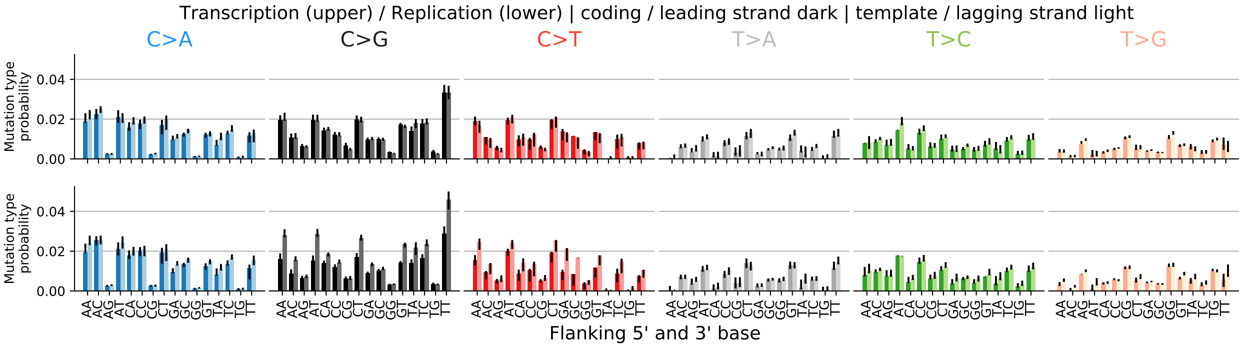

Supplementary Note 1 Fig. 92: **TS19: Single base substitution spectra for template/-coding and leading/lagging strand DNA.** Bars indicate maximum likelihood estimates and error bars determine 95% bootstrap confidence intervals.

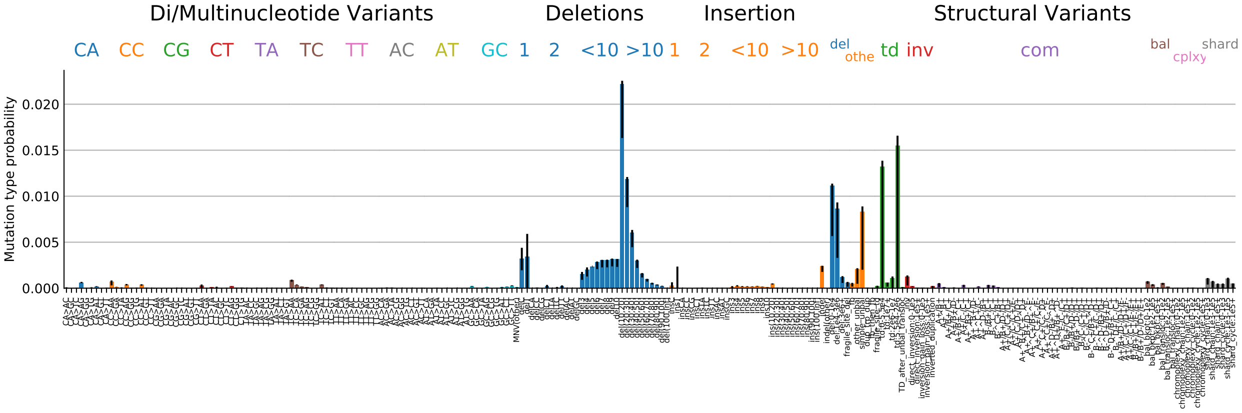

Supplementary Note 1 Fig. 93: **TS19: Spectrum other mutation types.** Bars indicate maximum likelihood estimates and error bars determine 95% bootstrap confidence intervals.

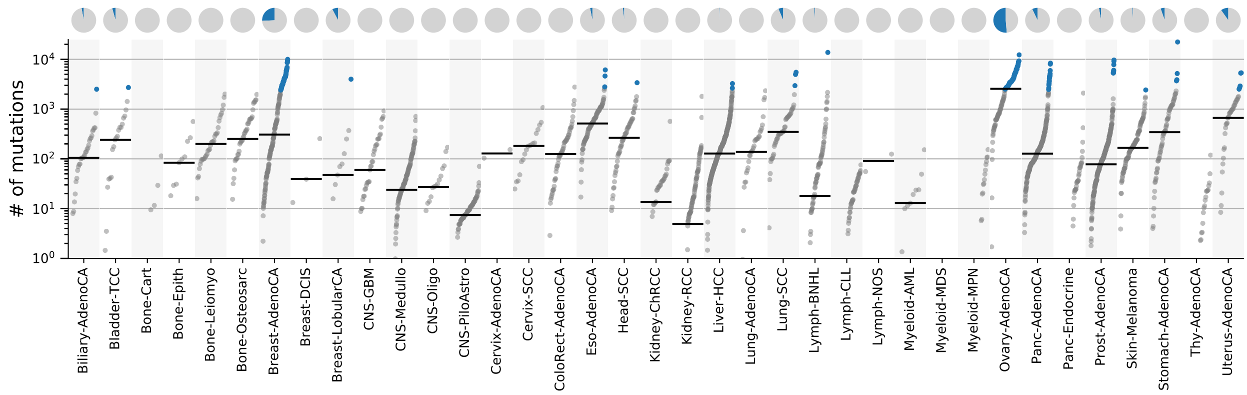

Supplementary Note 1 Fig. 94: **TS19: Signature activity in different cancer types.** Dots indicate maximum likelihood estimates and colored blue if the sample exposure exceeded the typical signature activity. Pie charts indicate the proportion of samples with detectable signature contribution.

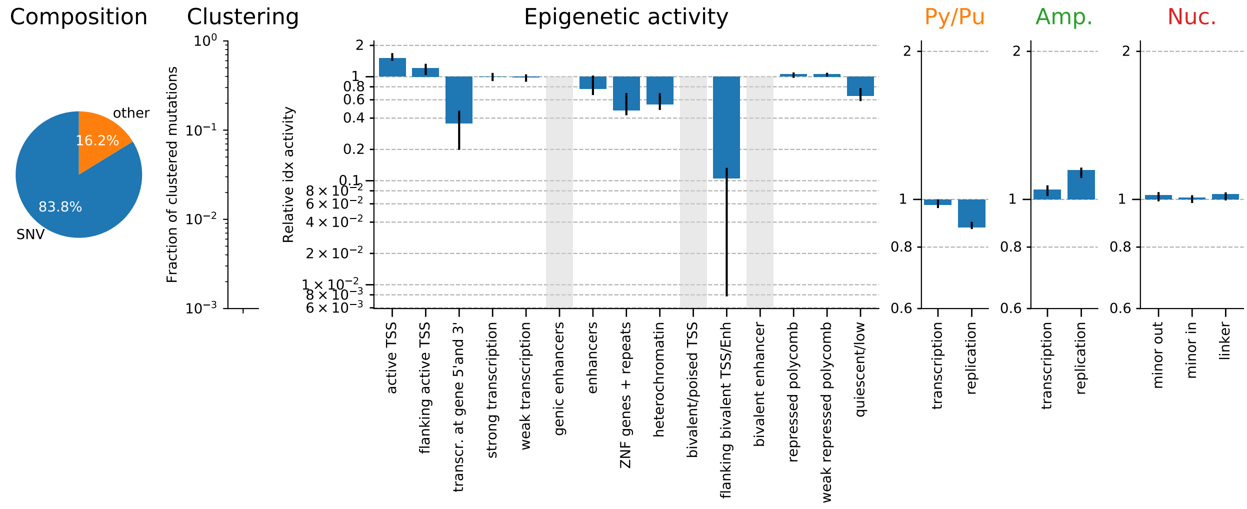

Supplementary Note 1 Fig. 95: **TS19: Signature specific tensor coefficients.** Bars and pie charts indicate maximum likelihood estimates and error bars determine 95% bootstrap confidence intervals.

TS20-N[T>G]T (unknown/5FU)

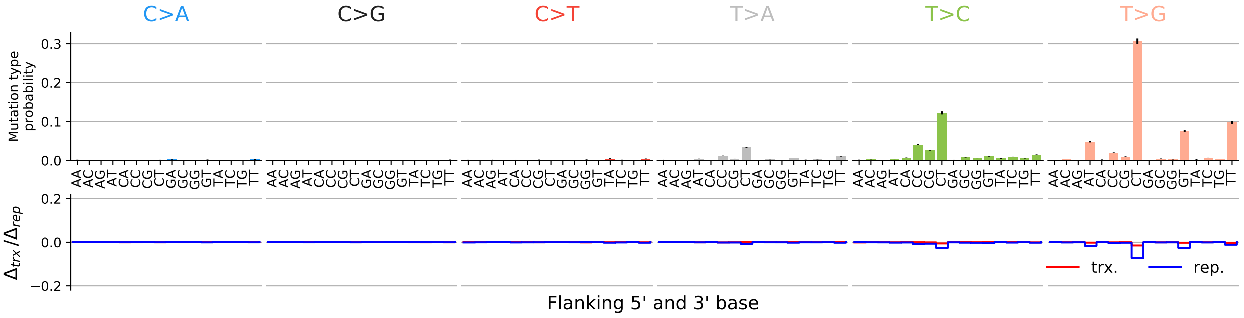

Supplementary Note 1 Fig. 96: **TS20: Single base substitution spectrum.** Bars indicate maximum likelihood estimates and error bars determine 95% bootstrap confidence intervals.

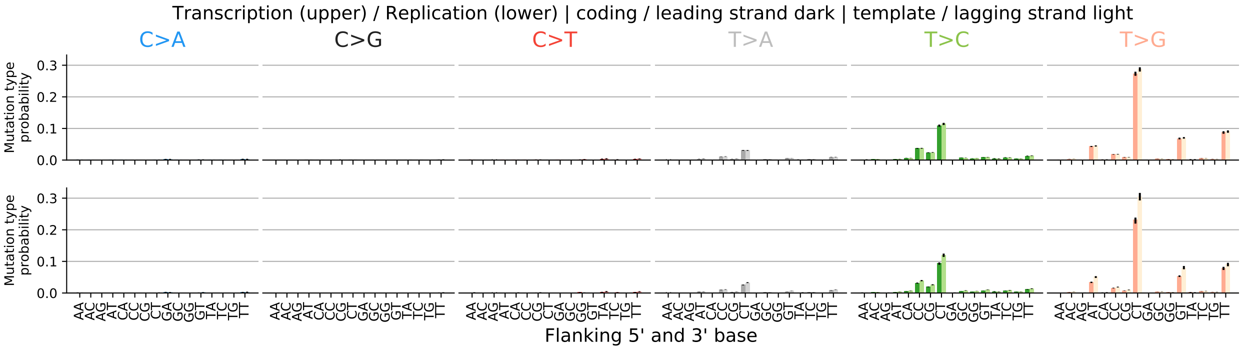

Supplementary Note 1 Fig. 97: **TS20: Single base substitution spectra for template/-coding and leading/lagging strand DNA.** Bars indicate maximum likelihood estimates and error bars determine 95% bootstrap confidence intervals.

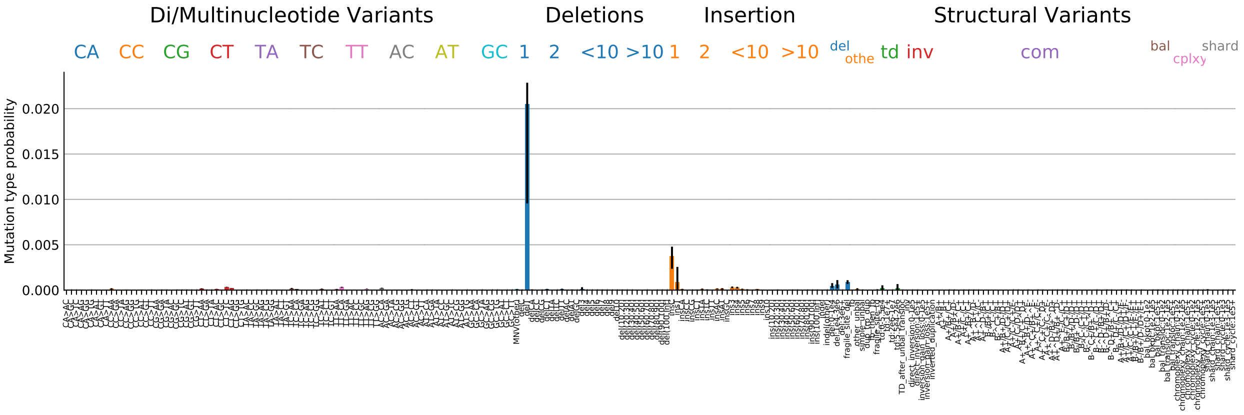

Supplementary Note 1 Fig. 98: **TS20: Spectrum other mutation types.** Bars indicate maximum likelihood estimates and error bars determine 95% bootstrap confidence intervals.

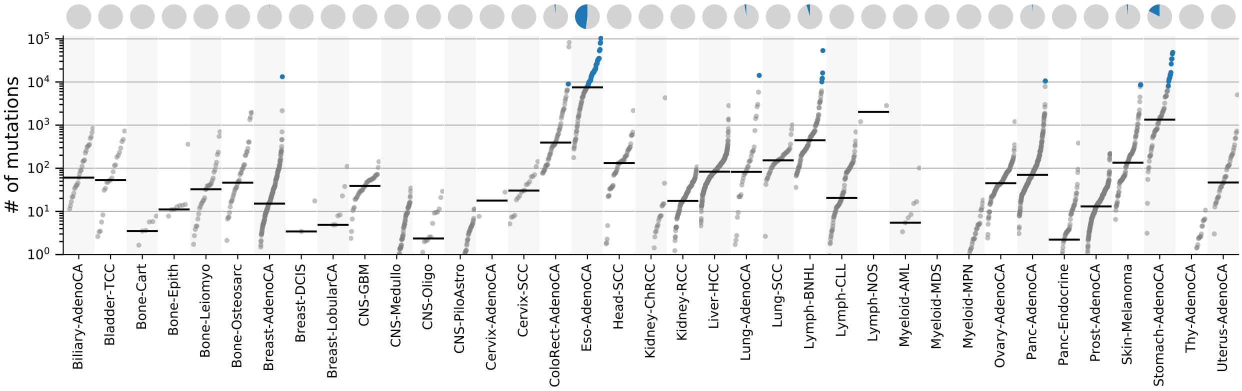

Supplementary Note 1 Fig. 99: **TS20: Signature activity in different cancer types.** Dots indicate maximum likelihood estimates and colored blue if the sample exposure exceeded the typical signature activity. Pie charts indicate the proportion of samples with detectable signature contribution.

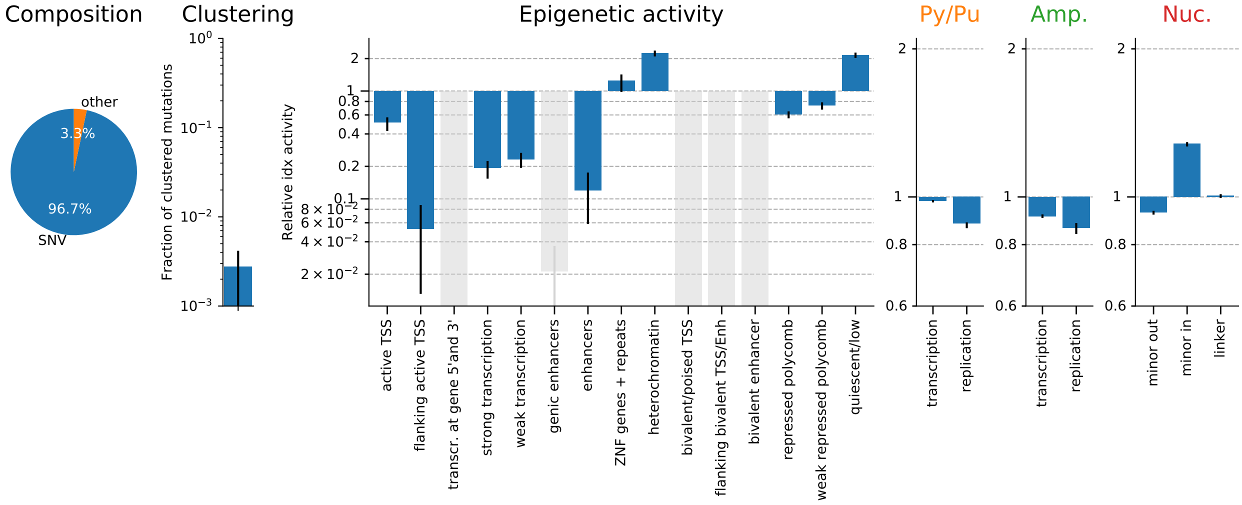

Supplementary Note 1 Fig. 100: **TS20: Signature specific tensor coefficients.** Bars and pie charts indicate maximum likelihood estimates and error bars determine 95% bootstrap confidence intervals.

3 Supplementary Note 2: TensorSignatures Manual

TensorSignatures is a tensor factorisation framework for mutational signature analysis, which in contrast to other methods, deciphers mutational processes not only in terms of mutational spectra, but also assess their properties with respect to various genomic variables, allows the inclusion of different mutation types and integrates a robust noise model to perform the inference.
TensorSignatures is a young project and breaking changes are to be expected. We keep a changelog and it will have possible breakage clearly documented.

Installing TensorSignatures

TensorSignatures makes use of the TensorFlow 1.5.x framework requiring the user to install a separate package to enable GPU support, i.e. `tensorflow-gpu` instead of `tensorflow`. We highly recommend to install TensorSignatures into an environment with `tensorflow-gpu`, as the tensor computations greatly benefit from GPU-acceleration.

Installation via GitHub

To obtain the most recent version of TensorSignatures, we recommend to download the repository directly from GitHub and to install the package into a virtual environment. To get started, clone the repository by executing the following commands in your terminal

```
$ git clone https://github.com/gerstung-lab/tensorsignatures.git && \
cd tensorsignatures
```

Then, create a new virtual environment and install all dependencies. If you have access to a GPU with cuda support use `requirements-gpu.txt` instead of `requirements.txt`

```
$ python -m venv env
$ source env/bin/activate
$ pip install --upgrade pip setuptools wheel && \
pip install -r requirements.txt
```

Finally, install TensorSignatures.

```
$ python setup.py install
```

Installation via Pypi

To install `tensorsignatures` via Pypi simply type

```
$ pip install tensorsignatures
```

into your shell.

Installation via Docker

To run TensorSignatures within a docker environment clone the the repository

```
$ git clone https://github.com/gerstung-lab/tensorsignatures.git
$ cd tensorsignatures
```

and then start the image using `docker-compose`.

```
$ docker-compose up --build
```

This spins up a jupyter server including notebooks with tutorials on `http://localhost:8889`.

Quick Start

Running TensorSignatures involves three steps: preparing the input data, i.e. creating the mutation count tensor as well as the mutation count matrix, computing a trinucleotide normalisation to account for differences in the nucleotide composition of different genomic regions, and running TensorSignatures.

Step 1: Data preparation

Preparing the input data for TensorSignatures involves creating the single base substitution count tensor and the other mutation type count matrix with multinucleotide variants, deletions and insertions (currently we do not provide a automated way of generating a structural variant table yet). Despite the fact that TensorSignatures is written in Python, this part of the pipeline runs in `R` and depends on the `Bioconductor` packages `VariantAnnotation` and `rhdf5`.

Preparing input data using docker We provide a docker image that contains all `R` and `bioconductor` dependencies to create the variant tensor and the other mutation type matrix. To use it, pull the image from docker. Note that the image is approximately 5 GB large.

```
$ docker pull sagar87/tensorsignatures-data:latest
```

To use the image switch into the folder containing your VCF data. Then run image using the following command and supply the VCF files as well as the name of the `hdf5` output file (must be the last argument) as arguments.

```
$ docker run -v $PWD:/usr/src/app/mount sagar87/tensorsignatures-data \
<vcf1.vcf> <vcf2.vcf> ... <vcfn.vcf> <output.h5>
```

Then continue with Step 2 (Sec. 3).

Preparing the input data using a custom installation Make sure you have `R3.4.x` (!) and the packages `VariantAnnotation` and `rhdf5` installed. You can install them, if necessary, by executing

```
$ Rscript -e "source('https://bioconductor.org/biocLite.R'); \
biocLite('VariantAnnotation')"
```

and

```
$ Rscript -e "source('https://bioconductor.org/biocLite.R'); \
biocLite('rhdf5')"
```

from your command line. Then, download the following files and place them in the same directory:

- `Constants.RData` (contains `GRanges` objects that annotate transcription/replication orientation, nucleosomal and epigenetic states)
- `mutations.R` (all required functions to partiton SNVs, MNVs and Indels)
- `processVcf.R` (loads vcf files and creates the SNV count tensor, MNV and indel count matrix; eventually needs custom modification to make the script run on your vcfs.)
- `genome.zip`.

To obtain the SNV count tensor and the matrices containing other mutation types, execute `processVcf.R` and pass the VCF files you want to convert, as well as a name for an output `hdf5` file as command line arguments, e.g.

```
$ Rscript processVcf.R <vcf1.vcf> <vcf2.vcf> ... <vcfn.vcf>\
<output.h5>
```

In case of errors please check wether you have correctly specified paths in line 6-8. Also, take a look at the `readVcfSave` function and adjust it when it fails.

Step 2: Computing trinucleotide normalisation

TensorSignatures requires a trinucleotide normalisation constant to account for differences in the nucleotide composition of genomic states. To compute it, invoke the prep sub routine of TensorSignatures and pass the `hd5` file from Step 1 (Sec. 3) as well as the path for the output file as positional arguments to the programme.

```
$ tensorsignatures prep <output.h5> <tsdata.h5>
```

Step 3: Run TensorSignatures

There are two ways to run TensorSignatures using either the `refit` option, which fits the exposures of a set of pre-defined signatures extracted from the PCAWG cohort to your dataset, or via the `train` subroutine, that performs a denovo extraction of tensor signatures. Refitting tensor signatures is computationally fast but does not allow to discover new signatures, while extracting new signatures from scratch is computationally intensive (GPU required) and requires ideally larger numbers of samples. For most use cases, with a small number of samples, we advice to use the refit option:

```
$ tensorsignatures --verbose refit tsData.h5 refit.pkl -n
```

To run a denovo extraction use

```
$ tensorsignatures --verbose train tsData.h5 denovo.pkl <rank> \
-k <size> -n -ep <epochs>
```

where `rank` specifies the decomposition rank, `size` controls the dispersion of the model, and `epochs` the number of desired epochs to fit the model. TensorSignatures outputs value of the objective function (log likelihood) that is minimised during training as well as the change of the objective during an epoch interval (`delta`). When deciding on the number of epochs to train the model ensure that it is sufficiently large such that the objective function converges, i.e. the `delta` value is close to, or fluctuates around zero. For more information on how to run TensorSignatures in a practical setting see Sec. (3). Running TensorSignatures will yield a `pickle` dump which can subsequently inspected using the `tensorsignatures` package (3).

Tutorials

TensorSignatures extracts mutational signatures and their genomic properties from a mutation count tensor that partitions single base substitutions with respect to a multitude of genomic states. Moreover, the algorithm links other variant types to these signatures by taking into account a secondary mutation matrix. In following tutorials, we want to convey an intuition for working with such highdimensional data, and explain the usage of the `tensorsignatures` API and command line interface (CLI).

Understanding the mutation count tensor

Let us start by creating a simulated mutation count tensor to better understand the structure of this data type. The `data` module of `tensorsignatures` provides a `TensorSignatureData` class which allows us to simulate such data. To do so, we import the package and create a `TensorSignatureData` instance.

```
> import tensorsignatures as ts
> data = ts.TensorSignatureData(
    seed=573, # set a seed for reproducibility
    rank=5, # number of signatures
    samples=100, # number of samples
    dimensions=[3, 5], # number of arbitrary genomic dimensions
    mutations=1000)
```

This command generates data from five signatures (`rank`) to simulate 100 genomes (`samples`) each with 1000 mutations (`mutations`). By passing the list [3, 5] to the `dimensions` argument, we create two additional genomic dimensions[1] with 3 and 5 states respectively. To obtain the SNV count tensor, we invoke the `snv` method of `data`, which returns the single base substitution count tensor.

```
> snv = data.snv()
```

Similarly, we can extract a simulated matrix of other mutation counts by invoking the `other()` method.

```
> other = data.other()
```

[1] in addition to the dimensions specifying transcription and replication

The mutation count tensor is a multidimensional array with a specific structure The `snv` object is simply a 6-dimensional `numpy` array,

```
> snv.ndim
6
```

whose `shape` attribute is a tuple of integers indicating the size, i.e. the number of states, of the array in each dimension.

```
> snv.shape
(3, 3, 3, 5, 96, 100)
```

TensorSignatures expects the structure of the count tensor to follow a specific convention: the first and second dimension (`snv.shape[0]` and `snv.shape[1]`) split counts by transcription and replication strand, following dimensions partition single base substitution by genomic factors, and the penultimate (`snv.shape[-2]`) and last dimension (`snv.shape[-1]`) represent substitution types and samples respectively. Table 1 summarises the structure of the count tensor.

Extracting the single base substutions from specific genomic states We index the SNV tensor like any other `numpy` array. For example, to obtain variants from template and leading strands, and from the "unassigned" state of additional genomic dimensions, we simply index the tensor with `snv[0, 1, 0, 0, :, :]` which returns a two dimensional array with mutation types along the first axis and samples along the other.

```
> slice = snv[0, 1, 0, 0, :, :]
> slice.shape
(96, 100)
```

Note, that we can reconstruct the $p \times n$ mutation count matrix, which usually serves as an input for conventional mutational signature analysis, by summing over all dimensions except the last two (representing single base substitution types and samples respectively). The following code illustrates this operation.

```
> collapsed = snv.sum(axis=(0, 1, 2, 3))
> collapsed.shape
(96, 100)
```

Another useful technique is to first index a specific state, and then to sum over all other dimensions to exclude. This allows us to extract the spectra from specific genomic states, for example, to extract all coding and template strand mutations from the tensor we would simply run

```
> coding = snv[0].sum(axis=(0, 1, 2, 4))
> template = snv[1].sum(axis=(0, 1, 2, 4))
```

of course this also works for any other dimension, for example, leading and lagging strand mutations maybe extracted as follows.

```
> leading = snv[:, 0].sum(axis=(0, 1, 2, 4))
> lagging = snv[:, 1].sum(axis=(0, 1, 2, 4))
```

Table 1: The structure of the SNV count tensor.

Dimension	Size	Index	State / Variants
Transcription	3	0	Coding strand
		1	Template strand
		2	Unassigned
Replication	3	0	Leading strand
		1	Lagging strand
		2	Unassigned
First genomic dimension (eg. epigenetic states)	t+1	0	Unassigned
		1	State 1
		...	...
		t	State t
Last genomic dimension (eg. nucleosomal states)	r+1	0	Unassigned
		1	State 1
		...	
		r+1	State r
Single base substitution types	p=96	0	A[C>A]A
		1	A[C>A]C
		...	...
		p	T[T>C]T
Samples	n	0	Sample 1
		...	
		n	Sample n

To understand how they differ we may plot them,

```
> fig, axes = plt.subplots(2, 2, sharey=True)
> axes[0, 0].bar(np.arange(96), coding, color=ts.DARK_PALETTE)
> axes[0, 0].set_title('Pooled coding strand mutations')
> axes[0, 1].bar(np.arange(96), template, color=ts.DARK_PALETTE)
> axes[0, 1].set_title('Pooled template strand mutations')
> axes[1, 0].bar(np.arange(96), leading, color=ts.DARK_PALETTE)
> axes[1, 0].set_title('Pooled leading strand mutations')
> axes[1, 1].bar(np.arange(96), lagging, color=ts.DARK_PALETTE)
> axes[1, 1].set_title('Pooled lagging strand mutations')
> plt.tight_layout()
```

which reveals that some variant types, e.g. C>A (blue), C>T (red) and T>A (grey), seem to occur with different frequencies across transcription and replication states.

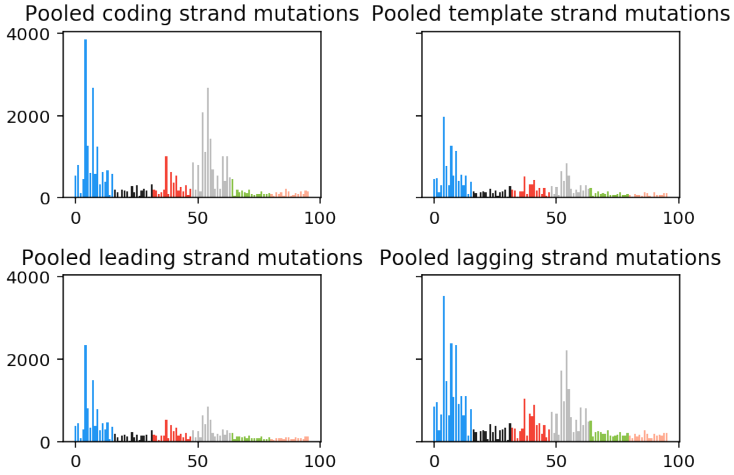

By indexing the SNV tensor appropriately, we can also recover mutational spectra from different state combinations, eg. `snv[0,:,2].sum(axis=(0,1))` would return a $p \times n$ matrix representing the coding strand mutations in state 2 of the first additional genomic dimension.

Understanding tensor factors

In the previous section, we created a simulated dataset using the `TensorSignaturesData` class, and investigated the data by plotting mutational spectra in various genomic contexts. While doing so, we discovered that some variant types occur with different frequencies in different genomic states, for example, frequencies of coding strand C>A, C>T and T>A variants seemed to be twice as large in comparison to corresponding numbers on template strand DNA. Strand asymmetries have been observed for several mutational processes and are often attributed to DNA repair mechanisms. Transcription coupled repair (TCR), for example, actively depletes mutations on template strand DNA in gene encoding regions.

Transcriptional and replicational biases TensorSignatures models variability in mutagenesis due to transcription and replication by

1. extracting separate single base substitution spectra for coding and template strand, and leading and lagging strand DNA
2. fitting a scalar for each signature in context of transcription and replication that quantifies the overall strand asymmetry of single base substitutions (bias matrix `b`)
3. fitting a scalar for each signature that is interpreted as the relative signature activity of signature in transcribed vs untranscribed regions, and early and late replicating regions (activity matrix `a`).

To understand this better, let us first plot the signatures that were used to simulate the counts in `data`.

```
> plt.figure(figsize=(16, 5))
> ts.plot_signatures(data.S.reshape(3, 3, -1, 96, data.rank))
```

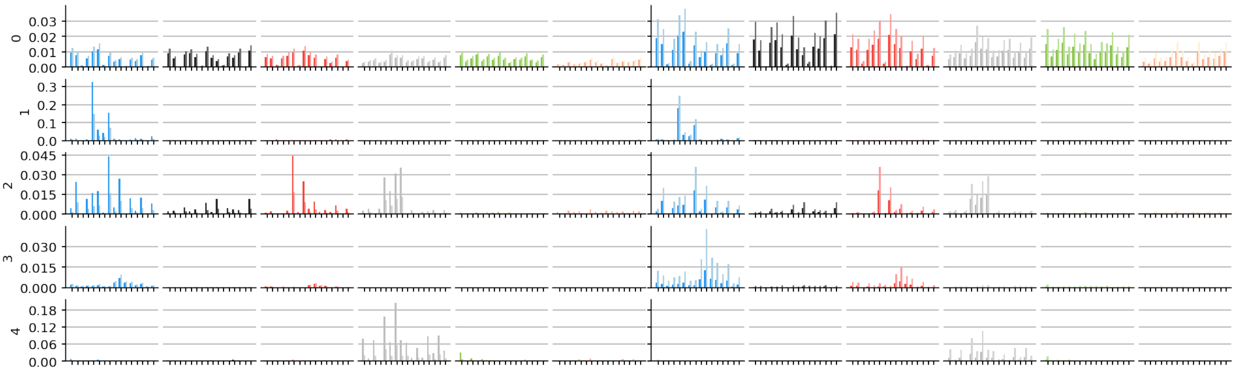

This reveals the SNV spectra (rows) in context of transcription and replication in the left and right column. Colors indicate the mutation type (blue C>A, black C>G, red C>T, grey T>A, green T>C and salmon T>G), while the shading indicates the mutation type probabilities for coding strand and leading strand DNA (dark), and for template and lagging strand DNA (light), respectively. Notice, for example, how in the fourth signature (last row), the amplitude of dark and light grey bars differ, indicating that this mutational process is more likely to produce T>A mutations on coding and leading strand DNA respectively.

TensorSignatures models the propensity of a mutational process to generate strand specific mutations by scaling the SNV spectra for coding and template, and leading and lagging strand with a multiplicative scalar variable. To visualise the strand biases for our simulated dataset, we pass the strand biases, accessible via the `b` attribute of our `data` object, to the `ts.heatmap` function.

```
> plt.figure(figsize=(6,2))
> ts.heatmap(data.b,
             vmin=.5, vmax=2, # allows to specify the limits of the colorbar
             row_labels=['transcription', 'replication'],
             cbarlabel='Strand bias (No bias = 1)' # color bar label
            )
```

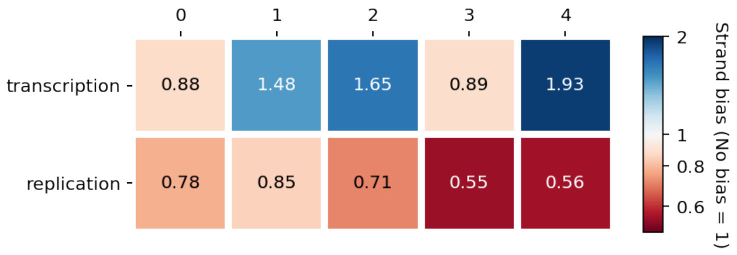

Rows of the heat map depict the context and columns signatures. Note the logarithmic scaling of the color bar, which indicates that a baseline value of 1 resembles a mutational process with no strand preference. Coefficients < 1 (red) indicate signature enrichment on template or lagging strand DNA, and conversely, values > 1 (blue), an asymmetry towards the on coding or leading strand.

Signature activities in specific genomic regions The multidimensional representation of SNV count data allows TensorSignatures to quantify the propensity of mutational processes within

confined genomic regions. These genomic contexts, thereafter also genomic states, may represent genomic features such as specific chromatin marks or nucleosome occupancy. To illustrate this, we depicted a genomic region in the Fig. 1 together with arbitrary genomic states and respective mutations.

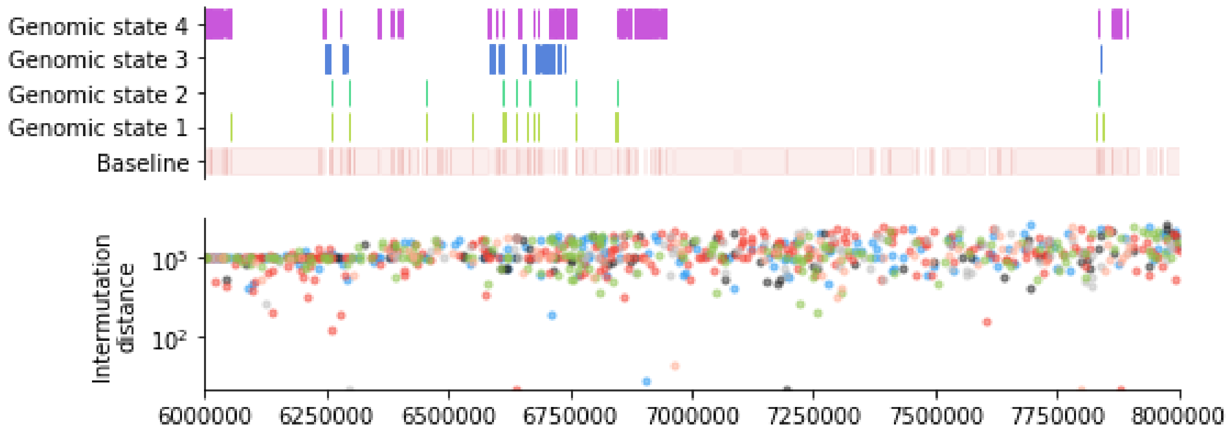

Supplementary Note 2 Fig. 1: **The distribution of single base substitutions may vary due to differences in genome organisation and other factors.** The horizontal bar plot in the upper panel depicts genomic states, which represent confined genomic regions with certain features. The rainfall plot underneath shows the variant types at these genomic loci.

The rainfall plot representation may not always reveal changes in the mutational spectrum on first sight. However, the SNV count tensor contains the mutational spectra of each state combination. We can inspect them by indexing the respective state and summing over all remaining dimensions except the one for trinucleotides (Sec. 3). To visualize, for example, pooled mutation spectra along the five states of the fourth dimension in our simulated dataset we would execute the following code.

```
> fig, ax = plt.subplots(1, 5, figsize=(16, 2.5), sharey=True)
> ax[0].bar(np.arange(96), snv[:, :, :, 0].sum(axis=(0, 1, 2, 4)), \
color=ts.DARK_PALETTE)
> ax[0].set_title('Baseline')
> ax[1].bar(np.arange(96), snv[:, :, :, 1].sum(axis=(0, 1, 2, 4)), \
color=ts.DARK_PALETTE)
> ax[1].set_title('Genomic state 1')
> ax[2].bar(np.arange(96), snv[:, :, :, 2].sum(axis=(0, 1, 2, 4)), \
color=ts.DARK_PALETTE)
> ax[2].set_title('Genomic state 2')
> ax[3].bar(np.arange(96), snv[:, :, :, 3].sum(axis=(0, 1, 2, 4)), \
color=ts.DARK_PALETTE)
> ax[3].set_title('Genomic state 3')
> ax[4].bar(np.arange(96), snv[:, :, :, 4].sum(axis=(0, 1, 2, 4)), \
color=ts.DARK_PALETTE)
> ax[4].set_title('Genomic state 4')
```

This plot nicely illustrates that different genomic states may have a variable exposure to different mutational signatures. For example, judging from the prevalence of C>A and T>A variants in

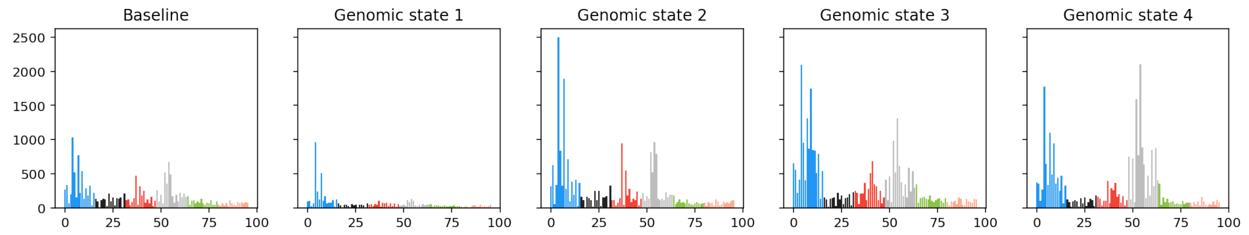

genomic state 2 and 4, it appears likely that these states are dominated by signature 3 and 4 respectively. TensorSignatures models the activity of each signature by fitting a single coefficient for each signature and genomic state. To visualize the coefficients used to generate our simulated dataset we execute

```
> plt.figure(figsize=(6,2))
> ts.heatmap(data.k1,
           row_labels=['Genomic state 1', ..., 'Genomic state 4', ],
           col_labels=['{}'.format(i) for i in range(5)],
           cbarlabel='Relative Signature\nactivity (Baseline = 1)')
```

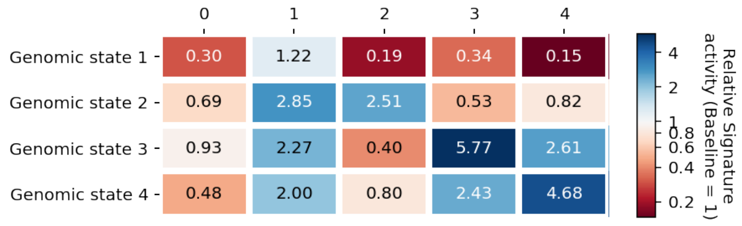

which confirms our suspicion about the elevated activities of signature 3 and 4 in genomic state 3 and 4 respectively. To interprete this correctly, keep in mind that usually majority of SNVs do not fall into specific genomic states and therefore end up in the baseline or "unassigned" state (Tab. 1), which is in TensorSignatures always 1, and to which all other coefficients are inferred relatively to. In other words, signature 3, for example, shows 5.77 times higher activities in genomic state 3 in comparison to the genomic baseline.

The TensorSignatures CLI

The TensorSignatures CLI comes with six subroutines,

- `boot`: computes bootstrap intervals for a TensorSignature initialisation,
- `data`: simulates mutation count data for a TensorSignature inference,
- `prep`: computes a normalisation constant and formats a count tensor (Sec. 3),
- `refit`: refits the exposures to set of fixed tensor signatures (Sec. 3),
- `train`: runs a denovo extraction of tensor signatures (Sec. 3),
- `write`: creates a hdf5 file out of dumped tensor signatures pkls.

The goal of this tutorial is to illustrate how to run TensorSignatures in a practical setting. For this reason we will first simulate mutation count data using `tensorsignatures data`, and subsequently run `tensorsignatures train` to extract constituent signatures. In the next section we will then analyse the results of this experiment in jupyter with help of the tensorsignatures API (Sec. 3).

Simulate data via the CLI To create a reproducible (the first positional argument sets a seed: 573) synthetic dataset from 5 mutational signatures (second positional argument) with the CLI, we invoke the data subprogram

```
$ tensorsignatures data 573 5 data.h5 -s 100 -m 10000 -d 3 -d 5
```

which will simulate 100 samples (`-s 100`) each with 10,000 mutations (`-m 10000`), and two additional genomic dimensions with 3 and 5 states (`-d 3 -d 5`) respectively. The program writes a `hdf5` file `data.h5` to the current folder containing the datasets `SNV` and `OTHER` representing the SNV count tensor and all other variants respectively.

Running TensorSignatures using the command line interface Since we know the number of signatures that made up the dataset we can run a TensorSignatures decomposition simply by executing

```
$ tensorsignatures --verbose train data.h5 my_first_run.pkl 5
```

which saves a pickle able binary file to the disk, which we can load into a interactive python session (eg. a Jupyter notebook) for further investigation (see Sec. 3)

```
> init = ts.load_dump('my_first_run.pkl')
> init.S.shape
(3, 3, 3, 5, 96, 5, 1)
```

However, usually we do not know the number of active mutational processes a priori. For this reason, it is necessary to run the algorithm using different decomposition ranks, and to subsequently select the most appropriate model for the data. Moreover, we recommend to run several initialisations of the algorithm at each decomposition rank. This is necessary, because non-negative matrix factorisation produces stochastic solutions, i.e. each decomposition represents a local minimum of the objective function that is used to train the model. As a result, it is worthwhile to sample the solution space thoroughly, and to pick the solution which maximised the log-likelihood. Running TensorSignatures at different decomposition ranks while computing several initialisations is easy using the CLI. For example, to compute decompositions from rank 2 to 10 with 10 initialisation each, we would simply write a nested bash loop.

```
$ for rank in {2..10}; do
$   for init in {0..9}; do
$     tensorsignatures train data.h5 sol_${rank}_${init}.pkl ${rank} \
-i ${init} -j MyFirstExperiment;
$   done;
$ done;
```

Also note the additional arguments we pass here to the programme; the `-i` argument identifies each initialisation uniquely (mandatory), and the `-j` parameter allows us to name the experiment, which in this context denotes multiple TensorSignature decompositions across a range of ranks extracted using the same hyper parameters (number of epochs, dispersion, etc).

Summarising the result from many initialisations with `tensorsignatures write` This command produces for each rank (2-10) ten initialisation and saves the results as pickleable binary files to the hard disk. Loading the 9 x 10 initialisations manually using `ts.load_dump` would be quite tedious and even impracticable in larger experiments. For this reason, we included the subprogram `tensorsignatures write`, which takes a `glob` filename pattern and an output filename as arguments to generate a `hdf5` file containing all initialisations.

```
$ tensorsignatures write "sol_*.pkl" results.h5
Processing 90 files.
```

The TensorSignatures API

The TensorSignatures API provides useful functions to analyse results from TensorSignature decompositions. Since running the tool usually involves creating several initialisations at different decomposition ranks (Sec. 3), we provide three classes that abstract

- Experiments (`Experiment`), i.e. multiple initialisation at different decomposition ranks extracted using the same hyper parameters,
- Cluster (`Cluster`), i.e. multiple initialisations at a specific decomposition rank,
- Initialisations (`Initialization`): a single decomposition.

Importing data and performing model selection using the `Experiment` class The `Experiment` class loads and clusters initialisations of each decomposition rank of a `hdf5` file written by `tensorsignatures write` (Sec. 3)

```
> experiment = ts.Experiment("results.h5")
```

The `data` field of an `Experiment` instance returns a set of keys, which allow us to access the `Cluster` of the experiment (Sec. 3). Keys follow the format to prefix the decomposition rank with the name of the experiment, which we set earlier using the `-j` flag when we ran `tensorsignatures train` (Sec. 3).

```
> experiment.data
{'/MyFirstExperiment/10', '/MyFirstExperiment/2', ..., \
'/MyFirstExperiment/9'}
```

The `Experiment` class computes a table of useful statistics,

```
> experiment.summary_table.head()
```

which, for example, enable us to inspect log likelihood of each initialisation[2],

```
> sns.swarmplot(x='rank', y='log_L', hue='init', \
data=experiment.summary_table, color='C0', palette='deep')
```

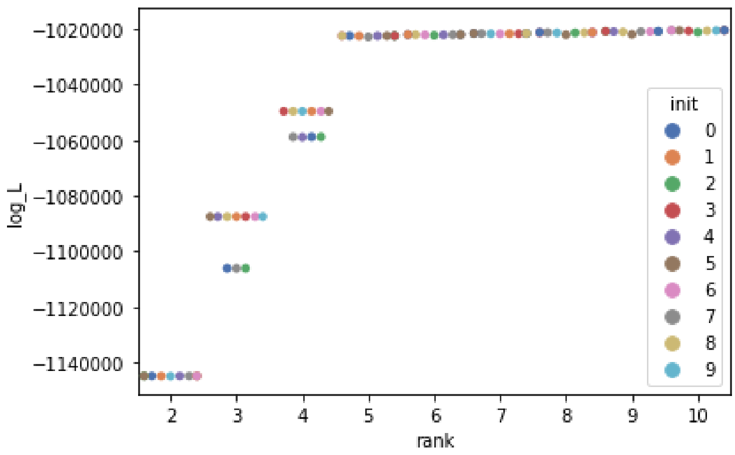

The summary_table also allows us to perform model selection using the Bayesian Information Criterion (BIC). This estimator tries to find a trade-off between the log-likelihood and the number of parameters in the model; chosen is the rank which minimises the BIC. To understand which model to choose in our experiment, we will quickly plot the rank against BIC,

```
> import seaborn as sns
> sns.boxplot(x='rank', y='BIC', data=experiment.summary_table, color='C0')
```

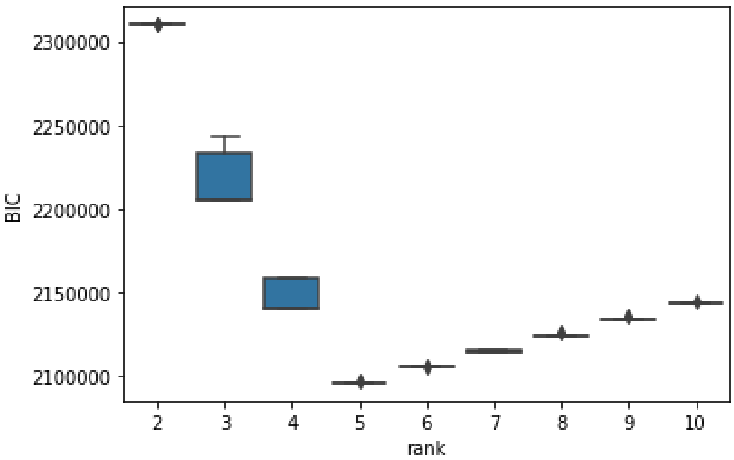

indicating that rank 5 is most appropriate for our dataset.

The `Cluster` class wraps multiple TensorSignature initialisations We can extract the cluster of a specific decomposition rank by passing these keys to the getter function of an `Experiment` object. For example, to extract the rank 5 solution, we execute

```
> cluster = experiment['/MyFirstExperiment/5']
```

A `Cluster` instance is essentially a wrapper for multiple `Initializations` (Sec. 3). It embodies attributes to access the parameters of a tensor signature inference, for example, we may access the extracted signature tensor(s) through the `S` field of `Cluster`.

[2] Here we use the the `seaborn` library to create the plot. You can install the package, if necessary, by executing `pip install seaborn` in your terminal.

```
> cluster.S.shape
(3, 3, 3, 5, 96, 5, 10)
```

Note the similarity between the shape of the extracted signature tensor and the shape of the input `snv` count tensor (`(3, 3, 3, 5, 96, 100)`). First few indices match the size of corresponding genomic dimensions, i.e. transcription and replication directionality (each 3), genomic dimension 1 and 2 (3 and 5) and single base substitution types (96). The following two indices, however, indicate the decomposition rank (5) rather than the number of samples, and the number initialisations in the cluster.
Other model parameters may be accessed through the following fields:

- Other mutation type signatures: `result.T`
- Exposures: `result.E`
- Transcription and replicational strand biases: `result.b`
- Signature activities in transcribed/untranscribed regions and early/late replicating regions: `result.a`
- Arbitrary genomic property (like epigenetic signature activities): `result.k0`, `result.k1`, ..., `result.kx`
- Mixing proportions: `result.m`

The last dimension of an extracted Cluster parameter always indicates the number of available initialisations. To extract the solution of a particular initialisation, we can can simply index it using standard `numpy` indexing. Here we make use of the so called ellipsis operator (`...`) which enables to index the last dimension of an multidimensional array

```
> solution = cluster.b[..., 3]
> solution.shape
(2, 7)
```

`Cluster` objects provide an `init` field containing the index of the initialisation with the highest log-likelihood. To extract this particular `Initialization` from a cluster, we simply pass it to the `Cluster` getter function.

```
> init = cluster[cluster.init]
```

A `Initialization` object stores tensor signatures, factors and exposures Similar to TensorSignature `Cluster` objects, `Initializations` contain the fitted model including all parameters. For example, we can access the extracted signature tensor by accessing the `S` field from `init`.

```
> init.S.shape
(3, 3, 3, 5, 96, 5, 1)
```

Note that the last dimension of S has a size of one, indicating an initialisation rather than a clustered signature tensor. Two other useful methods of `Initialization` objects are `to_dic` and `dump`, which let us serialise and save the result of a TensorSignature initialisation to the hard disk.

```
> # returns a dictionary with all parameters
> init.to_dic()
> # saves the initialisation to disk (load a saved solution with \
ts.load_dump)
> init.dump('initialisation.pkl')
```

The TensorSignatures API features some basic plotting function which allow us to visualise the extracted parameters of an `Initialisation`.

- `plot_signatures`: plots single base substitution spectra in context of transcription and replication
- `heatmap`: plots tensor factors (transcription and replication biases (`b`), signature activities (`a`), and genomic activities (`k0`, `k1`, `...`, `kx`))

The `ts.plot_signatures` function expects an 5 dimensional array `(3, 3, -1, 96, rank)`. Due to the fact that we can have an arbitrary number of genomic states, we first have to reshape the signature tensor before we can pass it to the plotting function.

```
> plt.figure(figsize=(16, 5))
> ts.plot_signatures(init.S.reshape(3, 3, -1, 96, init.rank))
```

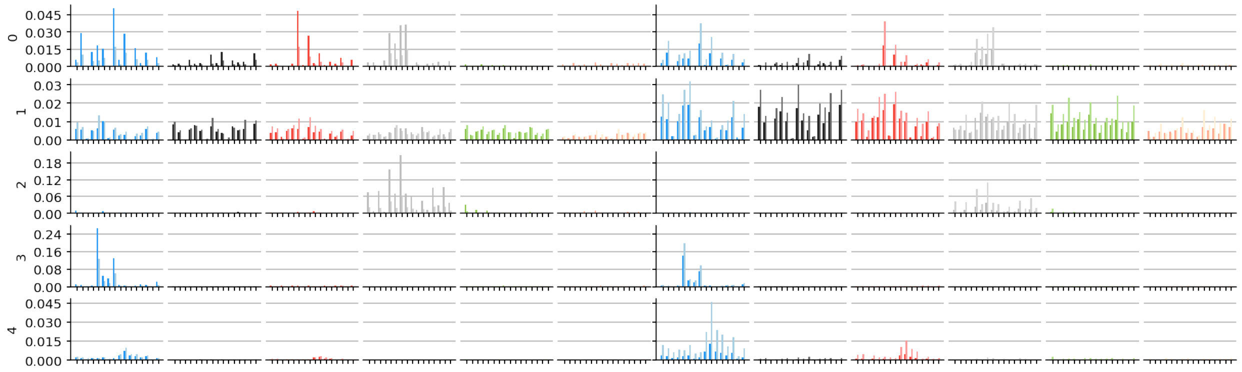

We can plot extracted tensor factors `result.b`, `result.a`, `result.k0` and `result.k1` using the `ts.heatmap` function. Note, that similarly to the the signature tensor, the `Initialization` object appends an additional dimension to indicate its index. For this reason, we need to reshape the arrays containing tensor factors or index them appropriately.

```
> result.b.shape # transcription and replication strand biases
(2, 5, 1)
> # ... (the elipsis operator) allows to index the last dimension of an array
> plt.figure(figsize=(6, 2))
> ts.heatmap(result.b[..., 0],
```

```
    vmin=.5, vmax=2,
    row_labels=['transcription', 'replication'],
    col_labels=['{}'.format(i) for i in range(5)],
    cbarlabel='Strand bias (No bias = 1)' # color bar label
   )
```

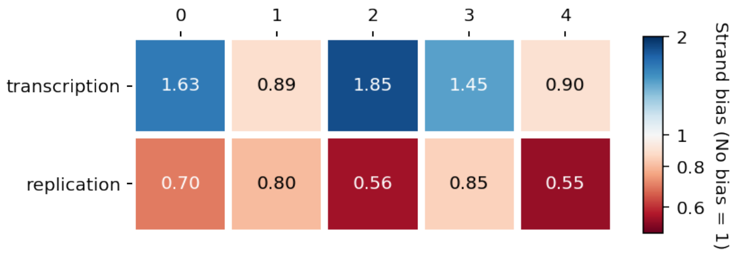

Exercise: Compare how extracted parameters differ from the ground truth (Sec. 3).

Running TensorSignatures through the API In some scenarios it might be desirable to run TensorSignatures via the API rather than the CLI (for example when integrating TensorSignatures into custom pipelines). To illustrate this, we first simulate data and extract the SNV count tensor and the matrix containing other mutation types. Here it is important to notice that the sample dimensions have to match, e.g. `snv[..., 4]` has to match `other[..., 4]`.

```
> data = ts.TensorSignatureData(
    seed=573, # set a seed for reproducibility
    rank=5, # number of signatures
    samples=100, # number of samples
    dimensions=[3, 5], # number of arbitrary genomic dimensions
    mutations=1000)
> snv = data_set.snv() # the SNV count tensor (3, 3, 3, 5, 96, 100)
> other = data_set.other() # other mutation type matrix (234, 100)
```

The next step is to pass the desired decomposition rank, as well as the input data, i.e. the `snv` count tensor and the `other` mutation matrix, to the `TensorSignature` class[3]. The `TensorSignature` constructor also receives other model hyperparameters such as learning rate of the model or the number of epochs to train the model. By default, TensorSignatures uses the outlier robust negative binomial distribution with a dispersion $\tau = 50$ to model the mutation count, and trains the model for 10,000 epochs.

```
# perform a rank 5 decomposition
model = ts.TensorSignature(snv, other, rank=5, verbose=True, epochs=20000)
```

To fit the signatures to our data, we simply invoke the `fit` method of the `model` instance, which will return a `Initialization` object after finishing to train the model.

```
> result = model.fit()
```

[3]When working with real genomic data it is also necessary to pass a normalisation tensor (via the argument `N`) which accounts for differences in the nucleotide composition of different genomic regions to the `TensorSignature` constructor.
