# Supplementary material for: Learning mutational signatures and their multidimensional genomic properties with TensorSignatures
Source: Nat Commun. 2021 Jun 15;12:3628. doi: 10.1038/s41467-021-23551-9 (PMC8206343; doi:10.1038/s41467-021-23551-9)
Supplement: Supplementary file 3 — Reporting Summary [file 41467_2021_23551_MOESM3_ESM.pdf]

nature research

Corresponding author(s): Moritz Gerstung

Last updated by author(s): Feb 22, 2021

Reporting Summary

Nature Research wishes to improve the reproducibility of the work that we publish. This form provides structure for consistency and transparency in reporting. For further information on Nature Research policies, see Authors & Referees and the Editorial Policy Checklist .

Statistics

For all statistical analyses, confirm that the following items are present in the figure legend, table legend, main text, or Methods section.

n/a	Confirmed	
[ ]	[x]	The exact sample size (*n*) for each experimental group/condition, given as a discrete number and unit of measurement
[x]	[ ]	A statement on whether measurements were taken from distinct samples or whether the same sample was measured repeatedly
[ ]	[x]	The statistical test(s) used AND whether they are one- or two-sided<br>*Only common tests should be described solely by name; describe more complex techniques in the Methods section.*
[ ]	[x]	A description of all covariates tested
[ ]	[x]	A description of any assumptions or corrections, such as tests of normality and adjustment for multiple comparisons
[ ]	[x]	A full description of the statistical parameters including central tendency (e.g. means) or other basic estimates (e.g. regression coefficient) AND variation (e.g. standard deviation) or associated estimates of uncertainty (e.g. confidence intervals)
[x]	[ ]	For null hypothesis testing, the test statistic (e.g. *F*, *t*, *r*) with confidence intervals, effect sizes, degrees of freedom and *P* value noted<br>*Give P values as exact values whenever suitable.*
[x]	[ ]	For Bayesian analysis, information on the choice of priors and Markov chain Monte Carlo settings
[x]	[ ]	For hierarchical and complex designs, identification of the appropriate level for tests and full reporting of outcomes
[ ]	[x]	Estimates of effect sizes (e.g. Cohen's *d*, Pearson's *r*), indicating how they were calculated

Our web collection on statistics for biologists contains articles on many of the points above.

Software and code

Policy information about availability of computer code

Data collection	No software was used for data collection.
Data analysis	Python 3.6 (Packages: h5py (2.7.1), ipython (6.3.1), matplotlib (3.0.2), numpy (1.16.1), pandas (0.22.0), scikit-learn (0.19.1), scipy (1.0.1), seaborn (0.9.0), scipy (1.0.1), tensorflow (1.4.1), tensorsignatures (0.4.0), tqdm (4.28.1),),<br>R 3.4 (Packages: BiocInstaller/Bioconductor (1.24.0), Biostrings (2.42.1), BSgenome (1.42.0), GenomicRanges (1.26.4), VariantAnnotation (1.20.3), rhdf5 (2.18.0))<br>The TensorSignatures algorithm is publicly available at https://github.com/gerstung-lab/tensorsignatures

For manuscripts utilizing custom algorithms or software that are central to the research but not yet described in published literature, software must be made available to editors/reviewers. We strongly encourage code deposition in a community repository (e.g. GitHub). See the Nature Research guidelines for submitting code & software for further information.

Data

Policy information about availability of data

All manuscripts must include a data availability statement. This statement should provide the following information, where applicable:

- Accession codes, unique identifiers, or web links for publicly available datasets
- A list of figures that have associated raw data
- A description of any restrictions on data availability

The PCAWG data is available under restricted access, access can be obtained at http:// dcc.icgc.org/pcawg.
The HMF data is available under restricted access, access can be ob- tained at https://www.hartwigmedicalfoundation.nl/applying-for-data/.
The XPC genome data is available under restricted access, access can be obtained at https://www.ncbi. nlm.nih.gov/projects/gap/cgi-bin/study.cgi?study_id=phs000830.v1.p1.
The transcription data used in this study are available at https://www.gencodegenes.org/human/ release_19.html.
The replication data used in this study are available at https://www. encodeproject.org/search/?type=Experiment&assay_title=Repli-seq.

The ChromHMM data used in this study are available at https://egg2.wustl.edu/roadmap/web_portal/ chr_state_learning.html.
The nucleosome MNase dataset used in this study are avail- able at https://egg2.wustl.edu/roadmap/web_portal/chr_state_learning.html.

Field-specific reporting

Please select the one below that is the best fit for your research. If you are not sure, read the appropriate sections before making your selection.

☒ Life sciences ☐ Behavioural & social sciences ☐ Ecological, evolutionary & environmental sciences

For a reference copy of the document with all sections, see nature.com/documents/nr-reporting-summary-flat.pdf

Life sciences study design

All studies must disclose on these points even when the disclosure is negative.

Sample size	2,778 cancer genomes from the PCAWG consortium + 3,824 metastatic cancer genomes from the Hartwig Medical Foundation.
Data exclusions	No further exclusions were applied to the data.
Replication	The results of our discovery analysis in the PCAWG dataset was validated in an independent dataset comprising 3,824 metastatic cancer genomes from the Hartwig Medical foundation. Further, the statistical robustness of the inference method was assessed using bootstrap resampling.
Randomization	Randomization is not applicable to our study (the experimental design does not involve a randomized control trial or a paired test design).
Blinding	Blinding is not applicable to our study (the experimental does not include a randomized control trial).

Reporting for specific materials, systems and methods

We require information from authors about some types of materials, experimental systems and methods used in many studies. Here, indicate whether each material, system or method listed is relevant to your study. If you are not sure if a list item applies to your research, read the appropriate section before selecting a response.

Materials & experimental systems

n/a	Involved in the study	
☒	☐	Antibodies
☒	☐	Eukaryotic cell lines
☒	☐	Palaeontology
☒	☐	Animals and other organisms
☒	☐	Human research participants
☒	☐	Clinical data

Methods

n/a	Involved in the study	
☒	☐	ChIP-seq
☒	☐	Flow cytometry
☒	☐	MRI-based neuroimaging
